# Comparative Analyses of Single-Cell Transcriptomic Profiles between In Vitro Totipotent Blastomere-like Cells and In Vivo Early Mouse Embryonic Cells

**DOI:** 10.3390/cells10113111

**Published:** 2021-11-10

**Authors:** Po-Yu Lin, Denny Yang, Chi-Hsuan Chuang, Hsuan Lin, Wei-Ju Chen, Chia-Ying Chen, Trees-Juen Chuang, Chien-Ying Lai, Long-Yuan Li, Scott C. Schuyler, Frank Leigh Lu, Yu-Chuan Liu, Jean Lu

**Affiliations:** 1 Genomics Research Center, Academia Sinica, Taipei 11529, Taiwan; adam80829@gmail.com (P.-Y.L.); tze.yang19@imperial.ac.uk (D.Y.); jubie548888tw@gmail.com (C.-H.C.); chloechen1026@gmail.com (W.-J.C.); cychen0704@gate.sinica.edu.tw (C.-Y.C.); trees@gate.sinica.edu.tw (T.-J.C.); 2 Taiwan International Graduate Program, Molecular and Cell Biology, Academia Sinica and National Defense Medical Center, Taipei 11529, Taiwan; 3 School of Medicine, Imperial College London, London SW7 2AZ, UK; 4 Taiwan International Graduate Program in Molecular Medicine, National Yang-Ming Chiao-Tung University, Academia Sinica, Taipei 100147, Taiwan; johnnylin0917@gm.ym.edu.tw; 5 Biomedical Translation Research Center, Academia Sinica, Taipei 11529, Taiwan; believe614@gate.sinica.edu.tw (C.-Y.L.); melonliu@gate.sinica.edu.tw (Y.-C.L.); 6 Department of Life Sciences, National Chung Hsing University, Taichung 40227, Taiwan; lyuan@dragon.nchu.edu.tw; 7 Department of Biomedical Sciences, College of Medicine, Chang Gung University, Taoyuan 33302, Taiwan; schuyler@mail.cgu.edu.tw; 8 Division of Head and Neck Surgery, Department of Otolaryngology, Chang Gung Memorial Hospital, Taoyuan 33302, Taiwan; 9 Department of Pediatrics, National Taiwan University Children’s Hospital, National Taiwan University Hospital, National Taiwan University Medical College, Taipei 100, Taiwan; frankllu@ntu.edu.tw; 10 Department of Life Science, Tzu Chi University, Hualien 97071, Taiwan; 11 Graduate Institute of Medical Sciences, National Defense Medical Center, Taipei 11490, Taiwan; 12 National Core Facility Program for Biotechnology, National RNAi Platform, Taipei 11529, Taiwan

**Keywords:** totipotency, embryonic stem cells, totipotent blastomere-like cells, 2-cell-like cells (2CLCs), spliceosome inhibitor, pladienolide B, *Zscan4*

## Abstract

The developmental potential within pluripotent cells in the canonical model is restricted to embryonic tissues, whereas totipotent cells can differentiate into both embryonic and extraembryonic tissues. Currently, the ability to culture in vitro totipotent cells possessing molecular and functional features like those of an early embryo in vivo has been a challenge. Recently, it was reported that treatment with a single spliceosome inhibitor, pladienolide B (plaB), can successfully reprogram mouse pluripotent stem cells into totipotent blastomere-like cells (TBLCs) in vitro. The TBLCs exhibited totipotency transcriptionally and acquired expanded developmental potential with the ability to yield various embryonic and extraembryonic tissues that may be employed as novel mouse developmental cell models. However, it is disputed whether TBLCs are ‘true’ totipotent stem cells equivalent to in vivo two-cell stage embryos. To address this question, single-cell RNA sequencing was applied to TBLCs and cells from early mouse embryonic developmental stages and the data were integrated using canonical correlation analyses. Differential expression analyses were performed between TBLCs and multi-embryonic cell stages to identify differentially expressed genes. Remarkably, a subpopulation within the TBLCs population expressed a high level of the totipotent-related genes *Zscan4s* and displayed transcriptomic features similar to mouse two-cell stage embryonic cells. This study underscores the subtle differences between in vitro derived TBLCs and in vivo mouse early developmental cell stages at the single-cell transcriptomic level. Our study has identified a new experimental model for stem cell biology, namely ‘cluster 3’, as a subpopulation of TBLCs that can be molecularly defined as near totipotent cells.

## 1. Introduction

Life starts from a single fertilized egg, then proliferates into two, four, eight, and sixteen cells, and then into blastocysts. Two cell blastomeres, but not the later blastocyst cells, are considered totipotent. There are two distinct definitions of totipotency. The stringent definition of totipotency is the ability of a single cell to develop into an entire organism. In regard to the stringent definition, Tarkowski et al. destroyed one of the blastomeres from a two-cell stage mouse embryo inside the pellucid zone [1]. The remaining single blastomere was subsequently transferred to a surrogate mother, from which a newborn was developed to term. This experiment was the first to prove that individual two-cell blastomere cells have the developmental potential equivalent to a zygote, both of which are currently considered as totipotent. A more lenient definition of totipotency are cells possessing the ability to form chimeras and contribute to both embryonic and extraembryonic tissues [2,3,4]. For the more lenient definition, interestingly, a recent study identified a transient subpopulation within mouse pluripotent stem cell (PSC) cultures that share totipotent features and developmental potential equivalent to the two-cell stage embryonic cells, which are referred to as ‘2-cell-like cells’ (2CLCs). The 2CLCs express totipotent genes including the *MERVL* retrotransposon and exhibit the downregulation of pluripotent-specific genes that are robustly expressed in PSCs such as *Pou5f1*. The 2CLCs have provided an in vitro cell culture model for further studies on cellular totipotency [5,6]. However, 2CLCs cannot be stably maintained under in vitro culturing conditions [7].

In the very first stages of the mouse embryo, the cell originating from the oocyte is transcriptionally inactive. Many maternal mRNAs and proteins are stored in the oocyte, and they play essential roles in subsequent embryonic development [8]. After fertilization, the oocyte rapidly forms the zygote (1-cell stage) within one day. Following zygote formation, a major wave of zygotic gene activation (ZGA) begins to occur in the late two-cell stage [9,10]. ZGA is the significant event that triggers embryonic mRNA expression and the production of embryonic proteins and significantly controls the embryonic development. There are approximately 3000 genes activated during the ZGA wave of expression [10]. Among the various kinds of genes that are expressed during the ZGA wave in the late two-cell stage mouse embryo, the transcription factor zinc finger and SCAN domain containing 4 (*Zscan4*) is a totipotency marker that was found to be enriched in late two-cell mouse embryos and 2CLCs [11,12]. Many studies have reported that approximately 5% of mouse embryonic stem cells (mESCs) express *Zscan4* [5]. *Zscan4* is known to improve the normality of karyotypes, expand developmental potential, and promote telomere length maintenance throughout early embryonic development [13]. The reduction of *Zscan4* expression delays the development of two-cell stage mouse preimplantation embryos [11]. *Zscan4* is also essential for the development of functional blastocysts at the preimplantation stage [11].

**Figure 1:**
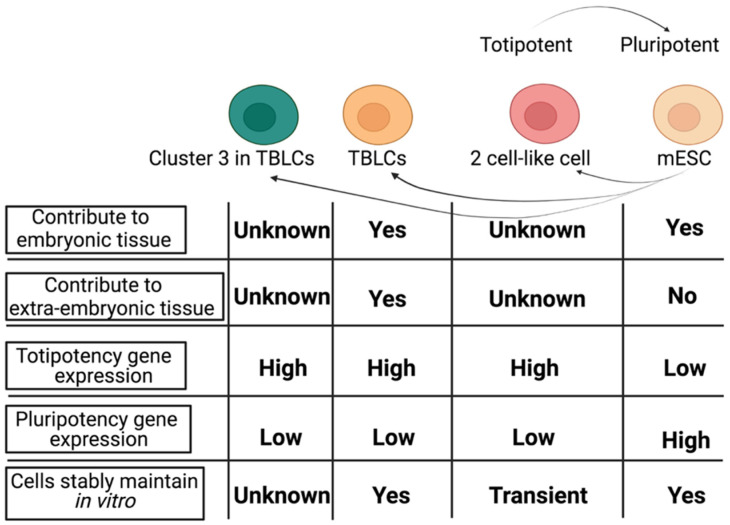
Comparison of molecular and functional features between ESCs, TBLCs, cluster 3 TBLCs, and 2-cell-like cells.

A recent study reported that applying a single spliceosome inhibitor, pladienolide B (PlaB), to mESC cultures in vitro could successfully induce the upregulation of totipotent blastomere-like cells (TBLCs) totipotency gene markers including *Zscan4s* and *MERVL* [14]. At the same time, the expression of pluripotent factors such as *Pou5f1*, *Nanog*, and *Sox2* were downregulated as compared to mESCs. Transcriptomic analyses revealed that TBLCs are similar to two- and four-cell blastomeres in vitro. Remarkably, chimeric assays showed that a single TBLC can contribute to the E6.5 to E7.5 embryo, including cells in the epiblast, extraembryonic ectoderm, and the ectoplacental cone tissues. Overall, TBLCs have been shown to display several totipotent features molecularly and functionally. Figure 1 displays features of multiple cell types including 2CLC, mESCs, and TBLCs (Figure 1).

**Figure 2:**
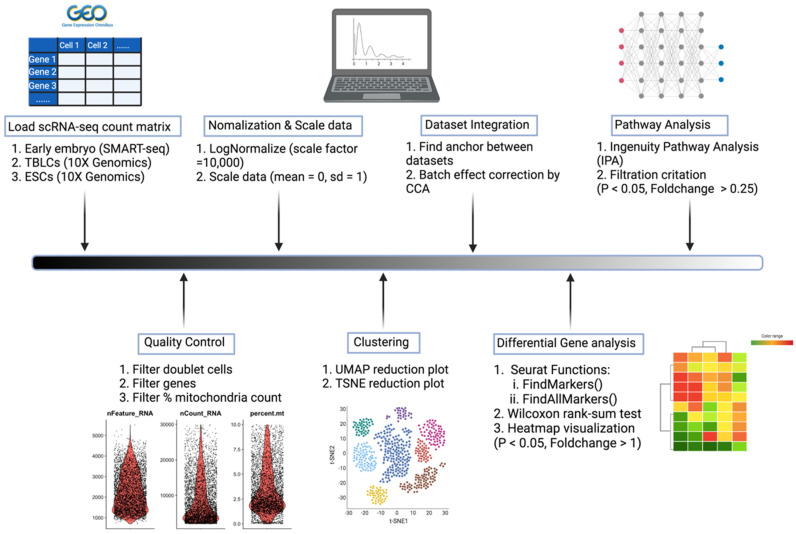
Workflow of single-cell RNA sequencing data analyses.

Here, we analyzed single cell RNAs to dissect the transcriptomic features of TBLCs by comparing them to early mouse embryonic developmental cell stages (Figure 2). This was achieved by characterizing differential expression patterns between TBLCs and multi-cell stages in the early embryo. Potential genes that regulate the totipotency and developmental potential of TBLCs were identified and ontologized. Interestingly, a subpopulation called ‘cluster 3′, that accounts for 16.9% of the TBLCs cellular population, was identified in low-dimensional space in which there is an enrichment of totipotent gene markers such as *Zscan4s, Sp110*, and the *Gm* protein family. (Figure 1). This unique cluster 3 in TBLCs displays a transcriptome profile similar to two-cell stage mouse embryos in vivo. Our analyses clarify at the molecular and cellular level the current unclear relationship between the *Zscan4*-positive TBLCs subpopulation in vitro and the mouse early embryonic stages in vivo.

## 2. Materials and Methods

### 2.1. Single-Cell RNA Sequencing (scRNA-Seq) Dataset Sources

This study utilized published data of mouse TBLCs, naïve ESCs, and preimplantation embryos to perform comparative transcriptomic analyses. The scRNA-seq count matrix of TBLCs were downloaded from the Gene Expression Omnibus (GEO) website (GSE168728). This dataset is derived from TBLCs on feeder cells (MEF) after 6 passages following 2.5 nM pladienolide B (PlaB) treatment. The scRNA-seq count matrices of mouse naïve ESCs were downloaded from the same source (GSE168728). The mouse preimplantation embryo count matrices were downloaded from another study (GSE45719). This dataset consists of mouse early developmental stages ranging from zygotes to late blastocysts.

### 2.2. scRNA-Seq Analyses

The Seurat (Satija Lab, New York, NY, USA)(4.0.3) R package was used for the scRNA-seq analysis workflow unless otherwise stated. For quality control, we used the same parameters published by the previous paper for further analyses [14]. TBLCs with more than 2000 and less than 30,000 gene read counts were selected, while ESCs were filtered with 4000 < read count < 40,000. All cells with less than 10% of the mitochondrial genes were selected for further analyses except ESCs which were filtered with 5% < mitochondrial genes. The number of cells remaining after quality control filtration was: TBLCs = 4534 cells, early development = 259 cells, and ESCs = 4139 cells. After filtering out low-quality cells, data were normalized with the ‘NormalizeData’ function in which the feature expressions of each cell were normalized by the total gene expression, multiplied by a scale factor of 10,000, and finally each feature of the gene expression was log-transformed. Gene expression levels of the top 2000 variable genes were linearly transformed with the ‘ScaleData’ function before dimensional reduction. Principal component analysis (PCA) was performed on linear transformed data with the ‘RunPCA’ function. The top principal components (PCs) with high variance (>4) and low *p*-value (<0.05) were selected to first construct the K-nearest neighbor graph using the ‘FindNeighbors’ function, where edges were drawn between any two-cells with similarly expressed genes. Unsupervised cell clustering was performed by applying Louvain’s modularity optimization algorithm with the ‘FindClusters’ function. Using the same number of PCs included in the clustering analyses, t-SNE or UMAP nonlinear dimensional reduction techniques were performed by the ‘RunTSNE’ and ‘RunUMAP’ functions, respectively. Single-cell data were merged using the ‘Merge’ function; dataset integrations and batch corrections were performed with the ‘FindIntegrationAnchors’ (canonical correlation analysis) and ‘IntegrateData’ functions, respectively. Differentially expressed genes between cell clusters were calculated using the Wilcoxon rank sum test with the ‘FindMarkers/FindAllMarkers’ function, and the identified differentially expressed genes with Bonferroni-corrected *p*-values < 0.05 were selected.

To visualize featured gene expression patterns on t-SNE or UMAP plots, the ‘FeaturePlot’ function was used. The ‘VlnPlot’ function was used to visualize the probability distributions of selected gene expression patterns between defined cell clusters. The ‘AverageExpression’ function was used to calculate average gene expression levels for each assigned cell cluster. Using the average gene expression patterns, heatmaps were plotted to compare the top upregulated or downregulated genes between each assigned cell cluster with the ‘DoHeatmap’ function. Only differentially expressed genes with Bonferroni adjusted *p*-values less than 0.05 and fold changes with absolute values greater than 1 were used to plot heatmaps. Correlations between defined cell clusters were determined by the ‘Cor’ function using the Pearson correlation coefficient method. Subsequently, correlation heatmaps were created using the ‘ggplot’ function in ggplot2 (3.3.5) R package to visualize the correlations between each defined cell cluster based on the top 20 PCs or at the global transcriptomic level.

### 2.3. Principal Component Analysis and Unsupervised Hierarchical Clustering Analysis

PCA for each defined cell cluster was calculated with the ‘prcomp’ function. Before PCA plotting, hierarchical clustering analyses were performed using the ‘dist’ and ‘hclust’ functions. To construct 3D PCA plots of the first 3 PCs, the ‘plot3d’ function was used from the rgl (0.107.12) R package. Hierarchical clustering trees of defined cell clusters were generated based on the first 20 PCs or at the global transcriptomic level using the ‘BuildClusterTree’ and ‘PlotClusterTree’ functions in Seurat (4.0.3) R packages.

### 2.4. Ingenuity Pathway Analysis (IPA)

Differential gene analysis data were inputted into the IPA core analysis program. The consultation measurements were searched from twenty-nine various database libraries such as KEGG, Affymetrix, dbSNP, and GenBank. All DE genes used for IPA analyses follow these filtration criteria: Bonferonni adjusted *p*-value less than 0.05, fold change with absolute value greater than 0.25. The IPA software are designed by QIAGEN (Germantown State, MA, USA).

## 3. Results

### 3.1. Transcriptomic Profile Comparisons between Early Mouse Developmental Stages and Tblcs

**Figure 3:**
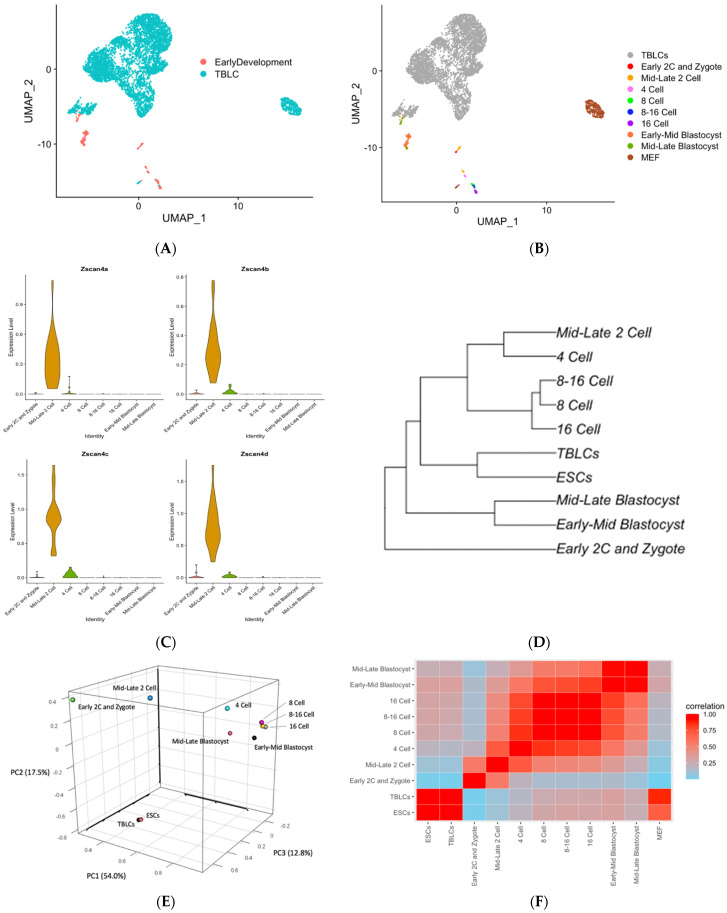
Transcriptomic profile comparisons between mouse early embryonic developmental stages and TBLCs. (**A**) UMAP dimensional reduction plot of mouse early embryonic developmental stages and TBLCs were separated by original identity. (**B**) UMAP dimensional reduction plot of early embryonic developmental stages of mouse cell clusters and TBLCs. (**C**) Violin plots displaying the expression profiles of *Zscan4a*–*d* across different stages of early mouse embryonic development clusters. The *y*-axis scale represents log-transformed gene expression levels. (**D**) Hierarchical clustering tree of TBLCs, ESCs, and early mouse embryonic developmental stages based on global transcriptomic levels. (**E**) Principal component analysis (PCA) of TBLCs, ESCs, and early mouse embryonic developmental stages using the average of single-cell data shown in the first 3 PCs. (**F**) A correlation matrix of TBLCs, ESCs, and early mouse embryonic developmental stages at the global transcriptomic level using Pearson’s correlation coefficient method. Scale bar indicates the correlation coefficient.

To analyze the transcriptomic profile of in vivo mouse early developmental stages and TBLCs, scRNA-seq datasets were downloaded from the Gene Expression Omnibus (GEO) website Available online:https://www.ncbi.nlm.nih.gov/geoprofiles/ (accessed on 12 July 2021) Through unsupervised clustering followed by UMAP dimensional reduction plotting, TBLCs and in vivo mouse early development cells were segregated into clusters and each cluster was labeled (Figure 3A,B). In vivo mouse early development clusters did not strongly overlap with TBLCs under the low-dimensional space (Figure 3A). To assess whether the mid-late two-cell stage expresses a totipotency marker, a violin plot was constructed to visualize the expression of *Zscan4s*. Only the mid-late two-cell clusters showed enriched *Zscan4a-d* gene expression, indicating that *Zscan4s* are robustly and transiently expressed in the mouse two-cell blastomere state following ZGA (Figure 3C). To visualize the levels of association between each identified cluster, the gene expression patterns of cells from each defined cluster were averaged, and a hierarchical clustering tree was constructed based on a distance matrix considering the global transcriptome. Strikingly, the hierarchical clustering analysis showed that TBLCs were more closely related to ESCs but distinct from other earlier mouse developmental stages such as early two-cell stage cells and zygotes (Figure 3D), indicating that TBLCs appeared to be similar to ESCs when visualized under the clustering tree. Principal component analysis (PCA) of the first 3 PCs revealed that TBLCs were distinct from in vivo mouse early developmental stages (Figure 3E). In contrast, TBLCs were tightly associated with ESCs under the first 3 PC dimensions. To assess the correlation between each identified cluster, the averaged gene expressions of each cell cluster were compared by correlation analyses at the global transcriptomic level. Consistent with hierarchical clustering analyses and PCA, TBLCs were more closely associated with ESCs than early cell stages (Figure 3F).

### 3.2. The Mid-Late Two-Cell Cluster Exhibits Greater Totipotency and Lower Pluripotency Gene Expression Patterns Compared to TBLCs

A previous study reported that the treatment of a single spliceosome inhibitor, pladienolide B (PlaB), was able to facilitate pluripotent mESCs reprogramming into a totipotent state [14]. To address the difference between TBLCs and early embryos, differential gene expression analyses were performed to analyze significantly upregulated and downregulated gene clusters in zygotes to early two-cell stages and in the mid-late two-cell stages as compared to TBLCs.

**Figure 4:**
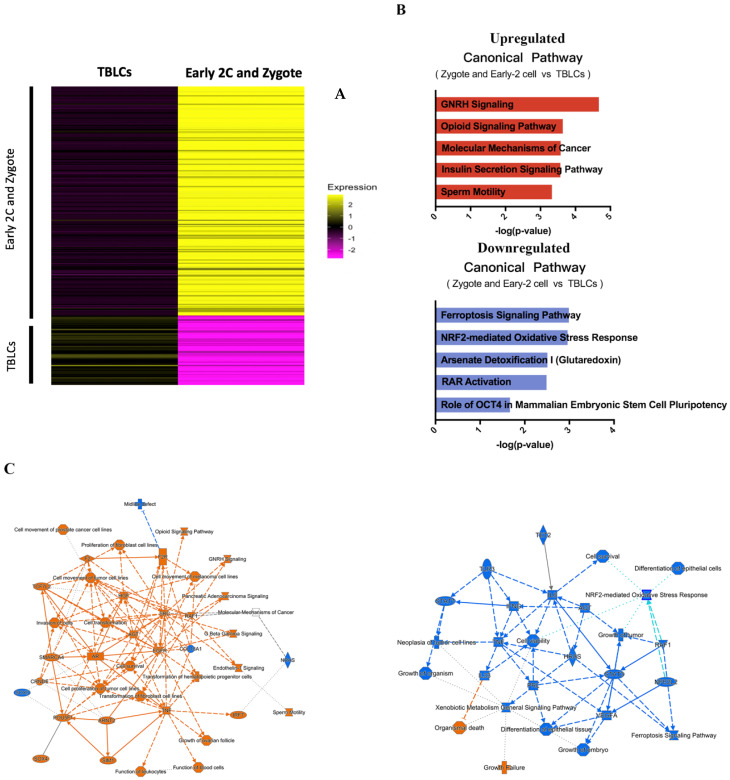
Differential gene and pathway analyses of TBLCs and zygote-early 2-cells. (**A**) Heatmap showing the average differential gene expression patterns of zygote-early 2-cells (top) and TBLCs (bottom). Scale bar indicates z-scored gene expression value. (**B**) Top 5 canonical pathways derived from ingenuity pathway analysis (IPA) gene ontology of gene markers of zygote-early 2-cells (top) and TBLCs (bottom). (**C**) Graphical summary of the predicted pathway regulation for zygote-early 2-cells (left) and TBLCs (right) gene markers. Orange lines indicate upregulation while blue lines indicate downregulation.

A heatmap showing the average gene expression of zygote-early two-cell clusters and TBLCs marker genes (upregulated) was derived from differential gene analyses where only genes with Bonferroni-corrected *p* < 0.05 and |log_2_(FoldChange)| ≥ 1 values were selected (Figure 4A). To investigate whether the identified DE genes are involved in cellular totipotency, we performed ingenuity pathway analyses (IPA) and found none of the essential pathways related to cellular totipotency were found in the zygote-early two-cells cluster-enriched genes compared to TBLCs (Figure 4B,C). This may be due to a limited understanding of the genes of the zygote and the early two-cell stage. By contrast, in the IPA analyses, the ferroptosis signaling pathway was found to be enriched in TBLCs (Figure 4B,C). One of the notably enriched genes in TBLCs was *Ctsb* (log_2_(FoldChange) = 2.77), which is a gene upregulated in the inner cell mass [15]. Thus, TBLCs may behave more like ESCs, which is a cell type derived from the inner cell mass.

**Figure 5:**
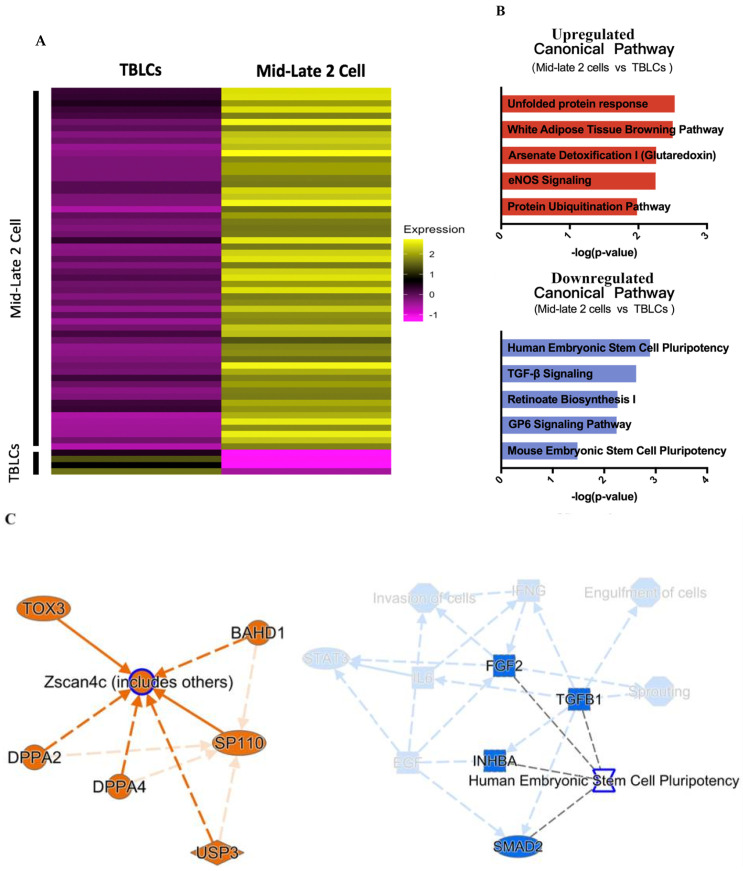
Differential gene and pathway analyses of TBLCs and mid-late 2-cells. (**A**) Heatmaps showing average differential gene expression patterns of mid-late 2-cells (top) and TBLCs (bottom) gene markers. Scale bar indicates z-scored gene expression value. (**B**) The top 5 canonical pathways were derived from ingenuity pathway analysis (IPA) gene ontology with gene markers of mid-late 2-cells (top) and TBLCs (bottom). (**C**) Graphical summary of the predicted pathway regulations of gene markers within mid-late 2-cells (left) and TBLCs (right). Orange lines indicate upregulation while blue colors indicate downregulation.

The next comparison group with TBLCs to be analyzed was the mid-late two-cell cluster which expresses a high level of totipotent marker genes like *Zscan4s*. Using the same filtration criteria for differential gene analyses (Bonferroni-corrected *p* < 0.5, |log_2_(FoldChange)| ≥ 1), a heatmap was constructed based on the differential genes expressed in mid-late two-cell clusters and TBLCs (Figure 5A). Comparing the mid-late two-cell cluster with TBLCs, many upregulated genes (including *Dppa2*, *Dppa4*, *Sp110*, *Usp3*, *Bahd1*, and *Tox3*) were notable because they are known to be involved in the *Zscan4s* signaling pathway (Figure 5B,C). This result suggested that totipotent genes were more enriched in mid-late two-cells but deprived in TBLCs. Next, we checked whether the TBLCs showed enriched pluripotent genes compared to the mid-late two-cells. The IPA analysis on the TBLCs-upregulated genes showed they are significantly related in pluripotency markers of human and mouse embryonic stem cells (Figure 5C). These genes include pluripotent signaling markers such as *Zfp42*, *Bmp4*, *Lefty1*, *Lefty2*, and *Nodal*. Since totipotent genes were downregulated and pluripotent genes were upregulated in TBLCs, it suggests that most of the TBLCs are still confined in a pluripotent state.

### 3.3. Cluster 3 of TBLCs Abundantly Expresses Totipotent Genes

TBLCs were reported to differentiate into embryonic and extraembryonic tissues in vivo [14]. However, the high similarity between TBLCs and ESCs made us hypothesize that there is a subpopulation responsible for this reported in vivo activity. The tight association between TBLCs and ESCs (Figure 3D–F) led us to further inspect the relationship between the two cell types in low-dimensional space (Figure S1A). Remarkably, the feature plots of ESCs and TBLCs showed that TBLCs contain a nonoverlapping subpopulation exhibiting enriched totipotency marker expression of *Zscan4c* and *Zscan4d* (Figure S1B). Thus, we next attempted to characterize the identity of this subpopulation.

**Figure 6:**
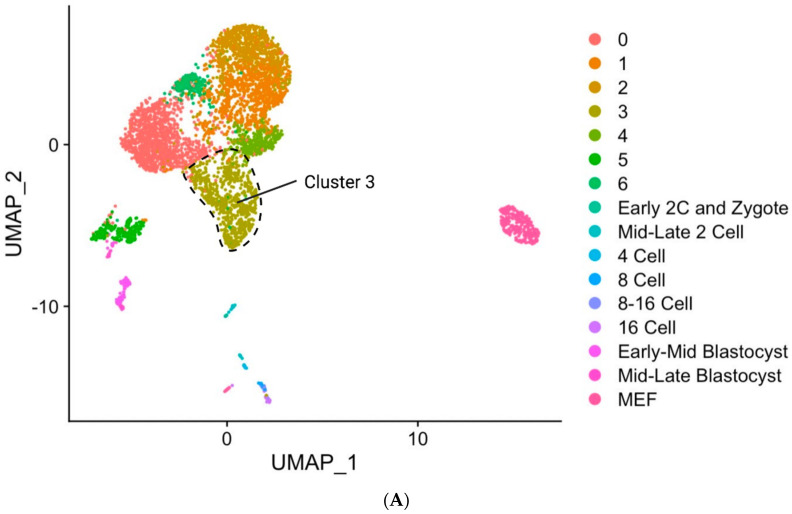

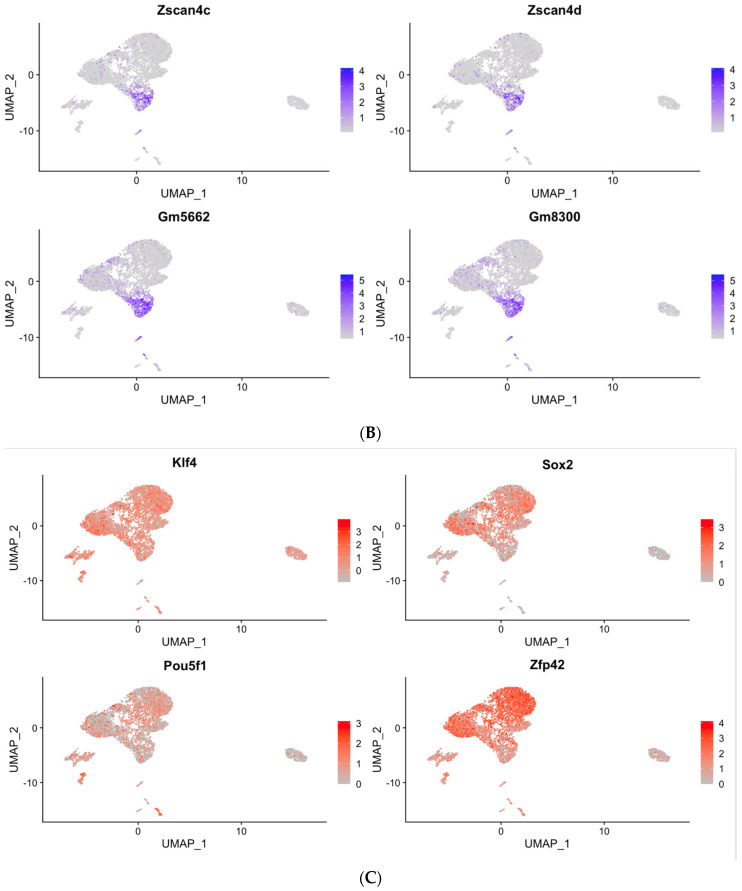
TBLCs clusters and the expression of totipotent and pluripotent gene markers. (**A**) UMAP dimensional reduction plot showing TBLCs clusters and early mouse embryonic developmental stages. (**B**) A feature plot revealing the totipotent marker gene expression on the UMAP dimensional reduction plot. Scale bar represents log-transformed gene expression. (**C**) Feature plot showing the pluripotent marker gene expression in TBLCs clusters on UMAP. Scale bar represents log-transformed gene expression.

**Figure 7:**
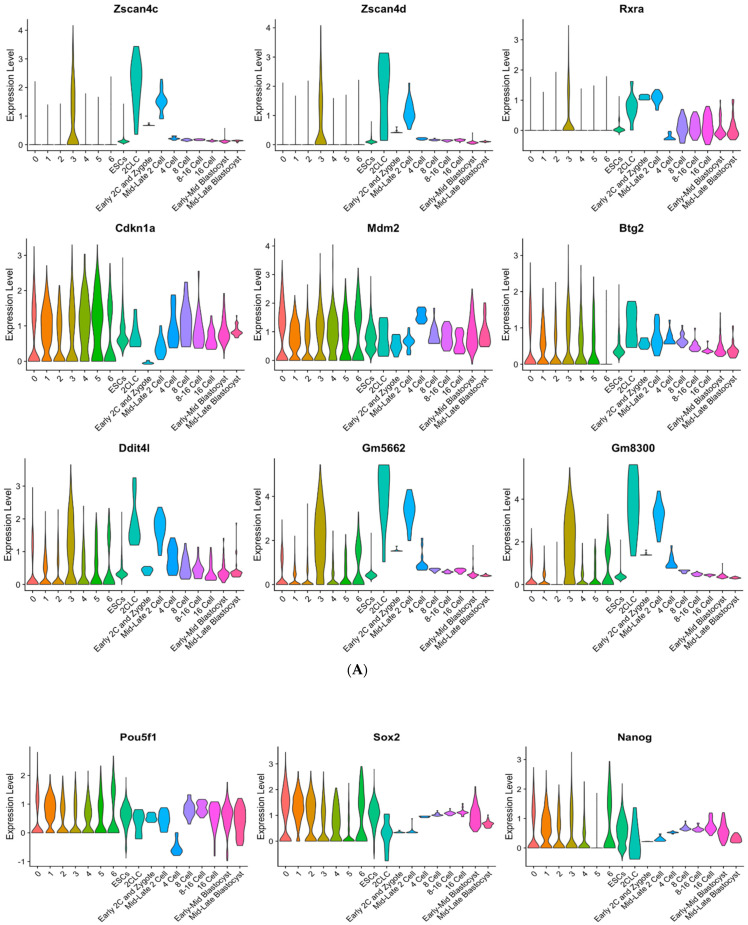

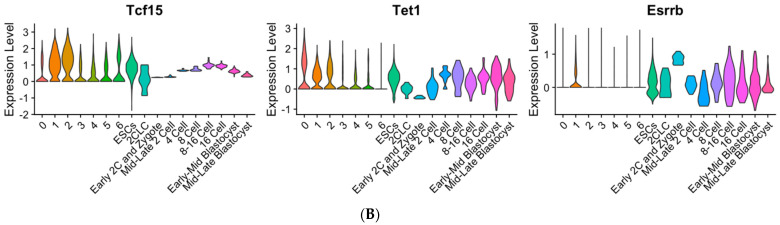
Totipotency gene and pluripotency gene expression of the TBLCs clusters, ESCs, early embryo cells, and 2CLCs. (**A**) Totipotent genes. (**B**) Pluripotent genes. *Y*-axis scale represents z-scored gene expression value.

TBLCs from the previous UMAP dimensional reduction plot (Figure 3B) were re-clustered at a higher resolution (Figure 6A). A feature plot was used to visualize the expression of totipotent gene markers including *Zscan4c*, *Zscan4d*, *Gm5662*, and *Gm8300*. As expected, one of the sorted clusters (cluster 3) showed greater totipotency marker gene expression as compared to all other clusters (Figure 6B). Pluripotent-specific gene markers such as *Klf4*, *Sox2*, *Pou5f1*, and *Zfp42* were all downregulated in cluster 3 as compared to other identified clusters of TBLCs (Figure 6C). Downregulation of pluripotent gene expression is a hallmark of cellular totipotency [7]. Cluster 3 is closely associated with zygote-early two-cell and mid-late two-cells under the UMAP plot (Figure 6A). Many totipotent genes are upregulated in the cluster 3, while pluripotent genes are downregulated in cluster 3 (Figure 7 and Figure S2). Violin plots revealed that *Zscan4c*, *Zscan4d*, *Rxra*, *CdKn1a*, *Mdm2*, *Btg2*, *Ddit4l*, *Gm5562*, and *Gm8300* expression were all upregulated in cluster 3 and mid-late two-cells (Figure 7A). Consistently, pluripotent genes *Pou5f1*, *Sox2*, *Nanog*, *Tcf15*, *Tet1,* and *Esrrb* were downregulated in the cluster 3 as compared to ESCs (Figure 7B). However, some of the differentially expressed genes detected in total TBLCs compared to ESCs (the previous paper [14] Figure S3H) did not have differences in cluster 3 (Figure S2), which may be caused by differential expression in other clusters.

### 3.4. Gene Expression Patterns of Cluster 3 in TBLCs Were Highly Correlated with Totipotent Mid-Late Two-Cell Stage Cells

**Figure 8:**
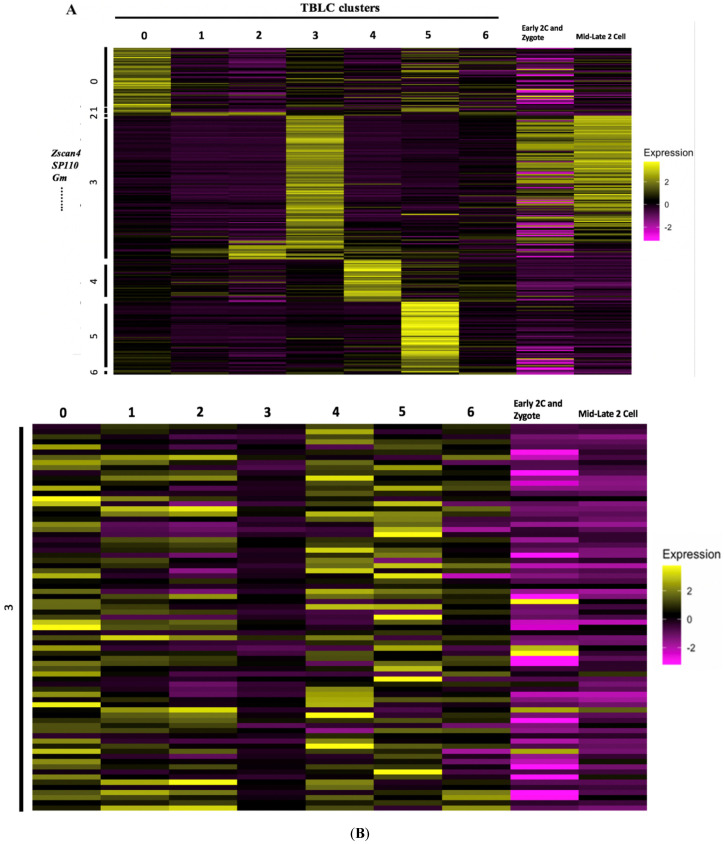

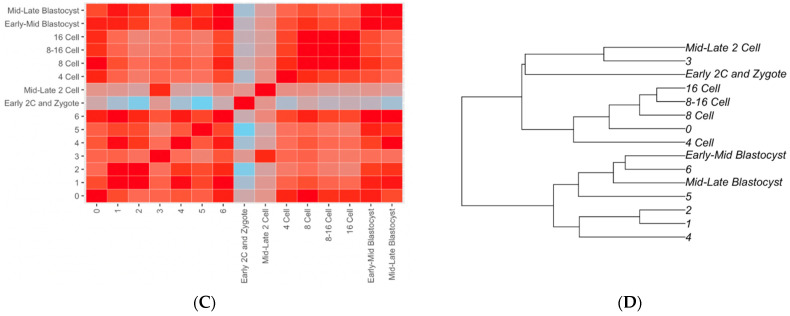
Transcriptomic analyses of TBLCs cluster 3 compared to other TBLCs clusters and in vivo mouse early developmental stages. (**A**) A heatmap showing average differential upregulated gene expression of TBLCs clusters, zygote-early 2-cells, and mid-late 2-cells. Scale bar represents z-scored gene expression value. (**B**) A heatmap showing average gene expression of TBLCs cluster 3 downregulated genes compared with other TBLCs clusters and 2-cell stage cells. Scale bar represents z-scored gene expression value. (**C**) A correlation matrix of TBLCs clusters and early mouse embryonic developmental stages based on first 20 PCs using Pearson’s correlation coefficient method. Scale bar indicates correlation coefficient. (**D**) A hierarchical clustering tree of TBLCs clusters and early mouse embryonic developmental stages based on first 20 PCs.

To further investigate the transcriptomic features in cluster 3, a heatmap was constructed to show the average gene expression of the various TBLCs clusters, mid-late two-cells, early two-cells, and zygotes based on each of the TBLCs cluster markers (Figure 8A). As expected, unlike other clusters of TBLCs, cluster 3 cells showed unique transcriptomic features such as greater totipotency-specific gene expression of *Zscan4*, *Sp110*, and the *Gm* protein family. Cluster 3 upregulated genes are similarly increased in mid-late two-cells (Figure 8A). Consistent with this observation, when cluster 3 downregulated gene markers derived from differential gene expression analyses were input into the data to construct heatmaps, mid-late two-cells showed similar downregulation patterns with cluster 3 (Figure 8B). To assess the correlation between the various clusters in TBLCs and different mouse embryo developmental stages, the average gene expression of each sample was compared by correlation analyses based on the first 20 PCs. Only cluster 3 in TBLCs showed a high correlation with mid-late two-cells (Figure 8C). Also, through hierarchical clustering analyses, only cluster 3 was closely related to mid-late two-cells, which is consistent with the correlation heatmap analyses (Figure 8D). These results revealed that only cluster 3 has a similar gene expression profile as the in vivo mid-late two-cells.

### 3.5. Unique Signaling Pathways and Regulation in Cluster 3 Cells

**Figure 9:**
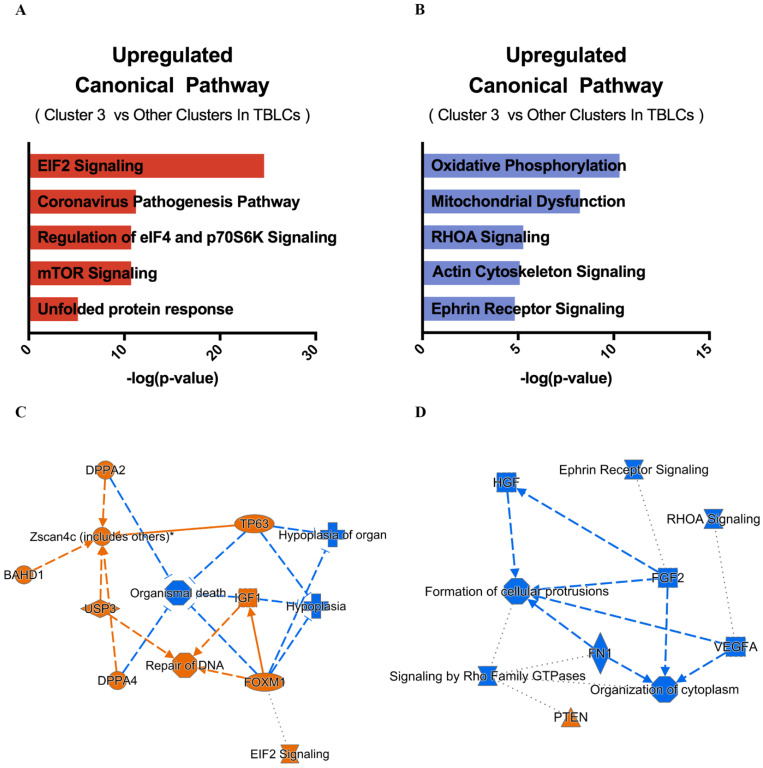
Gene ontology and pathway analyses of cluster 3 differentially expressed genes. (**A**) The top 5 canonical pathways were derived from the ingenuity pathway analysis (IPA) gene ontology of cluster 3 gene markers. (**B**) The top 5 canonical pathways were derived from the IPA gene ontology of cluster 3 downregulated genes. (**C**) Graphical summary of predicted pathway regulation of cluster 3 gene markers. (**D**) Graphical summary of predicted pathway regulation for cluster 3 downregulated genes. Orange lines indicate upregulation while blue lines indicate downregulation.

Cluster 3 cells showed unique transcriptomic features in TBLCs that are similar to totipotent blastomeres in vivo (Figure 6 and Figure 7). Therefore, we investigated different potential canonical signaling pathways uniquely manifested in cluster 3 using genes derived from differential gene analyses (Figure 8A,B). The IPA revealed that cluster 3 TBLCs were enriched with proliferation genes like the the EIF2 signaling pathway (Figure 9A). In combination with the analysis of the IPA, cluster 3 downregulated genes were ontologized to cellular energy consumption pathways, namely oxidative phosphorylation (Figure 9B).

Predicted pathway regulation analysis of cluster 3 also showed that many upregulated genes directly related to the *Zscan4c* expression pathway (Figure 9C). Totipotent and inhibiting hypoplasia gene candidates including *Zscan4c*, *Dppa2*, *Dppa4*, *Tp63*, *Igf1*, *Bahd1*, and *Usp3* are shown. On the other hand, the predicted pathway regulation analysis of cluster 3 downregulated genes showed cellular energy metabolism pathways including the RHOA and the Rho family GTPase. The critical cluster 3 downregulated genes *Fgf2* and *Vegfa* were previously reported to control mouse ESCs differentiation and direct ESCs to mesodermal and endothelial cell fate [16,17]. Thus, these findings suggest that cluster 3 in the TBLCs have unique totipotency features and pluripotency gene regulation which may govern the stem cell properties of the cells in this group.

## 4. Discussion

Our study reveals that cells of very early mouse embryonic developmental stages and TBLCs exhibit different transcriptomic features. Comparative transcriptomic analyses were performed using published data of TBLCs, mESCs, and preimplantation mouse embryos. The transcriptomic profile of TBLCs was close to ESCs but dissimilar to mouse mid-late two-cells according to our analyses (Figure 3, Figure 4 and Figure 5). This observation is contradictory to the previous study where they observed that TBLCs transcriptomes are close to 2- and 4-cell blastomeres [14]. This discrepancy may be caused by the differences in batch correction methods (canonical correlation analysis (CCA) using Seurat vs. the ‘Removebatcheffect’ function in the Limma R package). Conversely, it may simply be due to our analysis workflow using all single-cell sequencing data sets in contrast to others who combined the bulk sequence and single-cell sequence analyses for downstream analyses such as correlation analysis [14]. In addition, the limitations in single-cell sequencing in read depth compared to bulk sequencing could lead to these differences.

By comparing the zygote and early two-cells with TBLCs, based on IPA analyses, we observed many highly expressed genes in the early embryonic state before the major wave of zygote genome activation (ZGA). From gene ontology and canonical pathway analyses, TBLCs showed distinctive pathways and gene expression patterns as compared to zygote and early two-cells stage cells. A plausible explanation for this is that the major wave of ZGA has not occurred at the early two-cell stage cells, and thus this stage of embryonic development is dominated by maternal transcripts. Therefore, we cannot make any strong conclusion by comparing TBLCs and zygote-early two-cells by differential gene analysis and IPA. However, while comparing the expression profiles of TBLCs and the early two-cell stage, one of the most effective genes involved in downregulation in the ferroptosis signaling pathway is *Ctsb*. The *Ctsb* protein was reported to be enriched in the blastocyst and inner cell mass (ICM) [15,18]. However, in previous paper, Ctsb protein was suggested to be a two-cell-specific totipotency marker [14]. Other previous studies published that Ctsb protein plays an important role in implantation, pregnancy, and embryonic development [18]. The logical inference of which TBLCs express a high level of *Ctsb* is that TBLCs were closely related to mESCs from our hierarchical clustering analyses, PCA, and correlation analyses (Figure 3D–F). In contrast to the zygote and early two-cells, from the comparisons between the mid-late two-cells and TBLCs, TBLCs showed high expression levels of pluripotency genes and low expression of totipotency genes. In the predicted pathway regulation analyses, many of the differentiation-related genes are enriched in the TBLCs, including *Tgf1* and *Fgf2* [16,19].

TBLCs scRNA-seq datasets were reanalyzed by clustering TBLCs at a greater resolution in low-dimensional space. Remarkably, cluster 3 showed unique transcriptomic features as compared to other clusters (Figure 6, Figure 7, Figure 8 and Figure 9), with significantly higher totipotency gene expression markers such as *Zscan4c*, *Zscan4d*, *Sp110*, and *GM8300* (Figure 7A). In a previous study, the *Zscan4* gene has been thought to play important roles in embryo development [20], including DNA demethylation, maintenance of the telomere length, karyotype normality improvement, and quality. Cluster 3 showed lower pluripotency gene expression (*Pou5f1*, *Zfp42*, *Klf4*, and *Sox2*) compared with other TBLCs clusters (Figure 6C). The expression profiles of cluster 3 are also similar to the mid-late two-cell status according to differential gene analyses, correlation analyses, and hierarchical clustering analyses (Figure 7 and Figure 8). Consistently, under UMAP space, cluster 3 is tightly associated with mid-late two-cells (Figure 6A) In summary, we found that cluster 3 holds totipotency features and shows lower pluripotency gene expression as compared with the other TBLCs clusters.

The IPA analysis of cluster 3 upregulated genes showed the the Eif2 signaling pathway as the highest related canonical signaling pathway (Figure 9A). *Eif2* is a translational initiating factor [21], playing an important role in the regulation of mRNA translation. The EiF2 family was previously observed to maintain human and mouse ESC pluripotency [22]. In addition, *Eif2* maintains *Nanog* and *c-Myc* protein expression in mouse ESCs cultured without leukemia inhibitory factor (LIF) supplementation [22]. Also, *Eif2* is highly upregulated in the *Zscan4*-positive ESCs compared with the *Zscan4*-negative ESCs [23]. We infer that cluster 3 cells in TBLCs have more undifferentiated properties compared with other clusters. Other cluster 3 highly expressed genes shown in the predicted pathway regulation analyses can directly induce or activate *Zscan4c* (Figure 9C). These genes include *Dppa4*, which is highly related to the regulation of embryo expansion properties. Knocking down the *Dppa4* gene showed a reduction in the size of the mouse embryo; in addition, the lung tissues and the skeletal structures were malformed in the mouse model [24]. The *Igf1* gene is also highly expressed in cluster 3. The reduction in Igf1 activity was shown to result in ESC inferior proliferation and differentiation [25]. Furthermore, *Igf1* expression has a profound effect on size maintenance of embryonic and postnatal mice [26]. *Igf1* is an essential gene to promote mESC proliferation and antiapoptotic properties [27]. Notably, cluster 3 showed higher retinoid X receptors alpha (*Rxra*) gene expression (log_2_(FoldChange) = 1.41) (Figure 7A), which is a critical receptor that controls retinoic acid activation. Recent studies have showed that *Rxra* expression is upregulated in 2CLCs, and the high expression of retinoic acid can reprogram mESCs to the 2CLC state [28,29]. In the predicted pathway regulation analyses by the IPA, several low expression genes in cluster 3 revealed differentiation-related genes including *Fgf2* and *Vegfa*, where both are differentiation factors of ESCs [16,30]. These results indicated that cluster 3 has unique gene expression patterns that regulate undifferentiated, totipotent, and proliferative properties as compared to other clusters in the TBLCs.

**Figure 10:**
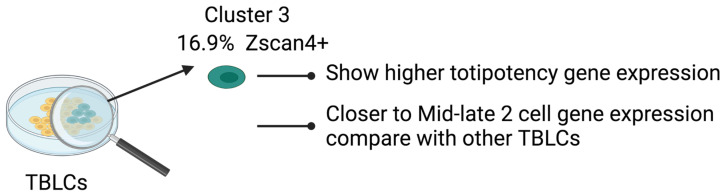
Summary image showing features of cluster 3 TBLCs.

Overall, our analysis showed that about 16.9% of cells in the TBLCs cluster 3 have higher expression of the totipotent genes and lower expression of pluripotent genes (Figure 10). We claim that cluster 3 resembles the previously reported 5% subpopulation in mESCs that are *Zscan4*-positive, but at a greater proportion (16.9%) [5,6]. Whether TBLCs cluster 3 can form chimerism and contribute to extraembryonic tissues remains unclear (Figure 10). In the previous studies, TBLCs were shown to maintain totipotency stably [14]. It would be of great interest to isolate cluster 3 cells in a culture system and perform chimeric assays. Since the totipotent population in mESCs cultures are difficult to maintain in 2CLCs [13], we speculate that the cluster 3 cells in TBLCs could have unique properties for the creation of sustainable in vitro maintenance, which may pave new experimental avenues in stem cell biology research and have many potential applications.

## Data Availability

Codes are provided in supplementary material.

## Supplementary Materials

The following are available online at https://www.mdpi.com/article/10.3390/cells10113111/s1. Figure S1: Single-cell RNA sequencing analyses of TBLCs and ESCs. Figure S2: Additional totipotency gene and pluripotency gene expression patterns of the TBLCs clusters, ESCs, early embryo cells, and 2CLCs.
