# Supplementary material for: Comparative Analyses of Single-Cell Transcriptomic Profiles between In Vitro Totipotent Blastomere-like Cells and In Vivo Early Mouse Embryonic Cells
Source: Cells. 2021 Nov 10;10(11):3111. doi: 10.3390/cells10113111 (PMC8621967; doi:10.3390/cells10113111)
Supplement: Supplementary file 1 [file cells-10-03111-s001.zip › Sup file/R code/2.0-Comparative-analysis-TBLC-vs-Early-Development.html]

 

 

 

 
 
 


 Comparative analysis of single-cell transcriptomic profiles between in vitro totipotent blastomere-like cells and in vivo early developments 

 
 
 
 
 
 
 
 
 
 
 
 

 

 
 


 


 

 

 


 

 


 


 


 Comparative analysis of single-cell transcriptomic profiles between in vitro totipotent blastomere-like cells and in vivo early developments 

 


 Loading TBLC (passage 6) data 
 Du et al.’s TBLC data “Mouse totipotent stem cells captured and maintained through spliceosomal repression” 
  # Load relevant packages
library(Seurat)  
  ## Attaching SeuratObject  
  library(dplyr)  
  ## 
## Attaching package: 'dplyr'  
  ## The following objects are masked from 'package:stats':
## 
##     filter, lag  
  ## The following objects are masked from 'package:base':
## 
##     intersect, setdiff, setequal, union  
  library(Matrix)
library(ggplot2)
library(cowplot)
library(patchwork)  
  ## 
## Attaching package: 'patchwork'  
  ## The following object is masked from 'package:cowplot':
## 
##     align_plots  
  library(writexl)  
  # Load TBLCs files
TBLCCountMatrix <- read.csv("~/Desktop/Internship/PlaB data/SCTBLCcount.csv")  
  #CreateSeuratObject
# filter the raw data: keep genes expressed in >=10 cells and keep cell with >200 genes i.e. filter genes
TBLC <- CreateSeuratObject(counts = TBLCCountMatrix, project = "TBLC")
TBLC  
  ## An object of class Seurat 
## 18184 features across 4534 samples within 1 assay 
## Active assay: RNA (18184 features, 0 variable features)  
  #QC control
# 1.number of unique genes detected in each cell
# 2.total number of molecules detected in each cell (strongly correlated with point 1)
# 3.the percentage of reads that map to the mitochondrial genome - PercentageFeature calculates the percentage of counts originating from a set of features
TBLC$"percent.mt" <- PercentageFeatureSet(TBLC, pattern = "^mt-")
# Visualize QC metrics as a violin plot
VlnPlot(TBLC, features = c("nFeature_RNA", "nCount_RNA", "percent.mt"), ncol = 3)  
   
  #find substring - i.e. mitochondrial genes
mito.genes <- grep(pattern = "^mt-", x = rownames(TBLC@assays[["RNA"]]), value = TRUE)
mito.genes  
  ##  [1] "mt-Nd1"  "mt-Nd2"  "mt-Co1"  "mt-Co2"  "mt-Atp8" "mt-Atp6" "mt-Co3" 
##  [8] "mt-Nd3"  "mt-Nd4l" "mt-Nd4"  "mt-Nd5"  "mt-Nd6"  "mt-Cytb"  
  head(TBLC@meta.data)  
 
 
 
  # FeatureScatter is typically used to visualize feature-feature relationships, but can be used
# for anything calculated by the object, i.e. columns in object metadata, PC scores etc.

TBLCplot1 <- FeatureScatter(TBLC, feature1 = "nCount_RNA", feature2 = "percent.mt")
TBLCplot2 <- FeatureScatter(TBLC, feature1 = "nCount_RNA", feature2 = "nFeature_RNA")
TBLCplot3 <- FeatureScatter(TBLC, feature1 = "nFeature_RNA", feature2 = "percent.mt")
TBLCplot1  
   
  TBLCplot2  
   
  TBLCplot3  
   
  #Normalizing Data: normalizes the feature expression measurements for each cell by the total expression (all genes a cell expresses), multiplies this by a scale factor (10,000 by default), and log-transforms the result. Normalized values are stored in TBLC[["RNA"]]@data.
#Normalized value stored here   TBLC$"RNA"@data
TBLC <- NormalizeData(TBLC, normalization.method = "LogNormalize", scale.factor = 10000)

#Identification of highly variable features (feature selection) - with 2000 variable features per dataset
TBLC <- FindVariableFeatures(TBLC, selection.method = "vst", nfeatures = 2000)
TBLC  
  ## An object of class Seurat 
## 18184 features across 4534 samples within 1 assay 
## Active assay: RNA (18184 features, 2000 variable features)  
  #Next, we apply a linear transformation (‘scaling’) that is a standard pre-processing step prior to dimensional reduction techniques like PCA. The ScaleData function:

#Shifts the expression of each gene, so that the mean expression across cells is 0
#Scales the expression of each gene, so that the variance across cells is 1
#This step gives equal weight in downstream analyses, so that highly-expressed genes do not dominate
#The results of this are stored in TBLC[["RNA"]]@scale.data

TBLC <- ScaleData(TBLC)  
  ## Centering and scaling data matrix  
  #Perform PCA on scaled data
TBLC <- RunPCA(TBLC, features = VariableFeatures(object = TBLC),verbose=FALSE)  
  #Clustering Cells (0.01)
TBLC <- FindNeighbors(TBLC, dims = 1:15)  
  ## Computing nearest neighbor graph  
  ## Computing SNN  
  TBLC <- FindClusters(TBLC, resolution = 0.01)  
  ## Modularity Optimizer version 1.3.0 by Ludo Waltman and Nees Jan van Eck
## 
## Number of nodes: 4534
## Number of edges: 152115
## 
## Running Louvain algorithm...
## Maximum modularity in 10 random starts: 0.9915
## Number of communities: 2
## Elapsed time: 0 seconds  
  # Run non-linear dimentional reduction (UMAP/tSNE)
# If you haven't installed UMAP, you can do so via reticulate::py_install(packages =
# 'umap-learn')
TBLC <- RunUMAP(TBLC, dims = 1:15)  
  ## Warning: The default method for RunUMAP has changed from calling Python UMAP via reticulate to the R-native UWOT using the cosine metric
## To use Python UMAP via reticulate, set umap.method to 'umap-learn' and metric to 'correlation'
## This message will be shown once per session  
  ## 15:39:09 UMAP embedding parameters a = 0.9922 b = 1.112  
  ## 15:39:09 Read 4534 rows and found 15 numeric columns  
  ## 15:39:09 Using Annoy for neighbor search, n_neighbors = 30  
  ## 15:39:09 Building Annoy index with metric = cosine, n_trees = 50  
  ## 0%   10   20   30   40   50   60   70   80   90   100%  
  ## [----|----|----|----|----|----|----|----|----|----|  
  ## **************************************************|
## 15:39:09 Writing NN index file to temp file /var/folders/cv/cfhg7t_j5y3d34y2_81gskv00000gn/T//RtmpdT6yOL/file4d3141a524e0
## 15:39:09 Searching Annoy index using 1 thread, search_k = 3000
## 15:39:10 Annoy recall = 100%
## 15:39:10 Commencing smooth kNN distance calibration using 1 thread
## 15:39:11 Initializing from normalized Laplacian + noise
## 15:39:11 Commencing optimization for 500 epochs, with 186234 positive edges
## 15:39:16 Optimization finished  
  # note that you can set `label = TRUE` or use the LabelClusters function to help label
# individual clusters
DimPlot(TBLC, reduction = "umap")  
   
  # Assigning cluster names for future reference and data integration
TBLC.cluster.ids <- c("TBLCs","MEF")
names(TBLC.cluster.ids) <- levels(TBLC)
TBLC <- RenameIdents(TBLC, TBLC.cluster.ids)
DimPlot(TBLC, reduction = "umap", label = TRUE, label.size=5) + NoLegend()  
   
  FeaturePlot(TBLC,features = "Zscan4c")  
   
 Loading early mouse embryo data 
 Daniel et al.’s early mouse embryo data “Single-cell RNA-Seq reveals dynamic, random monoallelic gene expression in mammalian cells” 
  # Load merged txt file sent by author (Deng et al.)
EarlyDev.data <- read.table("~/Desktop/Internship/Early Mouse Development data/expression_matrix.txt")  
  #CreateSeuratObject
# filter the raw data: keep genes expressed in >=10 cells and keep cell with >200 genes i.e. filter genes
EarlyDeve = CreateSeuratObject(counts = EarlyDev.data, project = "EarlyDevelopment", min.cells = 10, min.features = 200)  
  ## Warning: Feature names cannot have pipe characters ('|'), replacing with dashes
## ('-')  
  EarlyDeve  
  ## An object of class Seurat 
## 17176 features across 259 samples within 1 assay 
## Active assay: RNA (17176 features, 0 variable features)  
  #QC control
# 1.number of unique genes detected in each cell
# 2.total number of molecules detected in each cell (strongly correlated with point 1)
# 3.the percentage of reads that map to the mitochondrial genome - PercentageFeature calculates the percentage of counts originating from a set of features
EarlyDeve$"percent.mt" <- PercentageFeatureSet(EarlyDeve, pattern = "^Mt")

# Visualize QC metrics as a violin plot
VlnPlot(EarlyDeve, features = c("nFeature_RNA", "nCount_RNA", "percent.mt"), ncol = 3)  
   
  #find substring - i.e. mitochondrial genes
mito.genes <- grep(pattern = "^Mt", x = rownames(EarlyDeve@assays[["RNA"]]), value = TRUE)
mito.genes  
  ##  [1] "Mt1"     "Mt2"     "Mt4"     "Mta1"    "Mta2"    "Mta3"    "Mtap"   
##  [8] "Mtap1a"  "Mtap1b"  "Mtap1s"  "Mtap2"   "Mtap4"   "Mtap6"   "Mtap7"  
## [15] "Mtap7d1" "Mtap7d2" "Mtap7d3" "Mtap9"   "Mtbp"    "Mtch1"   "Mtch2"  
## [22] "Mtcp1"   "Mtdh"    "Mterf"   "Mterfd1" "Mterfd2" "Mterfd3" "Mtf1"   
## [29] "Mtf2"    "Mtfmt"   "Mtfp1"   "Mtfr1"   "Mtg1"    "Mthfd1"  "Mthfd1l"
## [36] "Mthfd2"  "Mthfd2l" "Mthfr"   "Mthfs"   "Mthfsd"  "Mtif2"   "Mtif3"  
## [43] "Mtl5"    "Mtm1"    "Mtmr1"   "Mtmr10"  "Mtmr11"  "Mtmr12"  "Mtmr14" 
## [50] "Mtmr2"   "Mtmr3"   "Mtmr4"   "Mtmr6"   "Mtmr7"   "Mtmr9"   "Mto1"   
## [57] "Mtor"    "Mtpap"   "Mtpn"    "Mtr"     "Mtrf1"   "Mtrf1l"  "Mtrnr2l"
## [64] "Mtrr"    "Mtss1"   "Mtss1l"  "Mttp"    "Mtus1"   "Mtus2"   "Mtx1"   
## [71] "Mtx2"    "Mtx3"  
  head(EarlyDeve@meta.data)  
 
 
 
  # FeatureScatter is typically used to visualize feature-feature relationships, but can be used
# for anything calculated by the object, i.e. columns in object metadata, PC scores etc.

Earlydeveplot1 <- FeatureScatter(EarlyDeve, feature1 = "nCount_RNA", feature2 = "percent.mt")
Earlydeveplot2 <- FeatureScatter(EarlyDeve, feature1 = "nCount_RNA", feature2 = "nFeature_RNA")
Earlydeveplot3 <- FeatureScatter(EarlyDeve, feature1 = "nFeature_RNA", feature2 = "percent.mt")
Earlydeveplot1   
   
  Earlydeveplot2  
   
  Earlydeveplot3  
   
  # filter samples
# We filter out cells that have unique feature counts over 2,500 or less than 200
# We filter out cells that have >5% mitochondrial counts

# Cells with high nCount_RNA would not be filtered out because they are late 2 cells with zygotic gene activation (ZGA) status. This means late 2 cells are an exception during filtering procedure as they theoretically have high nCount_RNA.
# No cells were filtered out in quality control since percentage mitochondrial counts are all less than 5 %.  
  #Normalizing Data: normalizes the feature expression measurements for each cell by the total expression (all genes a cell expresses), multiplies this by a scale factor (10,000 by default), and log-transforms the result. Normalized values are stored in PlaB[["RNA"]]@data.
#Normalized value stored here   PlaB$"RNA"@data
EarlyDeve <- NormalizeData(EarlyDeve, normalization.method = "LogNormalize", scale.factor = 10000)

#Identification of highly variable features (feature selection) - with 2000 variable features per dataset
EarlyDeve <- FindVariableFeatures(EarlyDeve, selection.method = "vst", nfeatures = 2000)
EarlyDeve  
  ## An object of class Seurat 
## 17176 features across 259 samples within 1 assay 
## Active assay: RNA (17176 features, 2000 variable features)  
  #Next, we apply a linear transformation (‘scaling’) that is a standard pre-processing step prior to dimensional reduction techniques like PCA. The ScaleData function:

#Shifts the expression of each gene, so that the mean expression across cells is 0
#Scales the expression of each gene, so that the variance across cells is 1
#This step gives equal weight in downstream analyses, so that highly-expressed genes do not dominate
#The results of this are stored in PlaB[["RNA"]]@scale.data

EarlyDeve <- ScaleData(EarlyDeve)  
  ## Centering and scaling data matrix  
  #Perform PCA on scaled data
EarlyDeve <- RunPCA(EarlyDeve, features = VariableFeatures(object = EarlyDeve),verbose=FALSE)  
  #Clustering Cells
EarlyDeve <- FindNeighbors(EarlyDeve, dims = 1:8)  
  ## Computing nearest neighbor graph  
  ## Computing SNN  
  EarlyDeve <- FindClusters(EarlyDeve, resolution = 3)  
  ## Modularity Optimizer version 1.3.0 by Ludo Waltman and Nees Jan van Eck
## 
## Number of nodes: 259
## Number of edges: 5866
## 
## Running Louvain algorithm...
## Maximum modularity in 10 random starts: 0.4452
## Number of communities: 10
## Elapsed time: 0 seconds  
  # Run non-linear dimentional reduction (UMAP/tSNE)
# If you haven't installed UMAP, you can do so via reticulate::py_install(packages =
# 'umap-learn')
EarlyDeve <- RunUMAP(EarlyDeve, dims = 1:8)  
  ## 15:39:24 UMAP embedding parameters a = 0.9922 b = 1.112  
  ## 15:39:24 Read 259 rows and found 8 numeric columns  
  ## 15:39:24 Using Annoy for neighbor search, n_neighbors = 30  
  ## 15:39:24 Building Annoy index with metric = cosine, n_trees = 50  
  ## 0%   10   20   30   40   50   60   70   80   90   100%  
  ## [----|----|----|----|----|----|----|----|----|----|  
  ## **************************************************|
## 15:39:24 Writing NN index file to temp file /var/folders/cv/cfhg7t_j5y3d34y2_81gskv00000gn/T//RtmpdT6yOL/file4d312c5fb3c2
## 15:39:24 Searching Annoy index using 1 thread, search_k = 3000
## 15:39:24 Annoy recall = 100%
## 15:39:24 Commencing smooth kNN distance calibration using 1 thread
## 15:39:24 Initializing from normalized Laplacian + noise
## 15:39:24 Commencing optimization for 500 epochs, with 7956 positive edges
## 15:39:25 Optimization finished  
  # note that you can set `label = TRUE` or use the LabelClusters function to help label
# individual clusters
DimPlot(EarlyDeve, reduction = "umap")  
   
  # Assigning cluster names for future reference and data integration
EarlyDeve.cluster.ids <- c("Early-Mid Blastocyst", "Mid-Late Blastocyst", "16 Cell","Early-Mid Blastocyst", "Early-Mid Blastocyst", "8-16 Cell", "Mid-Late 2 Cell","8 Cell","4 Cell","Early 2C and Zygote")
names(EarlyDeve.cluster.ids) <- levels(EarlyDeve)
EarlyDeve <- RenameIdents(EarlyDeve, EarlyDeve.cluster.ids)
DimPlot(EarlyDeve, reduction = "umap", label = TRUE) + NoLegend()  
   
  # assign levels
my_levels3 <- c('Early 2C and Zygote', 'Mid-Late 2 Cell', '4 Cell', '8 Cell', '8-16 Cell', '16 Cell', 'Early-Mid Blastocyst', 'Mid-Late Blastocyst')
levels(EarlyDeve) <- my_levels3
# Totipotent markers violin plot
VlnPlot(EarlyDeve, features = c("Zscan4a","Zscan4b"),pt.size=0)  
   
  VlnPlot(EarlyDeve, features = c("Zscan4c","Zscan4d"),pt.size=0)  
   
 Loading ESC data 
 Du et al.’s ESC data “Mouse totipotent stem cells captured and maintained through spliceosomal repression” 
  # load ESC data
ESCCountMatrix <- read.csv("~/Desktop/Internship/ESC data/SCESCcount.csv")  
  #CreateSeuratObject
# filter the raw data: keep genes expressed in >=10 cells and keep cell with >200 genes i.e. filter genes
ESC= CreateSeuratObject(counts = ESCCountMatrix, project = "ESC")
ESC  
  ## An object of class Seurat 
## 17558 features across 4139 samples within 1 assay 
## Active assay: RNA (17558 features, 0 variable features)  
  #QC control
# 1.number of unique genes detected in each cell
# 2.total number of molecules detected in each cell (strongly correlated with point 1)
# 3.the percentage of reads that map to the mitochondrial genome - PercentageFeature calculates the percentage of counts originating from a set of features
ESC$"percent.mt" <- PercentageFeatureSet(ESC, pattern = "^mt-")

# Visualize QC metrics as a violin plot
VlnPlot(ESC, features = c("nFeature_RNA", "nCount_RNA", "percent.mt"), ncol = 3)  
   
  #find substring - i.e. mitochondrial genes
mito.genes <- grep(pattern = "^mt-", x = rownames(ESC@assays[["RNA"]]), value = TRUE)
mito.genes  
  ##  [1] "mt-Nd1"  "mt-Nd2"  "mt-Co1"  "mt-Co2"  "mt-Atp8" "mt-Atp6" "mt-Co3" 
##  [8] "mt-Nd3"  "mt-Nd4l" "mt-Nd4"  "mt-Nd5"  "mt-Nd6"  "mt-Cytb"  
  head(ESC@meta.data)  
 
 
 
  # FeatureScatter is typically used to visualize feature-feature relationships, but can be used
# for anything calculated by the object, i.e. columns in object metadata, PC scores etc.

ESCplot1 <- FeatureScatter(ESC, feature1 = "nCount_RNA", feature2 = "percent.mt")
ESCplot2 <- FeatureScatter(ESC, feature1 = "nCount_RNA", feature2 = "nFeature_RNA")
ESCplot3 <- FeatureScatter(ESC, feature1 = "nFeature_RNA", feature2 = "percent.mt")
ESCplot1   
   
  ESCplot2  
   
  ESCplot3  
   
  #Normalizing Data: normalizes the feature expression measurements for each cell by the total expression (all genes a cell expresses), multiplies this by a scale factor (10,000 by default), and log-transforms the result. Normalized values are stored in ESC[["RNA"]]@data.
#Normalized value stored here   ESC$"RNA"@data
ESC <- NormalizeData(ESC, normalization.method = "LogNormalize", scale.factor = 10000)

#Identification of highly variable features (feature selection) - with 2000 variable features per dataset
ESC <- FindVariableFeatures(ESC, selection.method = "vst", nfeatures = 2000)

#Next, we apply a linear transformation (‘scaling’) that is a standard pre-processing step prior to dimensional reduction techniques like PCA. The ScaleData function:

#Shifts the expression of each gene, so that the mean expression across cells is 0
#Scales the expression of each gene, so that the variance across cells is 1
#This step gives equal weight in downstream analyses, so that highly-expressed genes do not dominate
#The results of this are stored in ESC[["RNA"]]@scale.data

ESC <- ScaleData(ESC)  
  ## Centering and scaling data matrix  
  #Perform PCA on scaled data
ESC <- RunPCA(ESC, features = VariableFeatures(object = ESC),nfeatures.print=2000,verbose = FALSE)  
  #Clustering Cells
ESC <- FindNeighbors(ESC, dims = 1:20)  
  ## Computing nearest neighbor graph  
  ## Computing SNN  
  ESC <- FindClusters(ESC, resolution = 0)  
  ## Modularity Optimizer version 1.3.0 by Ludo Waltman and Nees Jan van Eck
## 
## Number of nodes: 4139
## Number of edges: 135955
## 
## Running Louvain algorithm...
## Maximum modularity in 10 random starts: 1.0000
## Number of communities: 1
## Elapsed time: 0 seconds  
  # Run non-linear dimentional reduction (UMAP/tSNE)
# If you haven't installed UMAP, you can do so via reticulate::py_install(packages =
# 'umap-learn')
ESC <- RunUMAP(ESC, dims = 1:20)  
  ## 15:40:06 UMAP embedding parameters a = 0.9922 b = 1.112  
  ## 15:40:06 Read 4139 rows and found 20 numeric columns  
  ## 15:40:06 Using Annoy for neighbor search, n_neighbors = 30  
  ## 15:40:06 Building Annoy index with metric = cosine, n_trees = 50  
  ## 0%   10   20   30   40   50   60   70   80   90   100%  
  ## [----|----|----|----|----|----|----|----|----|----|  
  ## **************************************************|
## 15:40:06 Writing NN index file to temp file /var/folders/cv/cfhg7t_j5y3d34y2_81gskv00000gn/T//RtmpdT6yOL/file4d315759ee94
## 15:40:06 Searching Annoy index using 1 thread, search_k = 3000
## 15:40:07 Annoy recall = 100%
## 15:40:07 Commencing smooth kNN distance calibration using 1 thread
## 15:40:08 Initializing from normalized Laplacian + noise
## 15:40:08 Commencing optimization for 500 epochs, with 168118 positive edges
## 15:40:13 Optimization finished  
  DimPlot(ESC, reduction = "umap", label = TRUE)  
   
  # Assigning cluster names for future reference and data integration
ESC.cluster.ids <- "ESCs"
names(ESC.cluster.ids) <- levels(ESC)
ESC <- RenameIdents(ESC, ESC.cluster.ids)
DimPlot(ESC, reduction = "umap", label = TRUE) + NoLegend()  
   
 CCA integration: Early Deve and avg TBLC UMAP 
  # merging avg TBLC and in vivo mouse early development data
EarlyDeveTBLC<-merge(EarlyDeve,TBLC)
head(EarlyDeveTBLC@meta.data)  
 
 
 
  # splitting object identify for downstream cluster analysis
EarlyDeveTBLCs <- SplitObject(EarlyDeveTBLC, split.by = "orig.ident")
# integrating data by integrating 2 seurat objects as input
EarlyDeveTBLCs.anchors <- FindIntegrationAnchors(object.list = EarlyDeveTBLCs, reduction = "cca", dims = 1:20)  
  ## Computing 2000 integration features  
  ## No variable features found for object1 in the object.list. Running FindVariableFeatures ...  
  ## No variable features found for object2 in the object.list. Running FindVariableFeatures ...  
  ## Scaling features for provided objects  
  ## Finding all pairwise anchors  
  ## Running CCA  
  ## Merging objects  
  ## Finding neighborhoods  
  ## Finding anchors  
  ##  Found 1249 anchors  
  ## Filtering anchors  
  ##  Retained 220 anchors  
  EarlyDeveTBLCs.combined <- IntegrateData(anchorset = EarlyDeveTBLCs.anchors, dims = 1:20)  
  ## Merging dataset 1 into 2  
  ## Extracting anchors for merged samples  
  ## Finding integration vectors  
  ## Finding integration vector weights  
  ## Integrating data  
  # perform integrated analysis
DefaultAssay(EarlyDeveTBLCs.combined) <- "integrated"

# Run the standard workflow for visualization and clustering
EarlyDeveTBLCs.combined <- ScaleData(EarlyDeveTBLCs.combined, verbose = FALSE)
EarlyDeveTBLCs.combined <- RunPCA(EarlyDeveTBLCs.combined, npcs = 30, verbose = FALSE)
# UMAP and Clustering
EarlyDeveTBLCs.combined <- RunUMAP(EarlyDeveTBLCs.combined, reduction = "pca", dims = 1:20)  
  ## 15:40:38 UMAP embedding parameters a = 0.9922 b = 1.112  
  ## 15:40:38 Read 4793 rows and found 20 numeric columns  
  ## 15:40:38 Using Annoy for neighbor search, n_neighbors = 30  
  ## 15:40:38 Building Annoy index with metric = cosine, n_trees = 50  
  ## 0%   10   20   30   40   50   60   70   80   90   100%  
  ## [----|----|----|----|----|----|----|----|----|----|  
  ## **************************************************|
## 15:40:39 Writing NN index file to temp file /var/folders/cv/cfhg7t_j5y3d34y2_81gskv00000gn/T//RtmpdT6yOL/file4d31455376eb
## 15:40:39 Searching Annoy index using 1 thread, search_k = 3000
## 15:40:40 Annoy recall = 100%
## 15:40:40 Commencing smooth kNN distance calibration using 1 thread
## 15:40:41 Initializing from normalized Laplacian + noise
## 15:40:41 Commencing optimization for 500 epochs, with 197216 positive edges
## 15:40:46 Optimization finished  
  #EarlyDeveTBLCs.combined <- FindNeighbors(EarlyDeveTBLCs.combined, reduction = "pca", dims = 1:20)
#EarlyDeveTBLCs.combined <- FindClusters(EarlyDeveTBLCs.combined, resolution = 1)
#visualize UMAP
DimPlot(EarlyDeveTBLCs.combined, reduction = "umap", group.by = "orig.ident")  
   
  # assign levels and dimplot
clustered_levels <- c('TBLCs', 'Early 2C and Zygote', 'Mid-Late 2 Cell', '4 Cell', '8 Cell', '8-16 Cell', '16 Cell', 'Early-Mid Blastocyst', 'Mid-Late Blastocyst','MEF')
levels(EarlyDeveTBLCs.combined) <- clustered_levels
DimPlot(EarlyDeveTBLCs.combined, reduction = "umap",label=FALSE,repel=TRUE,cols = c('dark grey','red','orange','violet','green','blue','purple','Coral','#7CAE00','Sienna'))  
   
  FeaturePlot(EarlyDeveTBLCs.combined,feature="Zscan4c")  
   
 CCA integration: Early Deve and clustered TBLC UMAP 
  # cluster TBLC at a greater resolution
TBLC <- FindClusters(TBLC, resolution = 0.5)  
  ## Modularity Optimizer version 1.3.0 by Ludo Waltman and Nees Jan van Eck
## 
## Number of nodes: 4534
## Number of edges: 152115
## 
## Running Louvain algorithm...
## Maximum modularity in 10 random starts: 0.8532
## Number of communities: 8
## Elapsed time: 0 seconds  
  # Run non-linear dimentional reduction (UMAP/tSNE)
# If you haven't installed UMAP, you can do so via reticulate::py_install(packages =
# 'umap-learn')
TBLC <- RunUMAP(TBLC, dims = 1:15)  
  ## 15:40:48 UMAP embedding parameters a = 0.9922 b = 1.112  
  ## 15:40:48 Read 4534 rows and found 15 numeric columns  
  ## 15:40:48 Using Annoy for neighbor search, n_neighbors = 30  
  ## 15:40:48 Building Annoy index with metric = cosine, n_trees = 50  
  ## 0%   10   20   30   40   50   60   70   80   90   100%  
  ## [----|----|----|----|----|----|----|----|----|----|  
  ## **************************************************|
## 15:40:49 Writing NN index file to temp file /var/folders/cv/cfhg7t_j5y3d34y2_81gskv00000gn/T//RtmpdT6yOL/file4d313b37670d
## 15:40:49 Searching Annoy index using 1 thread, search_k = 3000
## 15:40:50 Annoy recall = 100%
## 15:40:50 Commencing smooth kNN distance calibration using 1 thread
## 15:40:51 Initializing from normalized Laplacian + noise
## 15:40:51 Commencing optimization for 500 epochs, with 186234 positive edges
## 15:40:56 Optimization finished  
  # Assigning cluster names for future reference and data integration
new.cluster.ids <- c("0","1","3","2","4","MEF","5","6")
names(new.cluster.ids) <- levels(TBLC)
TBLC <- RenameIdents(TBLC, new.cluster.ids)  
  DimPlot(TBLC, reduction = "umap")  
   
  FeaturePlot(TBLC,feature="Zscan4c")  
   
  # merging avg TBLC and in vivo mouse early development data
EarlyDeveTBLC<-merge(EarlyDeve,TBLC)
head(EarlyDeveTBLC@meta.data)  
 
 
 
  # splitting object identity for downstream cluster analysis
EarlyDeveTBLCs <- SplitObject(EarlyDeveTBLC, split.by = "orig.ident")
# integrating data by integrating 2 seurat objects as input
EarlyDeveTBLCs.anchors <- FindIntegrationAnchors(object.list = EarlyDeveTBLCs , dims = 1:20)  
  ## Computing 2000 integration features  
  ## No variable features found for object1 in the object.list. Running FindVariableFeatures ...  
  ## No variable features found for object2 in the object.list. Running FindVariableFeatures ...  
  ## Scaling features for provided objects  
  ## Finding all pairwise anchors  
  ## Running CCA  
  ## Merging objects  
  ## Finding neighborhoods  
  ## Finding anchors  
  ##  Found 1249 anchors  
  ## Filtering anchors  
  ##  Retained 220 anchors  
  EarlyDeveTBLCs.combined <- IntegrateData(anchorset = EarlyDeveTBLCs.anchors, dims = 1:20)  
  ## Merging dataset 1 into 2  
  ## Extracting anchors for merged samples  
  ## Finding integration vectors  
  ## Finding integration vector weights  
  ## Integrating data  
  # perform integrated analysis
DefaultAssay(EarlyDeveTBLCs.combined) <- "integrated"

# Run the standard workflow for visualization and clustering
EarlyDeveTBLCs.combined <- ScaleData(EarlyDeveTBLCs.combined, verbose = FALSE)
EarlyDeveTBLCs.combined <- RunPCA(EarlyDeveTBLCs.combined, npcs = 30, verbose = FALSE)
# UMAP and Clustering
EarlyDeveTBLCs.combined <- RunUMAP(EarlyDeveTBLCs.combined, reduction = "pca", dims = 1:20)  
  ## 15:41:22 UMAP embedding parameters a = 0.9922 b = 1.112  
  ## 15:41:22 Read 4793 rows and found 20 numeric columns  
  ## 15:41:22 Using Annoy for neighbor search, n_neighbors = 30  
  ## 15:41:22 Building Annoy index with metric = cosine, n_trees = 50  
  ## 0%   10   20   30   40   50   60   70   80   90   100%  
  ## [----|----|----|----|----|----|----|----|----|----|  
  ## **************************************************|
## 15:41:22 Writing NN index file to temp file /var/folders/cv/cfhg7t_j5y3d34y2_81gskv00000gn/T//RtmpdT6yOL/file4d3179d60e73
## 15:41:22 Searching Annoy index using 1 thread, search_k = 3000
## 15:41:23 Annoy recall = 100%
## 15:41:23 Commencing smooth kNN distance calibration using 1 thread
## 15:41:24 Initializing from normalized Laplacian + noise
## 15:41:24 Commencing optimization for 500 epochs, with 197216 positive edges
## 15:41:30 Optimization finished  
  #EarlyDeveTBLCs.combined <- FindNeighbors(EarlyDeveTBLCs.combined, reduction = "pca", dims = 1:20)
#EarlyDeveTBLCs.combined <- FindClusters(EarlyDeveTBLCs.combined, resolution = 1)
#visualize UMAP
DimPlot(EarlyDeveTBLCs.combined, reduction = "umap", group.by = "orig.ident")  
   
  # assign levels and dimplot
clustered_levels <- c('0','1','2','3','4','5','6', 'Early 2C and Zygote', 'Mid-Late 2 Cell', '4 Cell', '8 Cell', '8-16 Cell', '16 Cell', 'Early-Mid Blastocyst', 'Mid-Late Blastocyst','MEF')
levels(EarlyDeveTBLCs.combined) <- clustered_levels

DimPlot(EarlyDeveTBLCs.combined, reduction = "umap",label=FALSE,repel=TRUE)  
   
  # totipotent markers: no anchor TBLC vs Earlydeve
FeaturePlot(EarlyDeveTBLCs.combined,features = c("Zscan4c","Zscan4d","Gm5662","Gm8300"), min.cutoff = "q8")  
   
  # pluripotent markers: no anchor TBLC vs Earlydeve
FeaturePlot(EarlyDeveTBLCs.combined,features = c("Klf4","Sox2","Pou5f1","Zfp42"),cols = c("grey", "red"))  
  ## Warning: Could not find Sox2 in the default search locations, found in RNA assay
## instead  
  ## Warning: Could not find Pou5f1 in the default search locations, found in RNA
## assay instead  
   
 Differential gene analysis: Integration (CCA) with Early mouse embryo and average TBLC 
  # cluster TBLC at a greater resolution
TBLC <- FindClusters(TBLC, resolution = 0.01)  
  ## Modularity Optimizer version 1.3.0 by Ludo Waltman and Nees Jan van Eck
## 
## Number of nodes: 4534
## Number of edges: 152115
## 
## Running Louvain algorithm...
## Maximum modularity in 10 random starts: 0.9915
## Number of communities: 2
## Elapsed time: 0 seconds  
  # Run non-linear dimentional reduction (UMAP/tSNE)
# If you haven't installed UMAP, you can do so via reticulate::py_install(packages =
# 'umap-learn')
TBLC <- RunUMAP(TBLC, dims = 1:15)  
  ## 15:41:34 UMAP embedding parameters a = 0.9922 b = 1.112  
  ## 15:41:34 Read 4534 rows and found 15 numeric columns  
  ## 15:41:34 Using Annoy for neighbor search, n_neighbors = 30  
  ## 15:41:34 Building Annoy index with metric = cosine, n_trees = 50  
  ## 0%   10   20   30   40   50   60   70   80   90   100%  
  ## [----|----|----|----|----|----|----|----|----|----|  
  ## **************************************************|
## 15:41:35 Writing NN index file to temp file /var/folders/cv/cfhg7t_j5y3d34y2_81gskv00000gn/T//RtmpdT6yOL/file4d312fc15af
## 15:41:35 Searching Annoy index using 1 thread, search_k = 3000
## 15:41:36 Annoy recall = 100%
## 15:41:36 Commencing smooth kNN distance calibration using 1 thread
## 15:41:37 Initializing from normalized Laplacian + noise
## 15:41:37 Commencing optimization for 500 epochs, with 186234 positive edges
## 15:41:42 Optimization finished  
  # Assigning cluster names for future reference and data integration
new.cluster.ids <- c("TBLCs","MEF")
names(new.cluster.ids) <- levels(TBLC)
TBLC <- RenameIdents(TBLC, new.cluster.ids)  
  # merging TBLC and in vivo mouse early development data
MPE1<-merge(EarlyDeve,TBLC)
head(MPE1@meta.data)  
 
 
 
  # splitting object identify for downstream cluster analysis
MPES1 <- SplitObject(MPE1, split.by = "orig.ident")
MPES1  
  ## $EarlyDevelopment
## An object of class Seurat 
## 21985 features across 259 samples within 1 assay 
## Active assay: RNA (21985 features, 0 variable features)
## 
## $TBLC
## An object of class Seurat 
## 21985 features across 4534 samples within 1 assay 
## Active assay: RNA (21985 features, 0 variable features)  
  # normalize and identify variable features for each dataset independently
MPES1 <- lapply(X = MPES1, FUN = function(x) {
    x <- NormalizeData(x)
    x <- FindVariableFeatures(x, selection.method = "vst", nfeatures = 2000)
})
# select features that are repeatedly variable across datasets for integration
features <- SelectIntegrationFeatures(object.list = MPES1)

# integrating data by integrating 2 seurat objects as input
MPES1.anchors <- FindIntegrationAnchors(object.list = MPES1, reduction = "cca", anchor.features = features,dim=1:20)  
  ## Scaling features for provided objects  
  ## Finding all pairwise anchors  
  ## Running CCA  
  ## Merging objects  
  ## Finding neighborhoods  
  ## Finding anchors  
  ##  Found 1249 anchors  
  ## Filtering anchors  
  ##  Retained 220 anchors  
  MPES1.combined <- IntegrateData(anchorset = MPES1.anchors,dim=1:20)  
  ## Merging dataset 1 into 2  
  ## Extracting anchors for merged samples  
  ## Finding integration vectors  
  ## Finding integration vector weights  
  ## Integrating data  
  # assign levels 
avgMPES1_levels <- c("TBLCs", 'Early 2C and Zygote', 'Mid-Late 2 Cell', '4 Cell', '8 Cell', '8-16 Cell', '16 Cell', 'Early-Mid Blastocyst', 'Mid-Late Blastocyst',"MEF")
levels(MPES1.combined) <- avgMPES1_levels
# average expressions
MPES1.combinedavgexp <- AverageExpression(MPES1.combined, return.seurat = T)  
  ## Warning: The following arguments are not used: row.names  
  ## Centering and scaling data matrix  
  # find Zygote and Early 2C vs avg TBLC markers
Early2CandZyvsvsavgTBLC.markers <- FindMarkers(MPES1.combined, ident.1 = "Early 2C and Zygote", ident.2 = "TBLCs", min.pct = 0.25, logfc.threshold = 0.25)
Early2CandZyvsvsavgTBLC.markers  
 
 
 
  AdjEarly2CandZyvsvsavgTBLC.markers <-subset(Early2CandZyvsvsavgTBLC.markers, p_val_adj < 0.05)

# convert to dataframe and give column name
as.data.frame(AdjEarly2CandZyvsvsavgTBLC.markers)  
 
 
 
  colnames(AdjEarly2CandZyvsvsavgTBLC.markers)[0] <- "Gene_Symbol"
AdjEarly2CandZyvsvsavgTBLC.markers<-tibble::rownames_to_column(AdjEarly2CandZyvsvsavgTBLC.markers, "Gene_Symbol") 
AdjEarly2CandZyvsvsavgTBLC.markers  
 
 
 
  # sorting differential expression by fold change order
LowerAdjEarly2CandZyvsvsavgTBLC.markers<-AdjEarly2CandZyvsvsavgTBLC.markers[order(AdjEarly2CandZyvsvsavgTBLC.markers$avg_log2FC),]
HigherAdjEarly2CandZyvsvsavgTBLC.markers<-AdjEarly2CandZyvsvsavgTBLC.markers[order(-AdjEarly2CandZyvsvsavgTBLC.markers$avg_log2FC),]
write_xlsx(HigherAdjEarly2CandZyvsvsavgTBLC.markers,"~/Desktop/HigherAdjEarly2CandZyvsvsavgTBLC.markers.xlsx")
write_xlsx(LowerAdjEarly2CandZyvsvsavgTBLC.markers,"~/Desktop/LowerAdjEarly2CandZyvsvsavgTBLC.markers.xlsx")  
  # Heatmap of Zygote and Early 2C vs avg TBLC markers
DoHeatmap(MPES1.combinedavgexp, features = HigherAdjEarly2CandZyvsvsavgTBLC.markers$Gene_Symbol[c(1:218,619:684)],hjust=0.5,angle=0, size=5,group.bar.height = 0.01, draw.lines = FALSE,cells=c("TBLCs",'Early 2C and Zygote'), disp.max = 1000, disp.min = -1000, )  
  ## Warning: Removed 8 rows containing missing values (geom_text).  
   
  # find Mid-Late 2C vs avg TBLC marker
MidLate2CvsavgTBLC.markers <- FindMarkers(MPES1.combined, ident.1 = "Mid-Late 2 Cell", ident.2 = "TBLCs", min.pct = 0.25, logfc.threshold = 0.25)
MidLate2CvsavgTBLC.markers  
 
 
 
  AdjMidLate2CvsavgTBLC.markers <-subset(MidLate2CvsavgTBLC.markers, p_val_adj < 0.05)

# convert to dataframe and give column name
as.data.frame(AdjMidLate2CvsavgTBLC.markers)  
 
 
 
  colnames(AdjMidLate2CvsavgTBLC.markers)[0] <- "Gene_Symbol"
AdjMidLate2CvsavgTBLC.markers<-tibble::rownames_to_column(AdjMidLate2CvsavgTBLC.markers, "Gene_Symbol") 
AdjMidLate2CvsavgTBLC.markers  
 
 
 
  # sorting differential expression by fold change order
LowerAdjMidLate2CvsavgTBLC.markers<-AdjMidLate2CvsavgTBLC.markers[order(AdjMidLate2CvsavgTBLC.markers$avg_log2FC),]
HigherAdjMidLate2CvsavgTBLC.markers<-AdjMidLate2CvsavgTBLC.markers[order(-AdjMidLate2CvsavgTBLC.markers$avg_log2FC),]
library("writexl")
write_xlsx(HigherAdjMidLate2CvsavgTBLC.markers,"~/Desktop/HigherAdjMidLate2CvsavgTBLC.markers.xlsx")
write_xlsx(LowerAdjMidLate2CvsavgTBLC.markers,"~/Desktop/LowerAdjMidLate2CvsavgTBLC.markers.xlsx")  
  # heat map 
DoHeatmap(MPES1.combinedavgexp, features = HigherAdjMidLate2CvsavgTBLC.markers$Gene_Symbol[c(1:58,467:470)],hjust=0.5,angle=0, size=5,group.bar.height = 0.01, draw.lines = FALSE,cells=c("TBLCs",'Mid-Late 2 Cell'), disp.max = 1000, disp.min = -1000)  
  ## Warning: Removed 8 rows containing missing values (geom_text).  
   
 Integration (CCA) with Early mouse embryo and average TBLC and ESC 
  # merging TBLC and in vivo mouse early development data
TBLCESC<-merge(TBLC,ESC)  
  ## Warning in CheckDuplicateCellNames(object.list = objects): Some cell names are
## duplicated across objects provided. Renaming to enforce unique cell names.  
  TBLCESCEarlyDeve<-merge(TBLCESC,EarlyDeve)
head(TBLCESCEarlyDeve@meta.data)  
 
 
 
  # splitting object identify for downstream cluster analysis
TBLCESCEarlyDeve1 <- SplitObject(TBLCESCEarlyDeve, split.by = "orig.ident")
TBLCESCEarlyDeve1  
  ## $TBLC
## An object of class Seurat 
## 22603 features across 4534 samples within 1 assay 
## Active assay: RNA (22603 features, 0 variable features)
## 
## $ESC
## An object of class Seurat 
## 22603 features across 4139 samples within 1 assay 
## Active assay: RNA (22603 features, 0 variable features)
## 
## $EarlyDevelopment
## An object of class Seurat 
## 22603 features across 259 samples within 1 assay 
## Active assay: RNA (22603 features, 0 variable features)  
  # normalize and identify variable features for each dataset independently
TBLCESCEarlyDeve1 <- lapply(X = TBLCESCEarlyDeve1, FUN = function(x) {
    x <- NormalizeData(x)
    x <- FindVariableFeatures(x, selection.method = "vst", nfeatures = 2000)
})

# select features that are repeatedly variable across datasets for integration
features <- SelectIntegrationFeatures(object.list = TBLCESCEarlyDeve1)
# integrating data by integrating 2 seurat objects as input
TBLCESCEarlyDeve1.anchors <- FindIntegrationAnchors(object.list = TBLCESCEarlyDeve1, reduction = "cca", anchor.features = features, dim=1:20)  
  ## Scaling features for provided objects  
  ## Finding all pairwise anchors  
  ## Running CCA  
  ## Merging objects  
  ## Finding neighborhoods  
  ## Finding anchors  
  ##  Found 12297 anchors  
  ## Filtering anchors  
  ##  Retained 3968 anchors  
  ## Running CCA  
  ## Merging objects  
  ## Finding neighborhoods  
  ## Finding anchors  
  ##  Found 1242 anchors  
  ## Filtering anchors  
  ##  Retained 1060 anchors  
  ## Running CCA  
  ## Merging objects  
  ## Finding neighborhoods  
  ## Finding anchors  
  ##  Found 1219 anchors  
  ## Filtering anchors  
  ##  Retained 1029 anchors  
  TBLCESCEarlyDeve1.combined <- IntegrateData(anchorset = TBLCESCEarlyDeve1.anchors,dim=1:20)  
  ## Merging dataset 3 into 1  
  ## Extracting anchors for merged samples  
  ## Finding integration vectors  
  ## Finding integration vector weights  
  ## Integrating data  
  ## Merging dataset 2 into 1 3  
  ## Extracting anchors for merged samples  
  ## Finding integration vectors  
  ## Finding integration vector weights  
  ## Integrating data  
  # hierarchical clustering tree of TBLC clusters vs early mouse embryo vs ESC
TBLCESCEarlyDeve1.combined1<-subset(x = TBLCESCEarlyDeve1.combined, idents = c('ESCs',"TBLCs", 'Early 2C and Zygote', 'Mid-Late 2 Cell', '4 Cell', '8 Cell', '8-16 Cell', '16 Cell', 'Early-Mid Blastocyst', 'Mid-Late Blastocyst'))

tree1<-BuildClusterTree(TBLCESCEarlyDeve1.combined1, assay="RNA")
tree1  
  ## An object of class Seurat 
## 24603 features across 8651 samples within 2 assays 
## Active assay: integrated (2000 features, 2000 variable features)
##  1 other assay present: RNA  
  PlotClusterTree(tree1,direction="rightwards")   
   
  # pull the tree
tree1 <- Tool(object = tree1, slot = "BuildClusterTree")
# plot the tree
ape::plot.phylo(x = tree1, direction = "rightwards")  
   
  # Correlation heatmap of TBLC clusters vs early mouse embryo vs ESC
# MEF is removed as it is not a cell type of interest
av.exp1 <- AverageExpression(TBLCESCEarlyDeve1.combined1)$RNA
cor.exp <- as.data.frame(cor(av.exp1))
cor.exp$x <- rownames(cor.exp)
cor.df1 <- tidyr::gather(data = cor.exp, y, correlation,  c('ESCs',"TBLCs", 'Early 2C and Zygote', 'Mid-Late 2 Cell', '4 Cell', '8 Cell', '8-16 Cell', '16 Cell', 'Early-Mid Blastocyst', 'Mid-Late Blastocyst'))

corClusMPES1_levels <- c('ESCs',"TBLCs", 'Early 2C and Zygote', 'Mid-Late 2 Cell', '4 Cell', '8 Cell', '8-16 Cell', '16 Cell', 'Early-Mid Blastocyst', 'Mid-Late Blastocyst')

ggplot(cor.df1, aes(x= factor(x, level = corClusMPES1_levels ), y=factor(y, level = corClusMPES1_levels ), fill = correlation)) + geom_tile(color='white')+scale_fill_gradient(low="skyblue", high="red")+ theme(axis.text.x = element_text(angle = 90, vjust = 0.5, hjust=1))    
   
 
 Run PCA code are shown below but will not be ran as it can not be viewed under html file 
 load rgl library(rgl) 
 create dataframe using average expression PCAav.exps <- AverageExpression(TBLCESCEarlyDeve1.combined)$integrated PCAav.exps.dataframe<-as.data.frame(PCAav.exps) PCAav.exps.dataframes<-PCAav.exps.dataframe[c(‘TBLCs’,‘ESCs’, ‘Early 2C and Zygote’, ‘Mid-Late 2 Cell’, ‘4 Cell’, ‘8 Cell’, ‘8-16 Cell’, ‘16 Cell’, ‘Early-Mid Blastocyst’, ‘Mid-Late Blastocyst’)] 
 PCA analysis PCAAverage<-prcomp(PCAav.exps.dataframes, scale. = TRUE) PCAAverage1<-get_pca_var(PCAAverage) 
 fit <- hclust(dist(PCAAverage1$coord[,1:3]), method=“complete”) # 1:3 -> based on 3 components groups <- cutree(fit, k=5) # k=5 -> 5 groups 
 plotting PCA but will not show here plotPCA <- function(x, nGroup) { n <- ncol(x) if(!(n %in% c(2,3))) { # check if 2d or 3d stop(“x must have either 2 or 3 columns”) } 
  fit <- hclust(dist(x), method="complete") # cluster
groups <- cutree(fit, k=nGroup)

if(n == 3) { # 3d plot
    plot3d(x, col=groups, type="s", size=1, axes=T, xlab="PCA 1", ylab="PCA 2", zlab="PCA 3")
    axes3d(edges=c("x--", "y--", "z--"), lwd=3, axes.len=2, labels=FALSE)
    grid3d("x")
    grid3d("y")
    grid3d("z")
    text3d(x, texts=name,adj=c(-0.25,1))
} else { # 2d plot
    maxes <- apply(abs(x), 2, max)
    rangeX <- c(-maxes[1], maxes[1])
    rangeY <- c(-maxes[2], maxes[2])
    plot(x, col=groups, pch=19, xlab=colnames(x)[1], ylab=colnames(x)[2], xlim=rangeX, ylim=rangeY)
    lines(c(0,0), rangeX*2)
    lines(rangeY*2, c(0,0))
}  
 } 
 Graphing PCA plotPCA(PCAAverage1 \(coord[,c(1,3)], 10) PCAAverage1\) coor 
 Differential gene analysis: Integration (CCA) with Early mouse embryo and clustered TBLC 
  # cluster TBLC at a greater resolution
TBLC <- FindClusters(TBLC, resolution = 0.5)  
  ## Modularity Optimizer version 1.3.0 by Ludo Waltman and Nees Jan van Eck
## 
## Number of nodes: 4534
## Number of edges: 152115
## 
## Running Louvain algorithm...
## Maximum modularity in 10 random starts: 0.8532
## Number of communities: 8
## Elapsed time: 0 seconds  
  # Run non-linear dimentional reduction (UMAP/tSNE)
# If you haven't installed UMAP, you can do so via reticulate::py_install(packages =
# 'umap-learn')
TBLC <- RunUMAP(TBLC, dims = 1:15)  
  ## 15:46:01 UMAP embedding parameters a = 0.9922 b = 1.112  
  ## 15:46:01 Read 4534 rows and found 15 numeric columns  
  ## 15:46:01 Using Annoy for neighbor search, n_neighbors = 30  
  ## 15:46:01 Building Annoy index with metric = cosine, n_trees = 50  
  ## 0%   10   20   30   40   50   60   70   80   90   100%  
  ## [----|----|----|----|----|----|----|----|----|----|  
  ## **************************************************|
## 15:46:01 Writing NN index file to temp file /var/folders/cv/cfhg7t_j5y3d34y2_81gskv00000gn/T//RtmpdT6yOL/file4d3153dc4af7
## 15:46:01 Searching Annoy index using 1 thread, search_k = 3000
## 15:46:02 Annoy recall = 100%
## 15:46:03 Commencing smooth kNN distance calibration using 1 thread
## 15:46:03 Initializing from normalized Laplacian + noise
## 15:46:03 Commencing optimization for 500 epochs, with 186234 positive edges
## 15:46:09 Optimization finished  
  # Assigning cluster names for future reference and data integration
TBLCs.clustered.ids <- c("0","1","3","2","4","MEF","5","6")
names(TBLCs.clustered.ids) <- levels(TBLC)
TBLC <- RenameIdents(TBLC, TBLCs.clustered.ids)  
  # merging TBLC and in vivo mouse early development data
MPE2<-merge(EarlyDeve,TBLC)
head(MPE2@meta.data)  
 
 
 
  # splitting object identity for downstream cluster analysis
MPES2 <- SplitObject(MPE2, split.by = "orig.ident")
MPES2  
  ## $EarlyDevelopment
## An object of class Seurat 
## 21985 features across 259 samples within 1 assay 
## Active assay: RNA (21985 features, 0 variable features)
## 
## $TBLC
## An object of class Seurat 
## 21985 features across 4534 samples within 1 assay 
## Active assay: RNA (21985 features, 0 variable features)  
  # normalize and identify variable features for each dataset independently
MPES2 <- lapply(X = MPES2, FUN = function(x) {
    x <- NormalizeData(x)
    x <- FindVariableFeatures(x, selection.method = "vst", nfeatures = 2000)
})
# select features that are repeatedly variable across datasets for integration
features <- SelectIntegrationFeatures(object.list = MPES2)

# integrating data by integrating 2 seurat objects as input
MPES2.anchors <- FindIntegrationAnchors(object.list = MPES2, reduction = "cca", anchor.features = features,dim=1:20)  
  ## Scaling features for provided objects  
  ## Finding all pairwise anchors  
  ## Running CCA  
  ## Merging objects  
  ## Finding neighborhoods  
  ## Finding anchors  
  ##  Found 1249 anchors  
  ## Filtering anchors  
  ##  Retained 220 anchors  
  MPES2.combined <- IntegrateData(anchorset = MPES2.anchors,dim=1:20)  
  ## Merging dataset 1 into 2  
  ## Extracting anchors for merged samples  
  ## Finding integration vectors  
  ## Finding integration vector weights  
  ## Integrating data  
  MPES2.combined <- ScaleData(MPES2.combined, verbose = FALSE)  
  # assign levels
ClusMPES2_levels <- c('0', '1', '2', '3', '4', '5', '6', 'Early 2C and Zygote', 'Mid-Late 2 Cell', '4 Cell', '8 Cell', '8-16 Cell', '16 Cell', 'Early-Mid Blastocyst', 'Mid-Late Blastocyst','MEF')
levels(MPES2.combined) <- ClusMPES2_levels

MPES2.combinedavgexp <- AverageExpression(MPES2.combined, return.seurat = T)  
  ## Warning: The following arguments are not used: row.names  
  ## Centering and scaling data matrix  
  # totipotent genes  plots
VlnPlot(MPES2.combined, features = c("Zscan4c","Zscan4d","Rxra"),ident=c('0', '1', '2', '3', '4', '5', '6', 'Early 2C and Zygote', 'Mid-Late 2 Cell', '4 Cell', '8 Cell', '8-16 Cell', '16 Cell', 'Early-Mid Blastocyst', 'Mid-Late Blastocyst'),assay='integrated')  
   
  # Exclude MEF from dataset by subsetting 
MPES2.combined1<-subset(x = MPES2.combined, idents = c('0', '1', '2', '3', '4', '5', '6', 'Early 2C and Zygote', 'Mid-Late 2 Cell', '4 Cell', '8 Cell', '8-16 Cell', '16 Cell', 'Early-Mid Blastocyst', 'Mid-Late Blastocyst'))

# Correlation heatmap of TBLC clusters vs early mouse embryo
# MEF is removed as it is not a cell type of interest
av.exp <- AverageExpression(MPES2.combined1)$integrated
matrix.av.exp <- as.data.frame(av.exp)
matrix.av.exp  
 
 
 
  cor.exp <- as.data.frame(cor(matrix.av.exp))
cor.exp$x <- rownames(cor.exp)
cor.df <- tidyr::gather(data = cor.exp, y, correlation,  c('0', '1', '2', '3', '4', '5', '6', 'Early 2C and Zygote', 'Mid-Late 2 Cell', '4 Cell', '8 Cell', '8-16 Cell', '16 Cell', 'Early-Mid Blastocyst', 'Mid-Late Blastocyst'))

corClusMPES2_levels <- c('0', '1', '2', '3', '4', '5', '6', 'Early 2C and Zygote', 'Mid-Late 2 Cell', '4 Cell', '8 Cell', '8-16 Cell', '16 Cell', 'Early-Mid Blastocyst', 'Mid-Late Blastocyst')

ggplot(cor.df, aes(x= factor(x, level = corClusMPES2_levels), y=factor(y, level = corClusMPES2_levels), fill = correlation)) + geom_tile(color='white')+scale_fill_gradient(low="skyblue", high="red")+ theme(axis.text.x = element_text(angle = 90, vjust = 0.5, hjust=1))  
   
  # hierarchical clustering tree of TBLC clusters vs early mouse embryo
# considering first 20 PCs

MPES2.combined1<-subset(x = MPES2.combined, idents = c('0', '1', '2', '3', '4', '5', '6', 'Early 2C and Zygote', 'Mid-Late 2 Cell', '4 Cell', '8 Cell', '8-16 Cell', '16 Cell', 'Early-Mid Blastocyst', 'Mid-Late Blastocyst'))

tree2<-BuildClusterTree(MPES2.combined1,assay='integrated')
tree2  
  ## An object of class Seurat 
## 23985 features across 4512 samples within 2 assays 
## Active assay: integrated (2000 features, 2000 variable features)
##  1 other assay present: RNA  
  PlotClusterTree(tree2,direction="rightwards")  
   
  # pull the tree
tree2 <- Tool(object = tree2, slot = "BuildClusterTree")
# plot the tree
ape::plot.phylo(x = tree2, direction = "rightwards")  
   
  # find markers for every cluster compared to all remaining cells, report only the positive ones
AllClus.marker<-FindAllMarkers(TBLC, only.pos = TRUE, min.pct = 0.25, logfc.threshold = 0.5)  
  ## Calculating cluster 0  
  ## Calculating cluster 1  
  ## Calculating cluster 3  
  ## Calculating cluster 2  
  ## Calculating cluster 4  
  ## Calculating cluster MEF  
  ## Calculating cluster 5  
  ## Calculating cluster 6  
  AdjAllClus.marker<-subset(AllClus.marker, p_val_adj < 0.05)

# convert to dataframe and give column name
as.data.frame(AdjAllClus.marker)  
 
 
 
  colnames(AdjAllClus.marker)[0] <- "Gene_Symbol"
AdjAllClus.marker<-tibble::rownames_to_column(AdjAllClus.marker, "Gene_Symbol") 
AdjAllClus.marker  
 
 
 
  # sorting differential expression by fold change order
write_xlsx(AdjAllClus.marker,"~/Desktop/AdjAllClus.marker.xlsx")  
  # heatmap of all TBLCs cluster marker
DoHeatmap(MPES2.combinedavgexp, features = AdjAllClus.marker$gene[c(1:802,1181:1461)], hjust=0.5,angle=0, size=4,group.bar.height = 0.01, draw.lines = FALSE, cells=c('0', '1', '2', '3', '4', '5', '6', 'Early 2C and Zygote', 'Mid-Late 2 Cell'), disp.max = 1000, disp.min = -1000)  
  ## Warning in DoHeatmap(MPES2.combinedavgexp, features =
## AdjAllClus.marker$gene[c(1:802, : The following features were omitted as they
## were not found in the scale.data slot for the integrated assay: Dnaja2, Mrpl28,
## Bex4, Alyref, Dut, Psma6, Nsmce4a, Marcksl1, Slc25a4, Phlda3, Mrfap1, Stmn1,
## Ptges3, Psma4, Anp32a, Eif3i, Srsf6, Capzb, Hdgf, Fbl, Snrpa1, Fkbp4, Taf7,
## Prdx6, Clta, Rbm3, Glrx3, Hnrnpc, Gm8797, Set, Eif3g, Arf1, Dhx16, Psmb1,
## Atp5d, Cdc42, Psma5, Eif6, Cox7a2l, Magoh, Mrpl15, Psmb5, Sumo2, Ywhaq, Vdac1,
## Anp32b, Psmb3, Rbm8a, Calm2, Srsf7, Ywhae, Tagln2, Rsl24d1, Tpi1, Psmb4, Cdk1,
## H3f3a, Cnbp, Hmgb2, Pebp1, Ncl, Psmb6, Eef2, H2afz, Srsf3, Eef1d, Eif1, Psmb2,
## Pgam1, Npm1, Rpsa, Actg1, Hspb1, Rps2, Eif5a, Rpl10, Ppia, Ran, Gapdh, Eef1a1,
## Ubb, Prdx1, Tuba1c, Pdlim1, Marc2, Atp6v0b, Chic2, Cystm1, Gsr, Arpc2, Crip2,
## Clic1, Morf4l2, Chmp4b, Cltc, Apoc1, Tmed2, Mfsd1, Atxn7l3b, Ctsc, Galk1,
## Sec61a1, Flnb, Vamp8, Calm1, Akap12, Snx2, Reep5, Slc38a1, Gga2, Arih2, Surf4,
## Tbx3, Cndp2, Lrrc59, Cdipt, Marcks, Ctsa, Plscr1, Tax1bp3, P4hb, Colgalt1,
## Tulp4, Agpat4, Lmna, Plod1, Elf3, Slc17a5, Klhl2, Fam213a, Cmtm7, Gas6, Rcn3,
## Pros1, Hexa, Efna4, Fam129b, F2r, P4ha1, Hspg2, Wdfy1, Rrbp1, Ckap4, Txndc12,
## Emp2, Myo6, Me1, Phldb2, Sapcd1, Dok2, Nrg2, Slc7a6, Gdpd5, Basp1, Cyb5r3,
## Zcrb1, Sub1, Perp, Tceal9, Gpx1, Hcfc1r1, Zfp706, Bex3, Bex1, 2010107E04Rik,
## Tcea3, Gm2694, St13, Sec23b, Hnrnpa0, Ndufv3, Cox7a1, Mrpl42, Syce2, Rwdd1,
## Uchl3, Srp19, Dph3, Myl4, Parp1, Gm2000, Mrpl52, Lsm7, Lsm5, Mrpl33, Timm8b,
## Uqcrq, Cox7a2, Tmem256, Cks1b, Atp5k, Mrps21, Cox7b, Rps27rt, Tomm5, Polr2l,
## Rpl5, Hmgn1, Hnrnpa2b1, Hmgn2, Uqcr10, Smim11, Ndufa2, Gm48168, Dpy30, Atp5j,
## Rpl39, Lsm6, Pdap1, Hk2, Snrpg, Cox17, Rps15a, Rpl31, Bola2, Ost4, Atp5g1,
## Tomm7, Rps17, mt-Nd2, Rpl22l1, Elob, Tmsb10, Rpl34, Dnajc19, Atp5e, Rpl26,
## Atox1, Rps11, Rpl36a, H2afv, Rpl32, Sem1, Rps23, Gm10076, Tma7, Rps18, Rps27,
## Rps19, Rpl35, Rpl35a, Rpl36, Rps27l, Rpl37a, Rps29, Rpl37, Rpl41, Rps28, Ppm1a,
## Csnk2a1, Kpna2, Ate1, Dnajc3, Srsf5, Cand1, Nop14, Eif4a2, Polr2a, Cyp2s1,
## H1fx, Snrpc, Zcchc17, Gtf2b, Fmr1nb, Maged1, Arid4a, Atp6v1e1, Igfbp3, Ctr9,
## Mtf2, Ube2t, Dbr1, Iars, Tmem189, Rplp0, Dcbld1, Lmbr1l, Mcl1, 2810004N23Rik,
## Utf1, Ndufs6, Rnf7, Rpl17, Rps26, Rps12, Dppa5a, Ptma, Rplp1, Aire, Kat6b,
## Phc1, AC149090.1, Tead1, Hnrnpl, Etnk1, Upf3b, Trip12, Aebp2, Supt20, Epha4,
## Fbxo2, Cbx5, Bptf, 2810474O19Rik, Fkbp9, mt-Nd4l, March7, Tanc1, Mcm4, Chka,
## Strn3, Tmem97, Sntb2, Rfc4, Golga4, Polg, BC005624, Nfib, Hmgcr, Smg5, Dis3,
## Fdft1, Epha2, Igf2bp3, Kansl1, Ruvbl1, Gm48170, Atrx, Phgdh, Mafg, Leo1, Cep44,
## 2700097O09Rik, Safb2, Golgb1, Hspbap1, Umps, Pbrm1, Ssr1, Lsg1, Pum3, Itga6,
## Slc6a6, Mcm2, Lig1, R3hdm1, Hnrnpr, Xpo1, Topbp1, Eftud2, Manf, Slc38a9, Igbp1,
## Kif20a, Skiv2l2, Supt16, Serpine2, Mrpl19, Rsbn1l, Nvl, Slc35b1, Ddb1, Slc25a36,
## Atp2a2, Jmjd1c, Creld2, Tmem41b, Smc3, Lonp1, Pcmt1, Psmd11, Triml2, Glmp,
## Ctnna1, Top2a, Rrm2, Brd8, Acadvl, Sf3a3, Slc20a1, Stx16, Tra2a, Lap3, Eed,
## Ssrp1, Clcn3, Sptbn1, Trap1, Dtl, Pa2g4, Spns1, Degs1, Hnrnpm, Tuba1a, Serinc1,
## Srrt, Srsf2, Lbr, Yif1b, Macf1, Usp14, Zdhhc20, Copb2, Pak1ip1, Prpf40a, Naa35,
## Glg1, Col18a1, Matr3, Farsa, Cep192, Sun1, Pnn, Fdps, Apex1, Kif22, Prkcsh,
## Gfm1, Ktn1, Srsf10, Luc7l2, Hspa14, Ckap2, M6pr, Nup93, Wdr18, Gon4l, Man2b1,
## Mphosph10, Gmps, Gm26825, Mbnl2, Akr1b3, Ddx46, Ftsj3, Polr2b, Mybbp1a, Ticrr,
## Mat2a, Acvr2b, Mphosph8, Plod2, Puf60, Gm47283, Mcm5, Pfkl, Dap3, Hmces, Steap1,
## Ppp1r12a, Kank3, Cabin1, Ewsr1, Pmpcb, Ccnb1, Ndufs1, Usp48, Pid1, Smad7, Top1,
## Psmc5, Mcm6, Ash2l, Hsd17b12, Eif4a3, Rbmxl2, Mau2, Nktr, Eif3e, Rsl1d1, Afg3l1,
## Msmo1, Nmd3, Ahsa1, Lman2, Bfar, Clpb, Ddx39, Atp2b1, Laptm4a, Sptlc2, Utp4,
## Mrps18b, Mesd, Ssb, Psmd3, Nsun2, Slc29a1, Phip, mt-Nd5, mt-Nd3, Mycbp2, Msh2,
## Nemf, Pdhb, Rcc2, Samm50, Ogt, Kifc1, Suclg1, Dnajb11, Pecam1, Dnajc7, Stk24,
## Ndufv1, Tsix, Aco2, Bub3, Slc7a7, 4833420G17Rik, Bora, Mars, Arl6ip1, Tyms,
## Stt3a, Naa25, Ppid, Ddx18, Ppib, Tomm40, Zfp266, Terf1, Tra2b, Nono, Stip1,
## Gdf3, Rars, Hspa4, Nedd4, Snrnp48, Mfge8, Mfsd10, Dnttip2, Tex19.1, Atp6ap2,
## Dnaja1, Gars, Tmem165, Eif4g1, AU018091, Mdh1, Psmd13, Eif3c, Prmt1, Hnrnpu,
## Ndufa10, Tet1, Sf3b2, Ckb, Pdia4, C1qbp, Brix1, Aldh2, Fh1, Hnrnpf, Pura, Hexb,
## Rsrp1, Ppm1g, Rbpms, Nectin2, Mcm3, Gm26699, Ndufs2, Snhg11, Cdc5l, Prdx4,
## Ebna1bp2, Sbspon, Sdha, Psmc4, Acp6, Rpn1, Ruvbl2, Lman1, Psmc2, Eif3l, Bclaf1,
## Krit1, Trp53, Dnmt3l, Eif3a, Pdia3, Ddx39b, Jarid2, Psmd1, Ilf2, Frrs1, Psmd6,
## Gart, Uqcrc2, Hat1, Ncor1, Impdh2, Gle1, Psmd12, Adh5, Ttc19, Mcm7, Grn, Tkt,
## Slc38a4, Msh6, Spcs2, Ube3a, Rtn4, Kif5b, Ndufa9, Smu1, Phb2, Alkbh1, Ccnt2,
## Malat1, Tuba1b, Uba2, Cyc1, Atp1a1, Ddx50, Spint2, Psmd2, Tmbim6, Gspt1, Tmem59,
## Tubb5, Rbm25, Klf5, Psmd7, Ddost, Vmp1, Canx, Pcolce, Pfkp, Eif3d, Rbm4b, Pnisr,
## Gm26917, Immt, G3bp2, Fus, Hspa9, G3bp1, Pabpc1, Cct2, Ass1, Aplp2, Cct5, Eprs,
## Trim35, Slc16a3, Ssr2, Klf9, Gnl3, Fubp1, Tecr, Ddx5, Tubb4b, Psma1, Lamp1,
## Cct4, Dkc1, Taldo1, Slc25a5, Nid2, Vcp, Ankrd11, Rbm26, Trim28, Calr, Gpi1,
## Ndufb9, Actl6a, Trap1a, Ctbp2, Slc25a3, Hnrnpk, Snrnp70, Bsg, Mtdh, Son, Srsf11,
## mt-Co2, Hsp90ab1, mt-Atp6, Tmed9, Epcam, Slc3a2, Ttc14, Pgk1, Uqcrc1, Aldoa,
## Nop56, Ldha, Cct8, Hsp90b1, Cox6a1, mt-Nd4, Bcap31, Rpn2, Cct3, Atp5a1, Srrm2,
## Hspd1, Sf3b3, Sf3b1, Cct7, Hspa8, Slc2a3, mt-Co1, Cd81, Eno1, Mdh2, Eif4a1, Pkm,
## Pdia6, mt-Co3, Cd9, mt-Cytb, Atp5b, mt-Nd1, Slc2a1  
  ## Warning: Removed 7 rows containing missing values (geom_text).  
   
  # find downregulated genes in Cluster 3 vs rest clusters (include early deve)
clus3vsrest.markers <- FindMarkers(MPES2.combined, ident.1 = "3", min.pct = 0.25)
clus3vsrest.markers  
 
 
 
  Adjclus3vsrest <-subset(clus3vsrest.markers, p_val_adj < 0.05)

# convert to dataframe and give column name
as.data.frame(Adjclus3vsrest)  
 
 
 
  colnames(Adjclus3vsrest)[0] <- "Gene_Symbol"
Adjclus3vsrest<-tibble::rownames_to_column(Adjclus3vsrest, "Gene_Symbol") 
Adjclus3vsrest  
 
 
 
  # sorting differential expression by fold change order
LowerAdjclus3vsrest<-Adjclus3vsrest[order(Adjclus3vsrest$avg_log2FC),]
HigherAdjclus3vsrest<-Adjclus3vsrest[order(-Adjclus3vsrest$avg_log2FC),]
library("writexl")
write_xlsx(HigherAdjclus3vsrest,"~/Desktop/HigherAdjTBLCcluster3.markers.xlsx")
write_xlsx(LowerAdjclus3vsrest,"~/Desktop/LowerAdjTBLCcluster3.markers.xlsx")  
  # heat map of downregulated genes in Clus3 vs rest clusters (include early deve)
DoHeatmap(MPES2.combinedavgexp, features = LowerAdjclus3vsrest$Gene_Symbol[1:75],hjust=0.5, angle=0, size=3, group.bar.height = 0.01,draw.lines = FALSE,cells=c('0', '1', '2', '3', '4', '5', '6', 'Early 2C and Zygote', 'Mid-Late 2 Cell'))  
  ## Warning: Removed 7 rows containing missing values (geom_text).  
   
 Supplementary: Integration (CCA) with avg TBLC and ESCs 
  # cluster TBLC at a greater resolution
TBLC <- FindClusters(TBLC, resolution = 0.5)  
  ## Modularity Optimizer version 1.3.0 by Ludo Waltman and Nees Jan van Eck
## 
## Number of nodes: 4534
## Number of edges: 152115
## 
## Running Louvain algorithm...
## Maximum modularity in 10 random starts: 0.8532
## Number of communities: 8
## Elapsed time: 0 seconds  
  # Run non-linear dimentional reduction (UMAP/tSNE)
# If you haven't installed UMAP, you can do so via reticulate::py_install(packages =
# 'umap-learn')
TBLC <- RunUMAP(TBLC, dims = 1:15)  
  ## 15:47:12 UMAP embedding parameters a = 0.9922 b = 1.112  
  ## 15:47:12 Read 4534 rows and found 15 numeric columns  
  ## 15:47:12 Using Annoy for neighbor search, n_neighbors = 30  
  ## 15:47:12 Building Annoy index with metric = cosine, n_trees = 50  
  ## 0%   10   20   30   40   50   60   70   80   90   100%  
  ## [----|----|----|----|----|----|----|----|----|----|  
  ## **************************************************|
## 15:47:13 Writing NN index file to temp file /var/folders/cv/cfhg7t_j5y3d34y2_81gskv00000gn/T//RtmpdT6yOL/file4d31340d5886
## 15:47:13 Searching Annoy index using 1 thread, search_k = 3000
## 15:47:14 Annoy recall = 100%
## 15:47:14 Commencing smooth kNN distance calibration using 1 thread
## 15:47:15 Initializing from normalized Laplacian + noise
## 15:47:15 Commencing optimization for 500 epochs, with 186234 positive edges
## 15:47:21 Optimization finished  
  # Assigning cluster names for future reference and data integration
new.cluster.ids <- c("0","1","3","2","4","MEF","5","6")
names(new.cluster.ids) <- levels(TBLC)
TBLC <- RenameIdents(TBLC, new.cluster.ids)  
  #Clustering Cells
ESC <- FindNeighbors(ESC, dims = 1:20)  
  ## Computing nearest neighbor graph  
  ## Computing SNN  
  ESC <- FindClusters(ESC, resolution = 1)  
  ## Modularity Optimizer version 1.3.0 by Ludo Waltman and Nees Jan van Eck
## 
## Number of nodes: 4139
## Number of edges: 135955
## 
## Running Louvain algorithm...
## Maximum modularity in 10 random starts: 0.7029
## Number of communities: 14
## Elapsed time: 0 seconds  
  # Run non-linear dimentional reduction (UMAP/tSNE)
# If you haven't installed UMAP, you can do so via reticulate::py_install(packages =
# 'umap-learn')
ESC <- RunUMAP(ESC, dims = 1:20)  
  ## 15:47:23 UMAP embedding parameters a = 0.9922 b = 1.112  
  ## 15:47:23 Read 4139 rows and found 20 numeric columns  
  ## 15:47:23 Using Annoy for neighbor search, n_neighbors = 30  
  ## 15:47:23 Building Annoy index with metric = cosine, n_trees = 50  
  ## 0%   10   20   30   40   50   60   70   80   90   100%  
  ## [----|----|----|----|----|----|----|----|----|----|  
  ## **************************************************|
## 15:47:24 Writing NN index file to temp file /var/folders/cv/cfhg7t_j5y3d34y2_81gskv00000gn/T//RtmpdT6yOL/file4d3145e8c559
## 15:47:24 Searching Annoy index using 1 thread, search_k = 3000
## 15:47:25 Annoy recall = 100%
## 15:47:25 Commencing smooth kNN distance calibration using 1 thread
## 15:47:25 Initializing from normalized Laplacian + noise
## 15:47:25 Commencing optimization for 500 epochs, with 168118 positive edges
## 15:47:31 Optimization finished  
  DimPlot(ESC, reduction = "umap", label = TRUE)  
   
  # Assigning cluster names for future reference and data integration
ESC.cluster.ids <- c("ESCs","ESCs","ESCs","ESCs","ESCs","ESCs","ESCs","ESCs","ESCs","ESCs","ESCs","ESCs","ESCs","2CLC")
names(ESC.cluster.ids) <- levels(ESC)
ESC <- RenameIdents(ESC, ESC.cluster.ids)
DimPlot(ESC, reduction = "umap", label = TRUE) + NoLegend()  
   
  # merging TBLC and ESC data
ESCTBLC<-merge(TBLC,ESC)  
  ## Warning in CheckDuplicateCellNames(object.list = objects): Some cell names are
## duplicated across objects provided. Renaming to enforce unique cell names.  
  # splitting object identify for downstream cluster analysis
ESCTBLC1 <- SplitObject(ESCTBLC, split.by = "orig.ident")
ESCTBLC1  
  ## $TBLC
## An object of class Seurat 
## 19038 features across 4534 samples within 1 assay 
## Active assay: RNA (19038 features, 0 variable features)
## 
## $ESC
## An object of class Seurat 
## 19038 features across 4139 samples within 1 assay 
## Active assay: RNA (19038 features, 0 variable features)  
  # normalize and identify variable features for each dataset independently
ESCTBLC1 <- lapply(X = ESCTBLC1, FUN = function(x) {
    x <- NormalizeData(x)
    x <- FindVariableFeatures(x, selection.method = "vst", nfeatures = 2000)
})

# integrating data by integrating 2 seurat objects as input
ESCTBLC1.anchors <- FindIntegrationAnchors(object.list = ESCTBLC1, dims = 1:20)  
  ## Computing 2000 integration features  
  ## Scaling features for provided objects  
  ## Finding all pairwise anchors  
  ## Running CCA  
  ## Merging objects  
  ## Finding neighborhoods  
  ## Finding anchors  
  ##  Found 12190 anchors  
  ## Filtering anchors  
  ##  Retained 4206 anchors  
  ESCTBLC1.combined <- IntegrateData(anchorset = ESCTBLC1.anchors, dims = 1:20)  
  ## Merging dataset 2 into 1  
  ## Extracting anchors for merged samples  
  ## Finding integration vectors  
  ## Finding integration vector weights  
  ## Integrating data  
  DefaultAssay(ESCTBLC1.combined) <- "integrated"

# Run the standard workflow for visualization and clustering
ESCTBLC1.combined <- ScaleData(ESCTBLC1.combined, verbose = FALSE)
ESCTBLC1.combined <- RunPCA(ESCTBLC1.combined, verbose = FALSE)
# TSNE and Clustering
ESCTBLC1.combined <- RunTSNE(ESCTBLC1.combined, reduction = "pca", dims = 1:20, check_duplicates = FALSE)  
  # assign levels
ClusESCTBLC1.combined_levels <- c('ESCs','2CLC','0', '1', '2', '3', '4', '5', '6','MEF')
levels(ESCTBLC1.combined) <- ClusESCTBLC1.combined_levels  
  # To visualize the two conditions side-by-side, we can use the split.by argument to show each condition colored by cluster
DimPlot(ESCTBLC1.combined, reduction = "tsne",group.by = "orig.ident")  
   
  DimPlot(ESCTBLC1.combined, reduction = "tsne")  
   
  # Interactive plot showing Zscan4c distribution
# NOTE: unavaile if viewed without R-studio
FeaturePlot(ESCTBLC1.combined, features = c("Zscan4c"), split.by = "orig.ident", max.cutoff = 3,reduction = "tsne",
    cols = c("grey","red"),min.cutoff = "q5")  
   
  FeaturePlot(ESCTBLC1.combined, features = c("Zscan4d"), split.by = "orig.ident", max.cutoff = 3,reduction = "tsne",
    cols = c("grey","red"),min.cutoff = "q5")  
   
  # Another non-overlapping population is MEF as viewed under MEF marker Sp100a4
FeaturePlot(ESCTBLC1.combined, features = c("S100a4"), split.by = "orig.ident", max.cutoff = 3,reduction = "tsne",
    cols = c("grey","red"),min.cutoff = "q5")  
   
  VlnPlot(ESCTBLC1.combined, features = c("Zscan4c","Zscan4d"),ident=c('ESCs','2CLC','0', '1', '2', '3', '4', '5', '6'),assay='integrated',pt.size=0)  
   
  VlnPlot(ESCTBLC1.combined, features = c("Klf4","Sox2"),ident=c('ESCs','2CLC','0', '1', '2', '3', '4', '5', '6'),assay='integrated',pt.size=0)  
   
 Reviewer’s suggestion regarding inclusion of ESCs 
  # cluster TBLC at a greater resolution
TBLC <- FindClusters(TBLC, resolution = 0.5)  
  ## Modularity Optimizer version 1.3.0 by Ludo Waltman and Nees Jan van Eck
## 
## Number of nodes: 4534
## Number of edges: 152115
## 
## Running Louvain algorithm...
## Maximum modularity in 10 random starts: 0.8532
## Number of communities: 8
## Elapsed time: 0 seconds  
  # Run non-linear dimentional reduction (UMAP/tSNE)
# If you haven't installed UMAP, you can do so via reticulate::py_install(packages =
# 'umap-learn')
TBLC <- RunUMAP(TBLC, dims = 1:15)  
  ## 15:51:19 UMAP embedding parameters a = 0.9922 b = 1.112  
  ## 15:51:19 Read 4534 rows and found 15 numeric columns  
  ## 15:51:19 Using Annoy for neighbor search, n_neighbors = 30  
  ## 15:51:19 Building Annoy index with metric = cosine, n_trees = 50  
  ## 0%   10   20   30   40   50   60   70   80   90   100%  
  ## [----|----|----|----|----|----|----|----|----|----|  
  ## **************************************************|
## 15:51:19 Writing NN index file to temp file /var/folders/cv/cfhg7t_j5y3d34y2_81gskv00000gn/T//RtmpdT6yOL/file4d3129f66bcc
## 15:51:19 Searching Annoy index using 1 thread, search_k = 3000
## 15:51:20 Annoy recall = 100%
## 15:51:20 Commencing smooth kNN distance calibration using 1 thread
## 15:51:21 Initializing from normalized Laplacian + noise
## 15:51:21 Commencing optimization for 500 epochs, with 186234 positive edges
## 15:51:27 Optimization finished  
  # Assigning cluster names for future reference and data integration
TBLCs.clustered.ids <- c("0","1","3","2","4","MEF","5","6")
names(TBLCs.clustered.ids) <- levels(TBLC)
TBLC <- RenameIdents(TBLC, TBLCs.clustered.ids)  
  # merging TBLC and in vivo mouse early development data
TBLCESC<-merge(TBLC,ESC)  
  ## Warning in CheckDuplicateCellNames(object.list = objects): Some cell names are
## duplicated across objects provided. Renaming to enforce unique cell names.  
  TBLCESCEarlyDeve<-merge(TBLCESC, EarlyDeve)
head(TBLCESCEarlyDeve@meta.data)  
 
 
 
  # splitting object identify for downstream cluster analysis
TBLCESCEarlyDeve1 <- SplitObject(TBLCESCEarlyDeve, split.by = "orig.ident")
TBLCESCEarlyDeve1  
  ## $TBLC
## An object of class Seurat 
## 22603 features across 4534 samples within 1 assay 
## Active assay: RNA (22603 features, 0 variable features)
## 
## $ESC
## An object of class Seurat 
## 22603 features across 4139 samples within 1 assay 
## Active assay: RNA (22603 features, 0 variable features)
## 
## $EarlyDevelopment
## An object of class Seurat 
## 22603 features across 259 samples within 1 assay 
## Active assay: RNA (22603 features, 0 variable features)  
  # normalize and identify variable features for each dataset independently
TBLCESCEarlyDeve1 <- lapply(X = TBLCESCEarlyDeve1, FUN = function(x) {
    x <- NormalizeData(x)
    x <- FindVariableFeatures(x, selection.method = "vst", nfeatures = 2000)
})

# select features that are repeatedly variable across datasets for integration
features <- SelectIntegrationFeatures(object.list = TBLCESCEarlyDeve1)
# integrating data by integrating 2 seurat objects as input
TBLCESCEarlyDeve1.anchors <- FindIntegrationAnchors(object.list = TBLCESCEarlyDeve1, reduction = "cca", anchor.features = features, dim=1:20)  
  ## Scaling features for provided objects  
  ## Finding all pairwise anchors  
  ## Running CCA  
  ## Merging objects  
  ## Finding neighborhoods  
  ## Finding anchors  
  ##  Found 12297 anchors  
  ## Filtering anchors  
  ##  Retained 3968 anchors  
  ## Running CCA  
  ## Merging objects  
  ## Finding neighborhoods  
  ## Finding anchors  
  ##  Found 1242 anchors  
  ## Filtering anchors  
  ##  Retained 1060 anchors  
  ## Running CCA  
  ## Merging objects  
  ## Finding neighborhoods  
  ## Finding anchors  
  ##  Found 1219 anchors  
  ## Filtering anchors  
  ##  Retained 1029 anchors  
  TBLCESCEarlyDeve1.combined <- IntegrateData(anchorset = TBLCESCEarlyDeve1.anchors,dim=1:20)  
  ## Merging dataset 3 into 1  
  ## Extracting anchors for merged samples  
  ## Finding integration vectors  
  ## Finding integration vector weights  
  ## Integrating data  
  ## Merging dataset 2 into 1 3  
  ## Extracting anchors for merged samples  
  ## Finding integration vectors  
  ## Finding integration vector weights  
  ## Integrating data  
  # perform integrated analysis
DefaultAssay(TBLCESCEarlyDeve1.combined) <- "integrated"

# Run the standard workflow for visualization and clustering
TBLCESCEarlyDeve1.combined <- ScaleData(TBLCESCEarlyDeve1.combined, verbose = FALSE)
TBLCESCEarlyDeve1.combined <- RunPCA(TBLCESCEarlyDeve1.combined, npcs = 30, verbose = FALSE)
# UMAP and Clustering
TBLCESCEarlyDeve1.combined <- RunUMAP(TBLCESCEarlyDeve1.combined, reduction = "pca", dims = 1:20)  
  ## 15:55:10 UMAP embedding parameters a = 0.9922 b = 1.112  
  ## 15:55:10 Read 8932 rows and found 20 numeric columns  
  ## 15:55:10 Using Annoy for neighbor search, n_neighbors = 30  
  ## 15:55:10 Building Annoy index with metric = cosine, n_trees = 50  
  ## 0%   10   20   30   40   50   60   70   80   90   100%  
  ## [----|----|----|----|----|----|----|----|----|----|  
  ## **************************************************|
## 15:55:11 Writing NN index file to temp file /var/folders/cv/cfhg7t_j5y3d34y2_81gskv00000gn/T//RtmpdT6yOL/file4d3150d777bd
## 15:55:11 Searching Annoy index using 1 thread, search_k = 3000
## 15:55:13 Annoy recall = 100%
## 15:55:13 Commencing smooth kNN distance calibration using 1 thread
## 15:55:14 Initializing from normalized Laplacian + noise
## 15:55:14 Commencing optimization for 500 epochs, with 377980 positive edges
## 15:55:25 Optimization finished  
  #TBLCESCEarlyDeve1.combined <- FindNeighbors(TBLCESCEarlyDeve1.combined, reduction = "pca", dims = 1:20)
#TBLCESCEarlyDeve1.combined <- FindClusters(TBLCESCEarlyDeve1.combined, resolution = 1)
#visualize UMAP
DimPlot(TBLCESCEarlyDeve1.combined, reduction = "umap", group.by = "orig.ident")  
   
  # assign levels and dimplot
clustered_levels <- c('0','1','2','3','4','5','6','ESCs','2CLC', 'Early 2C and Zygote', 'Mid-Late 2 Cell', '4 Cell', '8 Cell', '8-16 Cell', '16 Cell', 'Early-Mid Blastocyst', 'Mid-Late Blastocyst','MEF')
levels(TBLCESCEarlyDeve1.combined) <- clustered_levels

DimPlot(TBLCESCEarlyDeve1.combined, reduction = "umap",label=TRUE,repel=TRUE)  
   
  TBLCESCEarlyDeve1.combinedavgexp <- AverageExpression(TBLCESCEarlyDeve1.combined, return.seurat = T)  
  ## Warning: The following arguments are not used: row.names  
  ## Centering and scaling data matrix  
  # Violin plots including ESCs
# totipotent gene and pluripotent gene
VlnPlot(TBLCESCEarlyDeve1.combined, features = c("Zscan4c","Zscan4d","Rxra","Pou5f1","Sox2","Nanog"),ident=c('ESCs','2CLC','0', '1', '2', '3', '4', '5', '6', 'Early 2C and Zygote', 'Mid-Late 2 Cell', '4 Cell', '8 Cell', '8-16 Cell', '16 Cell', 'Early-Mid Blastocyst', 'Mid-Late Blastocyst'),assay='integrated',pt.size=0)  
  ## Warning: Could not find Pou5f1 in the default search locations, found in RNA
## assay instead  
   
  av.exp.forreviewer <- AverageExpression(TBLCESCEarlyDeve1.combined)$`integrated`
matrix.av.expav.exp.forreviewer <- as.data.frame(av.exp.forreviewer)
colnames(matrix.av.expav.exp.forreviewer)[0] <- "Gene_Symbol"
matrix.av.expav.exp.forreviewer<-tibble::rownames_to_column(matrix.av.expav.exp.forreviewer, "Gene_Symbol") 
matrix.av.expav.exp.forreviewer  
 
 
 
  write_xlsx(matrix.av.expav.exp.forreviewer,"~/Desktop/Scaledexpressionmatrix.xlsx")  
 Repeat result from Figure S3F 
  FeaturePlot(ESCTBLC1.combined, features = c("Rest","Utf1"), split.by = "orig.ident",reduction = "tsne",
    cols = c("grey","red"),min.cutoff = "q5")  
  ## Warning: Could not find Rest in the default search locations, found in RNA assay
## instead  
   
  FeaturePlot(ESCTBLC1.combined, features = c("Tcf15","H2afx","Aes"), split.by = "orig.ident",reduction = "tsne", cols = c("grey","red"),min.cutoff = "q5")  
  ## Warning: Could not find Aes in the default search locations, found in RNA assay
## instead  
   
  FeaturePlot(ESCTBLC1.combined, features = c("Ctsd","Cdkn1a"), split.by = "orig.ident",reduction = "tsne", cols = c("grey","red"),min.cutoff = "q5")  
   
 Repeat result from Figure S3H with average TBLCs 
  # cluster TBLC at a low resolution
TBLC <- FindClusters(TBLC, resolution = 0.01)  
  ## Modularity Optimizer version 1.3.0 by Ludo Waltman and Nees Jan van Eck
## 
## Number of nodes: 4534
## Number of edges: 152115
## 
## Running Louvain algorithm...
## Maximum modularity in 10 random starts: 0.9915
## Number of communities: 2
## Elapsed time: 0 seconds  
  # Run non-linear dimentional reduction (UMAP/tSNE)
# If you haven't installed UMAP, you can do so via reticulate::py_install(packages =
# 'umap-learn')
TBLC <- RunUMAP(TBLC, dims = 1:15)  
  ## 15:55:41 UMAP embedding parameters a = 0.9922 b = 1.112  
  ## 15:55:41 Read 4534 rows and found 15 numeric columns  
  ## 15:55:41 Using Annoy for neighbor search, n_neighbors = 30  
  ## 15:55:41 Building Annoy index with metric = cosine, n_trees = 50  
  ## 0%   10   20   30   40   50   60   70   80   90   100%  
  ## [----|----|----|----|----|----|----|----|----|----|  
  ## **************************************************|
## 15:55:41 Writing NN index file to temp file /var/folders/cv/cfhg7t_j5y3d34y2_81gskv00000gn/T//RtmpdT6yOL/file4d315bb01206
## 15:55:41 Searching Annoy index using 1 thread, search_k = 3000
## 15:55:42 Annoy recall = 100%
## 15:55:42 Commencing smooth kNN distance calibration using 1 thread
## 15:55:43 Initializing from normalized Laplacian + noise
## 15:55:43 Commencing optimization for 500 epochs, with 186234 positive edges
## 15:55:48 Optimization finished  
  # Assigning cluster names for future reference and data integration
TBLCs.clustered.ids <- c("TBLCs","MEF")
names(TBLCs.clustered.ids) <- levels(TBLC)
TBLC <- RenameIdents(TBLC, TBLCs.clustered.ids)

# merging TBLC and ESC data
ESCTBLC<-merge(TBLC,ESC)  
  ## Warning in CheckDuplicateCellNames(object.list = objects): Some cell names are
## duplicated across objects provided. Renaming to enforce unique cell names.  
  # splitting object identify for downstream cluster analysis
ESCTBLC1 <- SplitObject(ESCTBLC, split.by = "orig.ident")
ESCTBLC1  
  ## $TBLC
## An object of class Seurat 
## 19038 features across 4534 samples within 1 assay 
## Active assay: RNA (19038 features, 0 variable features)
## 
## $ESC
## An object of class Seurat 
## 19038 features across 4139 samples within 1 assay 
## Active assay: RNA (19038 features, 0 variable features)  
  # normalize and identify variable features for each dataset independently
ESCTBLC1 <- lapply(X = ESCTBLC1, FUN = function(x) {
    x <- NormalizeData(x)
    x <- FindVariableFeatures(x, selection.method = "vst")
})

# integrating data by integrating 2 seurat objects as input
ESCTBLC1.anchors <- FindIntegrationAnchors(object.list = ESCTBLC1, dims = 1:20)  
  ## Computing 2000 integration features
## Scaling features for provided objects
## Finding all pairwise anchors
## Running CCA
## Merging objects
## Finding neighborhoods
## Finding anchors
##  Found 12190 anchors
## Filtering anchors
##  Retained 4206 anchors  
  ESCTBLC1.combined <- IntegrateData(anchorset = ESCTBLC1.anchors, dims = 1:20,features.to.integrate = c("Nr0b1","Sox2","Nanog","Tdgf1","H2afz","Tubb5","Pou5f1","Nop10","Zfp42","Tcf15","Tet1","Esrrb","Plk2","Cdkn1a","Zfp365","Trp53inp1","Mdm2","Btg2","Ddit4l","Pdrg1","Pid1","Emp3","Dffb","Ctsb"),verbose=FALSE)  
  ## Warning: Not all features provided are in this Assay object, removing the
## following feature(s): Gm5662, Pbld1, Srgn, Gm8300, Lefty1, H19, Gm5039,
## Olfr1369-ps1, Zscan4-ps2, Gm4027, Tmem80, Fry, Gm8994, Olfr889, Ptcra, Agl,
## Lgals1, Cyp4f14, Gm26870, Gm15879, Gm29666, Aldh1a3, Gm21761, Serpinh1,
## Zscan4c, Nlrc3, Zscan4d, Inhba, Fabp3, Gm29773, AF067061, Retn, Tagln, Noa1,
## BC147527, Gm45184, Gm8332, Foxq1, S100a4, Ctsl, Lhfpl4, Wdcp, Dab2, Plk4,
## Mcpt8, Gm43409, Slamf1, Gm4340, Acta2, Cryab, Meg3, Gm21762, Actb, S100a6,
## Gm2016, Alox8, Xist, Spp1, Krt18, Col4a1, Tcstv3, Spry2, Olfr1423, Zscan4-
## ps3, Rhox6, S100a3, Gm2022, Vim, Lefty2, Cdkn1c, Gm26737, Rrm2b, Spata31d1a,
## Tmsb4x, Xaf1, BC080695, Gata6, Usp17lc, AC160336.1, Thbs1, Gm45509, Bgn,
## Igfbp7, Gfod2, 4930548H24Rik, Amn, Col4a2, Krt8, Aqp8, Timp2, Id3, Hmga2,
## Mt1, Gm428, Tulp3, Ccnd2, Trh, Sparc, Grem1, Atg2a, A330069E16Rik, Dqx1, P3h3,
## Id1, Lama1, Aebp1, Glipr2, Hist1h2bc, Lgals4, Gm13075, Spata7, P2rx4, Rhox5,
## Unc13d, A930014E10Rik, Fst, 1010001N08Rik, AC163720.3, Sox17, Tc2n, Alpk2, Mt2,
## Igfbp4, Fbln2, Dkk1, Gm26909, Sp110, Twist2, Gm48754, Lrpap1, Cavin2, Rpl29,
## 6330410L21Rik, Fgfbp1, Apoe, Tm4sf1, Stmn2, Sfn, Gpx3, Usp17la, U90926, Gm12794,
## Pim1, A330032B11Rik, Gm10457, Fam25c, Lgals3, Gm13119, Krt7, Fbp2, Lamb1,
## Ifitm1, Gng11, Tpm2, Gata4, Tpm1, Calml4, Cyr61, 9330136K24Rik, Ccser1, Prex2,
## Bc1, Gm26710, Ccl7, Ifitm3, Fgf1, Cited4, Gm47510, Slc7a11, Csf1, P4ha2, Inpp4b,
## Col1a1, Dkk2, Comt, Limch1, Zscan4-ps1, Hspa5, Krtdap, Lgi1, Dennd5b, Serpine1,
## Hbegf, Lox, Oaf, AC125149.3, Ddit4, Gm48799, Cald1, Gm26782, Id2, Mylpf, Tpm4,
## Gm48610, 5430402O13Rik, Sdc4, Amt, Hmox1, AU019990, Serpinb2, Ngb, Gm13078,
## Scamp1, Nupr1, Siglec1, Ppbp, Fxyd3, Gm11238, Sp140, Calcoco2, Rpl39l, Cbx3,
## AC140186.1, Lmx1a, Neat1, Filip1l, C730034F03Rik, Usp26, Il11, Pth1r, Pou3f1,
## S100a13, Gm48764, Cd63, Prrx1, Ptgs2, Gm38947, Ascl2, Slc39a1, Gm26772, Chpt1,
## Gm26764, Sct, Ccl20, Hist1h2ap, Nfkbia, Pdgfrl, Phlda1, A530040E14Rik, Ccl3,
## B2m, Lrp2, Ctgf, Shkbp1, Hs3st1, Ier3, Car2, Gm8953, Gadd45g, Bex6, Cxcl1, Gsn,
## Ercc4, Gm20625, H2-T23, Dlg1, Cubn, Sox4, 4933440M02Rik, Gm43263, BC028528,
## Krt17, Terf2ip, Myl9, Tmem252, Gm16368, Ngf, Runx1, Enah, Fth1, Klf2, Ccl2,
## Tmem92, Dppa3, Cd44, Ube2c, Adcy2, Epop, Ncoa3, H1f0, Rps24, Gpx2, Gjb3, Txnip,
## Hsp90aa1, Cited2, Parvb, Fam46a, Cbr3, Rab33b, Avpi1, B020031M17Rik, Igfbp2,
## Mafb, Glrx2, Phkg1, Fos, Spink1, S100a11, 1810062G17Rik, Phlda2, Serpinb5,
## Gm2056, Dpys, Rep15, Lrrc32, Plaur, Bbs4, Gm21731, Dusp1, Il1rl1, Gm12280,
## Gpx4, Cav1, Tfap2c, Gabarapl2, Stra8, Oasl1, Gsto1, Klf4, Slc15a1, Ccdc80,
## Gm10827, Ubtfl1, Ifit1, Crip1, Jun, Gmcl1, Aqp3, Junb, Arhgef26, Marveld1,
## Krt19, Krt42, Anxa2, Timp1, Gm35339, Psmb8, Rnf128, Col5a2, Axl, 0610040J01Rik,
## Aard, Chrm3, Sgk1, 2200002D01Rik, Anxa1, Nodal, Rhox9, Gm20442, Pyy, AC191865.2,
## Gm46332, Itm2b, Htra1, G0s2, Myo1f, Cidea, Zfp296, Cenpf, Fgf3, Atf4, Col1a2,
## Adm, Bmp4, Sfmbt2, Ifi27l2a, Gm28940, H2-D1, Prrx2, Rasgrp2, Fbxo15, Gm12800,
## Cdkn2a, Tnfsf12, Gm21269, Pirb, Zar1, Tinagl1, Cxcl5, Gbp2b, Platr3, Scaf11,
## Arg2, Car4, Exosc2, Msc, Chac1, Tmem176b, Cst3, Rhox13, Gm15627, Isg15, Plk3,
## AC158554.1, Pdgfra, AC168977.1, Podxl, 9530059O14Rik, Irgm1, Dazl, Gm16233,
## Perp, H2afx, Zfp809, Gm5788, Slc7a3, Bnip3, Apobec2, Slc28a3, Skil, Ahnak2,
## Foxo3, Ly6a, Zfp428, Clmn, Slc24a5, Ccnd1, Plac8, Nefl, Pla2g12b, Arhgdib,
## Upp1, Anxa5, Gbx2, Drr1, Wtap, Khdc3, Malat1, 2610528J11Rik, 1600010M07Rik,
## Endov, Ccdc163, Foxa2, Klf6, Mycn, Nr2f2, Hook2, Utp11, Dok2, Ubald2, Wnt4,
## Wfdc2, Fxyd6, Cdx2, Fam186b, Fabp5, Gadd45a, S100a10, Flrt3, Nat8f2, Ooep,
## Ebf1, Ckb, Hsp90b1, Cyba, Mecom, Sept1, Nkx6-2, Gm48362, Cldn7, Crygs, Cd24a,
## Amot, Bend5, 1700007K13Rik, Cnn2, 3300005D01Rik, 1700097N02Rik, Gm10687, Tpo,
## Pfn1, Emp1, Cstb, Prrc1, Fam124a, Mcrip2, Srpk1, Slit2, Tbx3, Exoc1, Sapcd1,
## Gm49016, Lepr, Zfp560, Ghr, Prtg, Edn2, Dppa2, Upf1, Snhg18, Glyat, Fhl2, Hspb1,
## Plin2, Rhoc, Mt4, Tfpi, Pla2g1b, Pdia6, Fam151a, Srxn1, Fosl1, Sema3c, H2-
## M2, Fam162a, Ccl5, Wnt6, Gm48168, Rbp7, Peg10, Eva1a, Klhl13, Col27a1, Jund,
## Gm28578, Lgals9, Plet1, Tnc, Hes1, Ctsd, Dkkl1, Cdh11, Urah, 2310040G24Rik,
## Crlf1, B930036N10Rik, Bbx, Wnt7b, Cldn6, Ereg, Noct, 4933434E20Rik, Slc29a1,
## Folr1, 0610005C13Rik, Notch2, Loxl3, Fn1, Emb, Sag, Gjb5, Gpat3, Lgmn, Cabp1,
## Egr1, Pttg1, Fxyd5, Clu, Alcam, Anxa3, Nfam1, Timp3, Erf, Gapdh, Eprn, Prps1,
## Tcl1, Cdc20, Rasl11a, 1190005I06Rik, Sox9, Fosb, Pmaip1, Rrm2, Creb3l2, Hspb2,
## Epcam, Cebpd, Gm36266, Cebpb, Gbp2, Jam2, Jpt1, Arl4c, Pde6a, Irf7, Msmo1,
## Tnfrsf12a, H1fx, Spats2l, Tppp3, Nrn1, Ccnb1, Socs3, Slc27a2, Ccno, Lef1, Sox7,
## Dppa5a, Pdgfa, Sfrp1, Ifit3, Gm13964, Marcks, Nusap1, Asns, Flt1, Tfec, Ccnd3,
## Ldlr, Tdh, Foxp1, Lhfpl2, Cd9, Clic1, Hnf1b, Pcgf2, Zfp516, Cyp26a1, Phactr1,
## Gm2115, Ptp4a3, Ung, Reep5, Insig1, Prr13, Fstl1, Slc10a1, Rgs16, Mrpl9, Fmr1nb,
## Crct1, Klf5, Sycp1, Slc40a1, Atpaf2, Hist1h1b, Areg, Nnmt, Prc1, Hmces, Hmgcr,
## Mymx, Pdzd3, Pfas, Gm16222, Myef2, Lbh, Dnajb14, Fgf4, Plcxd3, Hmgn5, Ptpn22,
## Nkx6-3, Actg1, Alpk3, Wdr34, Clic5, Bhlhe40, Stard10, Vmn1r15, Btg1, Ndrg1,
## Glul, Map1b, Tpbgl, 4933408N05Rik, Col5a1, Ddah1, Ncam1, Ypel2, Gm9, Usp17lb,
## Tubb3, Rps15a, Myl4, Tagln2, Tuba1a, Pglyrp1, Bex1, Gpx8, Arl6ip1, Gm16755,
## Socs2, Slc39a4, Spry4, Herpud1, Slc48a1, Alkbh5, Ddit3, Nectin4, Crabp2, Gsta4,
## Rhox1, Kdm6b, Cenpa, Apbb1ip, Abhd2, Pitx2, Gast, Glrx, Ifit3b, Adam12, Tuba1c,
## Trib3, Snhg11, Cks2, Gm11232, Mbnl2, Utf1, Akr1c19, Dusp9, Calca, Sat1, Cmtm7,
## Gnas, Mc1r, Rab3il1, 3830417A13Rik, Kdelr3, Crip2, Scd2, Dnmt3b, Spink10,
## Fthl17c, Tuba3a, Dnajc3, Ehmt2, Thy1, Hsf2, Ptma, Smagp, Csrp1, Edn1, Rps12,
## Crim1, Stmn1, Rnf168, Hsd17b14, Cnr2, Gm16104, Ldoc1, Esd, Gm5463, Dap, Cxcl16,
## Apela, Gng12, Lamc1, Platr31, Platr5, Man2b1, Igfbp3, Calr, Bcl2l11, Adgrl3,
## H2-K1, P4ha1, Egln3, Gata2, Aurka, Ptgr1, Hmgb3, Slc38a2, Msx2, Ppia, Tspan1,
## Tapbpl, Pdcd4, Ankrd37, Mpped2, Gm2694, Foxd3, Epha4, Ube2s, Iqcb1, Oasl2,
## Fermt1, Arl4d, Dst, Gal, Sin3b, L1td1, Rpl22l1, Mdga2, Apoc1, Ctsz, Tmem30c,
## Pmepa1, Sms, Cotl1, Rps25, H2-M5, Klf3, Pcdh17, Hmgn3, Rap2b, Crb3, Vax2, Rbp1,
## Pgam2, Baz1a, Phldb2, Bbs2, Ppp1r11, Zfhx2, Eras, Mt3, Selenow, Amotl2, Basp1,
## Efna4, Serpinb6c, Ahnak, Ramp2, Capg, F2r, Tsc22d1, Gdpd5, Gadd45b, Bbc3, Pola2,
## Mgst3, Ptpre, Cda, Dstn, Slc38a4, Dhrs3, Uchl1, Dlk1, Mael, Patl2, Efna3, Mirg,
## Cenpe, Pmp22, Fgfr3, Ticrr, Mlana, Rpsa, Lhfp, Cpe, Cited1, Slc7a7, Calm1,
## Slc7a14, Pcbd1, Pou6f2, 9530053A07Rik, Gas6, Grasp, Elf3, Nrg1, Cdc42ep5,
## Mras, Phf21a, Nab2, Cltb, Sgk3, Foxc1, Gm26829, Hexa, Ucp2, Marcksl1, Taf7l,
## Pbx1, Tmem108, Icam1, Gm48170, Akap12, Cox4i2, Lockd, Ngfr, Cdc42ep3, Acot1,
## Sox15, Rsrp1, Sertad1, Fam213a, Srm, Dusp4, Fkbp6, Msln, Slc26a10, H2afj,
## Pcsk1n, Porcn, Cd59a, Serpinb9b, Gm29797, Wfdc15a, Scn1b, Kif21a, Tnfsf9, Cpn1,
## Bdh2, Ybx1, Fam181b, Tubb2a, Fxyd4, Rarg, Pcolce, Slc5a5, Mdfi, Tshz1, Laptm5,
## 4930461G14Rik, AI838599, Rpp25, Unc5b, Plod2, Isg20, Fzd7, Slc7a6, Cavin1, Nav2,
## Slc1a3, Ppdpf, Pitpnc1, Rasip1, Psmb10, Nrp2, Hkdc1, 2410137M14Rik, Dcbld1,
## Fosl2, Hoxb9, Cck, Lce1g, Gpnmb, Alppl2, Pf4, Gm8016, Lyz2, Mmp12, Tyrobp,
## Acta1, Ankrd1, Gm11361, Clec4d, Hspa1a, Hspa12b, Gm10696, Crabp1, Cwc22, Wfdc17,
## Ctss, Lonp2, Pgf, Mmp13, Nelfa, Slc25a4, Slc25a43, Arg1, Nppb, Slpi, Hspa8,
## Ctla2a, Fcgr3, Fcer1g, 1700012B09Rik, Cybb, Gm4858, Npy, Gm31805, Lce1h, C3ar1,
## Igfbp6, Hand1, Serpinf1, Cd36, Cxcl10, Ccl6, Rarres2, Rpl9-ps6, Fundc1, Mmp3,
## Clec4n, Npffr1, C5ar1, Saa3, Cxcl2, Ccl4, Stc1, Gkn1, Trpm1, Ndufa4l2, Gm13889,
## Cd52, Gkn2, 9130230L23Rik, Mgp, Csf2rb, Mmp10, Cd14, Akr1c18, Il1rn, Slc11a1,
## Adra2b, Dnmt3l, Bcl2a1b, Echdc3, Eif4a1, Slc2a1, Hsp90ab1, Car5b, Mmp9, Slc15a3,
## S100g, Dmkn, Nr2f1, Cxcl3, Lilr4b, Lncenc1, Ccl9, Cd53, Jsrp1, Nnat, Pdpn, Pclo,
## Gm10260, Ass1, Dcdc2c, Lce1f, Clec10a, Npm1, Olfr881, Serpine2, Sbsn, Mgll,
## Fxyd2, Olfr376, Thbd, Defb25, Gm32585, Lgals2, Gm33466, Psca, Slfn9, Bhlhe41,
## Igf1, Scx, Phlda3, Gm16638, Tex19.1, Txndc16, Wnt8a, Hist1h3c, Gm9112, Cct8,
## Pdzk1ip1, Spdye4b, Prdx1, Zfp352, Jph4, Pycr2, Cox6b2, Mpeg1, Usp18, Slc23a1,
## Olfr374, Eef1a1, Muc3, Ms4a7, Ccl8, Ifi27, Cthrc1, AL626769.1, Gm20234, C1qb,
## Dnajb9, Rrn3, Tubb4b, Prelp, 2010005H15Rik, Six3, Lcp1, Cd300ld, Pkm, Cs  
  ## Warning: Not all features provided are in this Assay object, removing the
## following feature(s): Gm5662, Pbld1, Srgn, Gm8300, Lefty1, H19, Gm5039,
## Olfr1369-ps1, Zscan4-ps2, Gm4027, Tmem80, Fry, Gm8994, Olfr889, Ptcra, Agl,
## Lgals1, Cyp4f14, Gm26870, Gm15879, Gm29666, Aldh1a3, Gm21761, Serpinh1,
## Zscan4c, Nlrc3, Zscan4d, Inhba, Fabp3, Gm29773, AF067061, Retn, Tagln, Noa1,
## BC147527, Gm45184, Gm8332, Foxq1, S100a4, Ctsl, Lhfpl4, Wdcp, Dab2, Plk4,
## Mcpt8, Gm43409, Slamf1, Gm4340, Acta2, Cryab, Meg3, Gm21762, Actb, S100a6,
## Gm2016, Alox8, Xist, Spp1, Krt18, Col4a1, Tcstv3, Spry2, Olfr1423, Zscan4-
## ps3, Rhox6, S100a3, Gm2022, Vim, Lefty2, Cdkn1c, Gm26737, Rrm2b, Spata31d1a,
## Tmsb4x, Xaf1, BC080695, Gata6, Usp17lc, AC160336.1, Thbs1, Gm45509, Bgn,
## Igfbp7, Gfod2, 4930548H24Rik, Amn, Col4a2, Krt8, Aqp8, Timp2, Id3, Hmga2,
## Mt1, Gm428, Tulp3, Ccnd2, Trh, Sparc, Grem1, Atg2a, A330069E16Rik, Dqx1, P3h3,
## Id1, Lama1, Aebp1, Glipr2, Hist1h2bc, Lgals4, Gm13075, Spata7, P2rx4, Rhox5,
## Unc13d, A930014E10Rik, Fst, 1010001N08Rik, AC163720.3, Sox17, Tc2n, Alpk2, Mt2,
## Igfbp4, Fbln2, Dkk1, Gm26909, Sp110, Twist2, Gm48754, Lrpap1, Cavin2, Rpl29,
## 6330410L21Rik, Fgfbp1, Apoe, Tm4sf1, Stmn2, Sfn, Gpx3, Usp17la, U90926, Gm12794,
## Pim1, A330032B11Rik, Gm10457, Fam25c, Lgals3, Gm13119, Krt7, Fbp2, Lamb1,
## Ifitm1, Gng11, Tpm2, Gata4, Tpm1, Calml4, Cyr61, 9330136K24Rik, Ccser1, Prex2,
## Bc1, Gm26710, Ccl7, Ifitm3, Fgf1, Cited4, Gm47510, Slc7a11, Csf1, P4ha2, Inpp4b,
## Col1a1, Dkk2, Comt, Limch1, Zscan4-ps1, Hspa5, Krtdap, Lgi1, Dennd5b, Serpine1,
## Hbegf, Lox, Oaf, AC125149.3, Ddit4, Gm48799, Cald1, Gm26782, Id2, Mylpf, Tpm4,
## Gm48610, 5430402O13Rik, Sdc4, Amt, Hmox1, AU019990, Serpinb2, Ngb, Gm13078,
## Scamp1, Nupr1, Siglec1, Ppbp, Fxyd3, Gm11238, Sp140, Calcoco2, Rpl39l, Cbx3,
## AC140186.1, Lmx1a, Neat1, Filip1l, C730034F03Rik, Usp26, Il11, Pth1r, Pou3f1,
## S100a13, Gm48764, Cd63, Prrx1, Ptgs2, Gm38947, Ascl2, Slc39a1, Gm26772, Chpt1,
## Gm26764, Sct, Ccl20, Hist1h2ap, Nfkbia, Pdgfrl, Phlda1, A530040E14Rik, Ccl3,
## B2m, Lrp2, Ctgf, Shkbp1, Hs3st1, Ier3, Car2, Gm8953, Gadd45g, Bex6, Cxcl1, Gsn,
## Ercc4, Gm20625, H2-T23, Dlg1, Cubn, Sox4, 4933440M02Rik, Gm43263, BC028528,
## Krt17, Terf2ip, Myl9, Tmem252, Gm16368, Ngf, Runx1, Enah, Fth1, Klf2, Ccl2,
## Tmem92, Dppa3, Cd44, Ube2c, Adcy2, Epop, Ncoa3, H1f0, Rps24, Gpx2, Gjb3, Txnip,
## Hsp90aa1, Cited2, Parvb, Fam46a, Cbr3, Rab33b, Avpi1, B020031M17Rik, Igfbp2,
## Mafb, Glrx2, Phkg1, Fos, Spink1, S100a11, 1810062G17Rik, Phlda2, Serpinb5,
## Gm2056, Dpys, Rep15, Lrrc32, Plaur, Bbs4, Gm21731, Dusp1, Il1rl1, Gm12280,
## Gpx4, Cav1, Tfap2c, Gabarapl2, Stra8, Oasl1, Gsto1, Klf4, Slc15a1, Ccdc80,
## Gm10827, Ubtfl1, Ifit1, Crip1, Jun, Gmcl1, Aqp3, Junb, Arhgef26, Marveld1,
## Krt19, Krt42, Anxa2, Timp1, Gm35339, Psmb8, Rnf128, Col5a2, Axl, 0610040J01Rik,
## Aard, Chrm3, Sgk1, 2200002D01Rik, Anxa1, Nodal, Rhox9, Gm20442, Pyy, AC191865.2,
## Gm46332, Itm2b, Htra1, G0s2, Myo1f, Cidea, Zfp296, Cenpf, Fgf3, Atf4, Col1a2,
## Adm, Bmp4, Sfmbt2, Ifi27l2a, Gm28940, H2-D1, Prrx2, Rasgrp2, Fbxo15, Gm12800,
## Cdkn2a, Tnfsf12, Gm21269, Pirb, Zar1, Tinagl1, Cxcl5, Gbp2b, Platr3, Scaf11,
## Arg2, Car4, Exosc2, Msc, Chac1, Tmem176b, Cst3, Rhox13, Gm15627, Isg15, Plk3,
## AC158554.1, Pdgfra, AC168977.1, Podxl, 9530059O14Rik, Irgm1, Dazl, Gm16233,
## Perp, H2afx, Zfp809, Gm5788, Slc7a3, Bnip3, Apobec2, Slc28a3, Skil, Ahnak2,
## Foxo3, Ly6a, Zfp428, Clmn, Slc24a5, Ccnd1, Plac8, Nefl, Pla2g12b, Arhgdib,
## Upp1, Anxa5, Gbx2, Drr1, Wtap, Khdc3, Malat1, 2610528J11Rik, 1600010M07Rik,
## Endov, Ccdc163, Foxa2, Klf6, Mycn, Nr2f2, Hook2, Utp11, Dok2, Ubald2, Wnt4,
## Wfdc2, Fxyd6, Cdx2, Fam186b, Fabp5, Gadd45a, S100a10, Flrt3, Nat8f2, Ooep,
## Ebf1, Ckb, Hsp90b1, Cyba, Mecom, Sept1, Nkx6-2, Gm48362, Cldn7, Crygs, Cd24a,
## Amot, Bend5, 1700007K13Rik, Cnn2, 3300005D01Rik, 1700097N02Rik, Gm10687, Tpo,
## Pfn1, Emp1, Cstb, Prrc1, Fam124a, Mcrip2, Srpk1, Slit2, Tbx3, Exoc1, Sapcd1,
## Gm49016, Lepr, Zfp560, Ghr, Prtg, Edn2, Dppa2, Upf1, Snhg18, Glyat, Fhl2, Hspb1,
## Plin2, Rhoc, Mt4, Tfpi, Pla2g1b, Pdia6, Fam151a, Srxn1, Fosl1, Sema3c, H2-
## M2, Fam162a, Ccl5, Wnt6, Gm48168, Rbp7, Peg10, Eva1a, Klhl13, Col27a1, Jund,
## Gm28578, Lgals9, Plet1, Tnc, Hes1, Ctsd, Dkkl1, Cdh11, Urah, 2310040G24Rik,
## Crlf1, B930036N10Rik, Bbx, Wnt7b, Cldn6, Ereg, Noct, 4933434E20Rik, Slc29a1,
## Folr1, 0610005C13Rik, Notch2, Loxl3, Fn1, Emb, Sag, Gjb5, Gpat3, Lgmn, Cabp1,
## Egr1, Pttg1, Fxyd5, Clu, Alcam, Anxa3, Nfam1, Timp3, Erf, Gapdh, Eprn, Prps1,
## Tcl1, Cdc20, Rasl11a, 1190005I06Rik, Sox9, Fosb, Pmaip1, Rrm2, Creb3l2, Hspb2,
## Epcam, Cebpd, Gm36266, Cebpb, Gbp2, Jam2, Jpt1, Arl4c, Pde6a, Irf7, Msmo1,
## Tnfrsf12a, H1fx, Spats2l, Tppp3, Nrn1, Ccnb1, Socs3, Slc27a2, Ccno, Lef1, Sox7,
## Dppa5a, Pdgfa, Sfrp1, Ifit3, Gm13964, Marcks, Nusap1, Asns, Flt1, Tfec, Ccnd3,
## Ldlr, Tdh, Foxp1, Lhfpl2, Cd9, Clic1, Hnf1b, Pcgf2, Zfp516, Cyp26a1, Phactr1,
## Gm2115, Ptp4a3, Ung, Reep5, Insig1, Prr13, Fstl1, Slc10a1, Rgs16, Mrpl9, Fmr1nb,
## Crct1, Klf5, Sycp1, Slc40a1, Atpaf2, Hist1h1b, Areg, Nnmt, Prc1, Hmces, Hmgcr,
## Mymx, Pdzd3, Pfas, Gm16222, Myef2, Lbh, Dnajb14, Fgf4, Plcxd3, Hmgn5, Ptpn22,
## Nkx6-3, Actg1, Alpk3, Wdr34, Clic5, Bhlhe40, Stard10, Vmn1r15, Btg1, Ndrg1,
## Glul, Map1b, Tpbgl, 4933408N05Rik, Col5a1, Ddah1, Ncam1, Ypel2, Gm9, Usp17lb,
## Tubb3, Rps15a, Myl4, Tagln2, Tuba1a, Pglyrp1, Bex1, Gpx8, Arl6ip1, Gm16755,
## Socs2, Slc39a4, Spry4, Herpud1, Slc48a1, Alkbh5, Ddit3, Nectin4, Crabp2, Gsta4,
## Rhox1, Kdm6b, Cenpa, Apbb1ip, Abhd2, Pitx2, Gast, Glrx, Ifit3b, Adam12, Tuba1c,
## Trib3, Snhg11, Cks2, Gm11232, Mbnl2, Utf1, Akr1c19, Dusp9, Calca, Sat1, Cmtm7,
## Gnas, Mc1r, Rab3il1, 3830417A13Rik, Kdelr3, Crip2, Scd2, Dnmt3b, Spink10,
## Fthl17c, Tuba3a, Dnajc3, Ehmt2, Thy1, Hsf2, Ptma, Smagp, Csrp1, Edn1, Rps12,
## Crim1, Stmn1, Rnf168, Hsd17b14, Cnr2, Gm16104, Ldoc1, Esd, Gm5463, Dap, Cxcl16,
## Apela, Gng12, Lamc1, Platr31, Platr5, Man2b1, Igfbp3, Calr, Bcl2l11, Adgrl3,
## H2-K1, P4ha1, Egln3, Gata2, Aurka, Ptgr1, Hmgb3, Slc38a2, Msx2, Ppia, Tspan1,
## Tapbpl, Pdcd4, Ankrd37, Mpped2, Gm2694, Foxd3, Epha4, Ube2s, Iqcb1, Oasl2,
## Fermt1, Arl4d, Dst, Gal, Sin3b, L1td1, Rpl22l1, Mdga2, Apoc1, Ctsz, Tmem30c,
## Pmepa1, Sms, Cotl1, Rps25, H2-M5, Klf3, Pcdh17, Hmgn3, Rap2b, Crb3, Vax2, Rbp1,
## Pgam2, Baz1a, Phldb2, Bbs2, Ppp1r11, Zfhx2, Eras, Mt3, Selenow, Amotl2, Basp1,
## Efna4, Serpinb6c, Ahnak, Ramp2, Capg, F2r, Tsc22d1, Gdpd5, Gadd45b, Bbc3, Pola2,
## Mgst3, Ptpre, Cda, Dstn, Slc38a4, Dhrs3, Uchl1, Dlk1, Mael, Patl2, Efna3, Mirg,
## Cenpe, Pmp22, Fgfr3, Ticrr, Mlana, Rpsa, Lhfp, Cpe, Cited1, Slc7a7, Calm1,
## Slc7a14, Pcbd1, Pou6f2, 9530053A07Rik, Gas6, Grasp, Elf3, Nrg1, Cdc42ep5,
## Mras, Phf21a, Nab2, Cltb, Sgk3, Foxc1, Gm26829, Hexa, Ucp2, Marcksl1, Taf7l,
## Pbx1, Tmem108, Icam1, Gm48170, Akap12, Cox4i2, Lockd, Ngfr, Cdc42ep3, Acot1,
## Sox15, Rsrp1, Sertad1, Fam213a, Srm, Dusp4, Fkbp6, Msln, Slc26a10, H2afj,
## Pcsk1n, Porcn, Cd59a, Serpinb9b, Gm29797, Wfdc15a, Scn1b, Kif21a, Tnfsf9, Cpn1,
## Bdh2, Ybx1, Fam181b, Tubb2a, Fxyd4, Rarg, Pcolce, Slc5a5, Mdfi, Tshz1, Laptm5,
## 4930461G14Rik, AI838599, Rpp25, Unc5b, Plod2, Isg20, Fzd7, Slc7a6, Cavin1, Nav2,
## Slc1a3, Ppdpf, Pitpnc1, Rasip1, Psmb10, Nrp2, Hkdc1, 2410137M14Rik, Dcbld1,
## Fosl2, Hoxb9, Cck, Lce1g, Gpnmb, Alppl2, Pf4, Gm8016, Lyz2, Mmp12, Tyrobp,
## Acta1, Ankrd1, Gm11361, Clec4d, Hspa1a, Hspa12b, Gm10696, Crabp1, Cwc22, Wfdc17,
## Ctss, Lonp2, Pgf, Mmp13, Nelfa, Slc25a4, Slc25a43, Arg1, Nppb, Slpi, Hspa8,
## Ctla2a, Fcgr3, Fcer1g, 1700012B09Rik, Cybb, Gm4858, Npy, Gm31805, Lce1h, C3ar1,
## Igfbp6, Hand1, Serpinf1, Cd36, Cxcl10, Ccl6, Rarres2, Rpl9-ps6, Fundc1, Mmp3,
## Clec4n, Npffr1, C5ar1, Saa3, Cxcl2, Ccl4, Stc1, Gkn1, Trpm1, Ndufa4l2, Gm13889,
## Cd52, Gkn2, 9130230L23Rik, Mgp, Csf2rb, Mmp10, Cd14, Akr1c18, Il1rn, Slc11a1,
## Adra2b, Dnmt3l, Bcl2a1b, Echdc3, Eif4a1, Slc2a1, Hsp90ab1, Car5b, Mmp9, Slc15a3,
## S100g, Dmkn, Nr2f1, Cxcl3, Lilr4b, Lncenc1, Ccl9, Cd53, Jsrp1, Nnat, Pdpn, Pclo,
## Gm10260, Ass1, Dcdc2c, Lce1f, Clec10a, Npm1, Olfr881, Serpine2, Sbsn, Mgll,
## Fxyd2, Olfr376, Thbd, Defb25, Gm32585, Lgals2, Gm33466, Psca, Slfn9, Bhlhe41,
## Igf1, Scx, Phlda3, Gm16638, Tex19.1, Txndc16, Wnt8a, Hist1h3c, Gm9112, Cct8,
## Pdzk1ip1, Spdye4b, Prdx1, Zfp352, Jph4, Pycr2, Cox6b2, Mpeg1, Usp18, Slc23a1,
## Olfr374, Eef1a1, Muc3, Ms4a7, Ccl8, Ifi27, Cthrc1, AL626769.1, Gm20234, C1qb,
## Dnajb9, Rrn3, Tubb4b, Prelp, 2010005H15Rik, Six3, Lcp1, Cd300ld, Pkm, Cs  
  # subsetting and assign levels
ESCTBLC1.combined<-subset(x = ESCTBLC1.combined, idents = c('2CLC','ESCs','TBLCs'))
ClusESCTBLC1.combined_levels <- c('2CLC','ESCs','TBLCs')
levels(ESCTBLC1.combined) <- ClusESCTBLC1.combined_levels  
  #Repeat result from Figure S3H

# Pluripotent genes
VlnPlot(ESCTBLC1.combined, features = 
          c("Nr0b1","Sox2","Nanog","Tdgf1","H2afz","Tubb5")
        ,ident=c('2CLC','ESCs', 'TBLCs'),assay='integrated',pt.size=0)  
   
  VlnPlot(ESCTBLC1.combined, features = 
          c("Pou5f1","Nop10","Zfp42","Tcf15","Tet1","Esrrb")
        ,ident=c('2CLC','ESCs', 'TBLCs'),assay='integrated',pt.size=0)  
   
  # Totipotent genes
VlnPlot(ESCTBLC1.combined, features = 
          c("Plk2","Cdkn1a","Zfp365","Trp53inp1","Mdm2","Btg2")
        ,ident=c('2CLC','ESCs', 'TBLCs'),assay='integrated',pt.size=0)  
   
  VlnPlot(ESCTBLC1.combined, features = 
          c("Ddit4l","Pdrg1","Pid1","Emp3","Dffb","Ctsb")
        ,ident=c('2CLC','ESCs', 'TBLCs'),assay='integrated',pt.size=0)  
   
  # cluster TBLC at a greater resolution
TBLC <- FindClusters(TBLC, resolution = 0.5)  
  ## Modularity Optimizer version 1.3.0 by Ludo Waltman and Nees Jan van Eck
## 
## Number of nodes: 4534
## Number of edges: 152115
## 
## Running Louvain algorithm...
## Maximum modularity in 10 random starts: 0.8532
## Number of communities: 8
## Elapsed time: 0 seconds  
  # Run non-linear dimentional reduction (UMAP/tSNE)
# If you haven't installed UMAP, you can do so via reticulate::py_install(packages =
# 'umap-learn')
TBLC <- RunUMAP(TBLC, dims = 1:15)  
  ## 15:58:44 UMAP embedding parameters a = 0.9922 b = 1.112  
  ## 15:58:44 Read 4534 rows and found 15 numeric columns  
  ## 15:58:44 Using Annoy for neighbor search, n_neighbors = 30  
  ## 15:58:44 Building Annoy index with metric = cosine, n_trees = 50  
  ## 0%   10   20   30   40   50   60   70   80   90   100%  
  ## [----|----|----|----|----|----|----|----|----|----|  
  ## **************************************************|
## 15:58:44 Writing NN index file to temp file /var/folders/cv/cfhg7t_j5y3d34y2_81gskv00000gn/T//RtmpdT6yOL/file4d316becee24
## 15:58:44 Searching Annoy index using 1 thread, search_k = 3000
## 15:58:46 Annoy recall = 100%
## 15:58:46 Commencing smooth kNN distance calibration using 1 thread
## 15:58:46 Initializing from normalized Laplacian + noise
## 15:58:46 Commencing optimization for 500 epochs, with 186234 positive edges
## 15:58:53 Optimization finished  
  # Assigning cluster names for future reference and data integration
TBLCs.clustered.ids <- c("0","1","3","2","4","MEF","5","6")
names(TBLCs.clustered.ids) <- levels(TBLC)
TBLC <- RenameIdents(TBLC, TBLCs.clustered.ids)

# merging TBLC, early development and ESC data
TBLCESC<-merge(TBLC,ESC)  
  ## Warning in CheckDuplicateCellNames(object.list = objects): Some cell names are
## duplicated across objects provided. Renaming to enforce unique cell names.  
  TBLCESCEarlyDeve<-merge(TBLCESC, EarlyDeve)

# splitting object identify for downstream cluster analysis
TBLCESCEarlyDeve1 <- SplitObject(TBLCESCEarlyDeve, split.by = "orig.ident")
TBLCESCEarlyDeve1  
  ## $TBLC
## An object of class Seurat 
## 22603 features across 4534 samples within 1 assay 
## Active assay: RNA (22603 features, 0 variable features)
## 
## $ESC
## An object of class Seurat 
## 22603 features across 4139 samples within 1 assay 
## Active assay: RNA (22603 features, 0 variable features)
## 
## $EarlyDevelopment
## An object of class Seurat 
## 22603 features across 259 samples within 1 assay 
## Active assay: RNA (22603 features, 0 variable features)  
  # normalize and identify variable features for each dataset independently
TBLCESCEarlyDeve1 <- lapply(X = TBLCESCEarlyDeve1, FUN = function(x) {
    x <- NormalizeData(x)
    x <- FindVariableFeatures(x, selection.method = "vst")
})

# integrating data by integrating 2 seurat objects as input
TBLCESCEarlyDeve1.anchors <- FindIntegrationAnchors(object.list = TBLCESCEarlyDeve1, dims = 1:20)  
  ## Computing 2000 integration features
## Scaling features for provided objects
## Finding all pairwise anchors
## Running CCA
## Merging objects
## Finding neighborhoods
## Finding anchors
##  Found 12297 anchors
## Filtering anchors
##  Retained 3968 anchors
## Running CCA
## Merging objects
## Finding neighborhoods
## Finding anchors
##  Found 1242 anchors
## Filtering anchors
##  Retained 1060 anchors
## Running CCA
## Merging objects
## Finding neighborhoods
## Finding anchors
##  Found 1219 anchors
## Filtering anchors
##  Retained 1029 anchors  
  TBLCESCEarlyDeve1.combined <- IntegrateData(anchorset = TBLCESCEarlyDeve1.anchors, dims = 1:20,features.to.integrate = c("Rest","Utf1","Tcf15","H2afx","Aes","Nr0b1","Sox2","Nanog","Tdgf1","H2afz","Tubb5","Pou5f1","Nop10","Zfp42","Tcf15","Tet1","Esrrb","Plk2","Cdkn1a","Zfp365","Trp53inp1","Mdm2","Btg2","Ddit4l","Pdrg1","Pid1","Emp3","Dffb","Ctsb","Ctsd","Cd81","Mt1","Gm5662","Gm8300"),verbose=FALSE)  
  ## Warning: Not all features provided are in this Assay object, removing the
## following feature(s): Pbld1, Srgn, Lefty1, H19, Olfr1369-ps1, Zscan4-ps2,
## Gm4027, Tmem80, Fry, Gm8994, Olfr889, Ptcra, Agl, Cyp4f14, Gm26870, Gm15879,
## Gm29666, Aldh1a3, Gm21761, Gm5039, Zscan4c, Nlrc3, Lgals1, Inhba, Gm29773,
## AF067061, Retn, Tagln, Noa1, BC147527, Gm45184, Gm8332, Foxq1, Zscan4d, S100a4,
## Fabp3, Lhfpl4, Wdcp, Plk4, Serpinh1, Mcpt8, Gm43409, Slamf1, Acta2, Cryab,
## Gm21762, S100a6, Alox8, Col4a1, Tcstv3, Spry2, Olfr1423, Zscan4-ps3, Ctsl,
## Rhox6, S100a3, Gm4340, Spp1, Vim, Lefty2, Cdkn1c, Gm26737, Spata31d1a, Xaf1,
## Dab2, Gata6, Usp17lc, BC080695, Rrm2b, AC160336.1, Thbs1, Gm45509, Bgn, Igfbp7,
## Meg3, Gfod2, Gm2016, 4930548H24Rik, Amn, Col4a2, Timp2, Tmsb4x, Aqp8, Id3,
## Hmga2, Gm2022, Ccnd2, Trh, Grem1, Atg2a, A330069E16Rik, Dqx1, P3h3, Id1, Gm428,
## Xist, Lama1, Aebp1, Hist1h2bc, Lgals4, Gm13075, Spata7, P2rx4, Rhox5, Unc13d,
## Actb, Krt18, A930014E10Rik, Fst, 1010001N08Rik, AC163720.3, Sox17, Tc2n, Alpk2,
## Mt2, Igfbp4, Txnip, Zfp352, Fbln2, Dkk1, Gm26909, Twist2, Gm48754, Lrpap1,
## Cavin2, Rpl29, 6330410L21Rik, Krt8, Fgfbp1, Apoe, Tm4sf1, Stmn2, Sfn, Gpx3,
## Usp17la, U90926, A330032B11Rik, Gm10457, Fam25c, Lgals3, Krt7, Fbp2, Lamb1,
## Ifitm1, Gng11, Tpm2, Gm13119, Tpm1, Calml4, Cyr61, 9330136K24Rik, Ccser1, Sparc,
## Prex2, Bc1, Gm26710, Ccl7, Ifitm3, Fgf1, Cited4, Gm47510, Tulp3, Csf1, P4ha2,
## Inpp4b, Col1a1, Rgs2, Obox3, Dkk2, Comt, Fcgr3, Zscan4-ps1, Hspa5, Slc16a6,
## Pim1, Krtdap, Lgi1, Dennd5b, Serpine1, Hbegf, Lox, Oaf, AC125149.3, Ddit4,
## Gm48799, Cald1, Glipr2, Gm26782, Mylpf, Tpm4, Gm48610, Limch1, 1600025M17Rik,
## 5430402O13Rik, Gm13078, Slc7a11, Sdc4, Amt, Hmox1, AU019990, Serpinb2, Ngb,
## Ubtfl1, Foxr1, Nupr1, Siglec1, Ppbp, Pyy, Fxyd3, Gm11238, Sp140, Rimklb,
## Calcoco2, Rfpl4b, Rpl39l, Cbx3, AC140186.1, Neat1, Ccno, Filip1l, B020004J07Rik,
## Dnajb9, Lmx1a, C730034F03Rik, Ccne1, Il11, Pth1r, Pou3f1, S100a13, Dcdc2c,
## Gm48764, Cd63, Gm12794, Prrx1, Pld1, Ptgs2, Gm38947, Ascl2, Slc39a1, Gm26772,
## Chpt1, Gm26764, Sct, Ccl20, Hist1h2ap, Nfkbia, Pdgfrl, Phlda1, A530040E14Rik,
## Ccl3, B2m, Ctgf, Sp110, Shkbp1, Hs3st1, Ier3, Car2, Gm8953, Gadd45g, Bex6,
## Cxcl1, Gsn, Gm11487, Ercc4, Gm20625, H2-T23, Zfp57, Dlg1, Cubn, Atf2, Usp26,
## 4933440M02Rik, Gm43263, Dnajb14, BC028528, Gata4, Krt17, Terf2ip, Myl9, Fam46b,
## Alppl2, Tmem252, Gm16368, Ngf, Runx1, Enah, Fth1, Klf2, Ccl2, Tmem92, Dppa3,
## Zar1, Cd44, Ldhb, Ube2c, Nexn, Adcy2, Epop, 1700013H16Rik, Rasl11a, Id2, Ncoa3,
## H1f0, Rps24, Gpx2, Gjb3, Hsp90aa1, Cited2, Parvb, Fam46a, Plk3, Cbr3, Rab33b,
## Sox4, Avpi1, B020031M17Rik, Igfbp2, Mafb, Phkg1, Scamp1, Fos, Spink1, S100a11,
## Dazl, 1810062G17Rik, Phlda2, Plac8, Serpinb5, Pttg1, Gm2056, Dpys, Rep15,
## Gm10696, Lrrc32, Npm2, Plaur, Gm21731, Gm7102, Il1rl1, Gm12280, Gpx4, Cav1,
## Tfap2c, Gabarapl2, Stra8, Oasl1, Gsto1, Klf4, Slc15a1, Ccdc80, Gm10827, Lcp1,
## Ifit1, Crip1, Jun, Gmcl1, Aqp3, Junb, Arhgef26, A530032D15Rik, Marveld1, Krt19,
## Jag1, Krt42, Anxa2, Snai1, Tacr3, Tiparp, Timp1, Gm35339, Psmb8, Rnf128, Ctss,
## Hspa1a, Col5a2, Axl, Ooep, Aard, AU022751, Chrm3, Ndrg1, Slc39a10, Gadd45a,
## Lrp2, Pank3, Sgk1, Slc34a2, 2200002D01Rik, Anxa1, Nodal, Rhox9, Gm20442,
## 0610040J01Rik, AC191865.2, Gm46332, Itm2b, Htra1, G0s2, Tcl1, Myo1f, Cidea,
## Arg2, Fgf3, Atf4, Smagp, Col1a2, Adm, Itpr2, Sfmbt2, Spry4, Ifi27l2a, Csrnp3,
## Gm28940, H2-D1, Rasgrp2, Fbxo15, Gm12800, Insm1, Cdkn2a, Tnfsf12, Dusp1,
## Gm21269, Pirb, Olfr881, Tinagl1, Cxcl5, Glrx2, Hmgb3, Gbp2b, Platr3, Glul,
## Gm9125, Car4, Cpsf4l, Spz1, Exosc2, Msc, Chac1, Tmem176b, Zfp296, Cst3, Rhox13,
## Mbd5, Gm15627, Isg15, Cd47, AC158554.1, AC168977.1, Podxl, 9530059O14Rik, Irgm1,
## Tdpoz1, Pnp, Gm16233, Perp, Zfp809, Gm5788, Bnip3, Apobec2, Eda, Rbm15, Slc28a3,
## Olfr376, Skil, Ahnak2, Oas1e, Ly6a, Zfp428, Clmn, Slc24a5, Ccnd1, Ppp1r15a,
## Nefl, Pla2g12b, Arhgdib, Anxa5, Gbx2, Drr1, Khdc3, Sesn2, Malat1, 2610528J11Rik,
## Tcl1b2, 1600010M07Rik, Endov, Ccdc163, Foxa2, Klf6, Mycn, Socs3, Nr2f2, Hook2,
## Utp11, Dok2, Ubald2, Wnt4, Wfdc2, Wtap, Fxyd6, Cdx2, Fam186b, Fabp5, S100a10,
## Nat8f2, Ebf1, Ckb, Hsp90b1, Ythdf2, Antxr1, Cyba, Tdrd12, Tesc, Bbs4, Hmgn5,
## Lmo7, Mecom, Sept1, Nkx6-2, Zfp622, Gm48362, Cldn7, Crygs, Cenpf, Cd24a,
## Rbbp6, Amot, Bspry, Bend5, 1700007K13Rik, G2e3, Errfi1, Cnn2, 3300005D01Rik,
## 1700097N02Rik, Obox6, Tspan8, Tubb3, Gm10687, Pfn1, Emp1, Cstb, Prrc1, Fam124a,
## Mcrip2, Srpk1, Slit2, Cdc20, Pdgfra, Tbx3, Exoc1, Sapcd1, Gm49016, Zfp560, Ghr,
## Prtg, Edn2, Dppa2, Upf1, Snhg18, Glyat, Mad2l1bp, Fhl2, Hspb1, Zfand5, Il1rn,
## Slc7a3, Eif4e1b, Kdm5b, Plin2, Rhoc, Mt4, Pla2g1b, Mthfd2, Pdia6, Cst7, Bmp4,
## Fam151a, Srxn1, Fosl1, Arl4d, Sema3c, Ninj1, H2-M2, Mphosph6, Fam162a, Tsc22d1,
## Ccl5, Fam109a, Wnt6, Gm48168, Rbp7, Nrp1, Fundc1, Peg10, Pnrc1, Gm4858, Spesp1,
## Eva1a, Klf17, Klhl13, Col27a1, Jund, Gm28578, Plet1, Prrx2, Tnc, Hes1, Scaf11,
## Dkkl1, Il6ra, Rnf11, Cdh11, Urah, 2310040G24Rik, Crlf1, B930036N10Rik, Bbx,
## Wnt7b, Foxo3, Cldn6, Ereg, Gng12, Noct, 4933434E20Rik, Pde6a, Fam110c, Slc29a1,
## Cab39, Parp12, Ttc30b, Dpysl3, Folr1, Notch2, Flrt3, Loxl3, Fn1, Emb, Sag, Gjb5,
## Stk35, Gpat3, Fam83d, Lgmn, Cabp1, Rrn3, Dynll2, Egr1, Abhd6, Tmprss2, Fxyd5,
## Clu, Alcam, Anxa3, Fdft1, Klk7, Adra2b, Nfam1, Timp3, Erf, Gapdh, Eprn, Slco3a1,
## Cpeb1, Znhit3, 1190005I06Rik, Sox9, Fosb, Pmaip1, Slbp, Smad7, Rrm2, Creb3l2,
## Hspb2, Dppa1, Epcam, Muc13, Cebpd, Taok3, Gm36266, Cebpb, Gbp2, Jam2, Jpt1,
## Arl4c, Paip2b, Pim2, Irf7, Msmo1, Npy, H1fx, Spats2l, Tppp3, Kctd3, Nrn1, Ccnb1,
## Dcc, Slc27a2, AU015836, Lef1, Sox7, Dppa5a, Pdgfa, Relb, Sfrp1, Ifit3, Gm13964,
## Jam3, Marcks, Nusap1, Flt1, Tfec, Ccnd3, Ldlr, Tdh, Foxp1, Lhfpl2, Dis3l, Cd9,
## Fgf7, Clic1, Pcgf2, Akr1c21, Zfp516, Cyp26a1, Phactr1, Anks4b, Ctnnal1, Gm2115,
## Lsp1, Ptp4a3, Ung, Reep5, Gata1, Insig1, Prr13, Fstl1, Slc10a1, Rgs16, Lgals9,
## Mrpl9, Fmr1nb, Crct1, Klf5, Slc40a1, Bfsp1, Mdm4, Atpaf2, Lepr, Nup98, Hist1h1b,
## Areg, Nnmt, Gm5, Hmces, Hmgcr, Mymx, Crabp2, Pdzd3, Pfas, Gm16222, Myef2,
## Gadd45b, Alkbh5, Fgf4, Lrrc8c, Plcxd3, Pim3, Ptpn22, Nkx6-3, Actg1, Alpk3,
## Wdr34, Clic5, Bhlhe40, Stard10, Vmn1r15, Btg1, Tubb6, 4930550L24Rik, Map1b,
## Tpbgl, Ddr2, 4933408N05Rik, Col5a1, Ddah1, Ier5, Ncam1, Fbxo34, Gm9, Usp17lb,
## Rps15a, Myl4, Tagln2, Tuba1a, Pglyrp1, Bex1, Gpx8, Arap2, Arl6ip1, Gm16755,
## Socs2, Slc39a4, Herpud1, Slc48a1, Ddit3, 2810474O19Rik, Nectin4, Baz1a, Gsta4,
## Rhox1, Tmem37, Kdm6b, 0610005C13Rik, Zc3h6, Cenpa, Apbb1ip, Abhd2, Tfpi, Pitx2,
## Usp18, Gast, Ctf2, Glrx, Ifit3b, Ppig, Adam12, Brd2, Tuba1c, Tob1, Snhg11, Cks2,
## Gm11232, Mbnl2, Cnr2, Akr1c19, Gabra1, Dusp9, Upp1, Calca, Sat1, Bhlha15, Cmtm7,
## Atrx, Gnas, Mc1r, Rab3il1, Cxcl16, 3830417A13Rik, Kdelr3, Crip2, Scd2, Dnmt3b,
## Maml3, Serpina3g, Spink10, Fthl17c, Tuba3a, Dnajc3, Ehmt2, Thy1, Hsf2, Ptma,
## Tpo, Soat1, Csrp1, Prc1, Edn1, Ptpre, Nasp, Zp3, Rps12, Mpzl1, Cltb, Crim1,
## Stmn1, Rnf168, Unc5c, Trib3, Incenp, Icam1, Gm16104, Psap, Ldoc1, Esd, Gm5463,
## Dnajc21, Nub1, Dap, Spic, Apela, Uchl1, Lamc1, Elf1, Platr31, Platr5, Man2b1,
## Igfbp3, Pip5k1a, Sh3kbp1, Calr, Tmcc3, Bcl2l11, Nav1, Adgrl3, Trpm6, Sdc1,
## Ampd3, H2-K1, P4ha1, Egln3, Gata2, Pcgf5, Aurka, Ptgr1, Asns, Tacstd2, Msx2,
## Ppia, Tspan1, Nr5a2, Tapbpl, Pdcd4, Sertad1, Ankrd37, Sf3b1, Mpped2, Gm2694,
## Foxd3, Epha4, Ube2s, Iqcb1, Oasl2, Fermt1, Dst, Gal, Pnma5, Mast4, Sin3b, L1td1,
## Rpl22l1, Tgfb3, Mdga2, Apoc1, Ctsz, Tmem30c, Slc38a2, Pmepa1, Tnfrsf12a, Sms,
## Cotl1, Xpnpep2, Rps25, H2-M5, Klf3, Pcdh17, Hmgn3, Rap2b, Crb3, Vax2, Rbp1,
## Pgam2, Ypel2, Synm, Ceacam1, Phldb2, Bbs2, Dyrk3, Ppp1r11, Zfhx2, Eras, Irf1,
## Mt3, Selenow, Amotl2, Nptx2, Basp1, Efna4, Slc2a3, Serpinb6c, Ramp2, F2r, Esrp1,
## Kif20b, Lysmd1, Gdpd5, Bbc3, Pola2, Pdzd2, Sh3bgrl, Cda, Arhgap25, Dstn, Galnt6,
## Slc38a4, Dlk1, Mael, Patl2, Efna3, Lpar6, Mirg, Ahnak, Aass, Cenpe, Pmp22,
## Fgfr3, Sycp1, Hsd17b14, Fam46c, Car7, Ticrr, Mlana, Sqle, Rpsa, Lhfp, Ovol2,
## Cpe, Elavl3, Lat2, Zfp553, Cited1, Slc7a7, Msn, Calm1, Slc7a14, Pcbd1, Zbtb10,
## Pou6f2, Manba, 9530053A07Rik, Gas6, Grasp, Elf3, Nrg1, Abtb2, C8b, Dcaf12l1,
## Nefh, Chd7, Cdc42ep5, Eomes, Mras, Phf21a, Gpbp1l1, Nab2, Sgk3, Foxc1, Prps1,
## Gm26829, Hexa, Ucp2, Marcksl1, Axin2, Taf7l, Ptpn14, Pbx1, Tmem108, Capn11,
## Gm48170, Akap12, Cox4i2, Tshz1, Lockd, Ngfr, Cdc42ep3, Acot1, Sox15, Rsrp1,
## Sec1, Fam213a, Pramef8, Fbxo36, Capg, Ub  
  ## Warning: Not all features provided are in this Assay object, removing the
## following feature(s): Pbld1, Srgn, Lefty1, H19, Olfr1369-ps1, Zscan4-ps2,
## Gm4027, Tmem80, Fry, Gm8994, Olfr889, Ptcra, Agl, Cyp4f14, Gm26870, Gm15879,
## Gm29666, Aldh1a3, Gm21761, Gm5039, Zscan4c, Nlrc3, Lgals1, Inhba, Gm29773,
## AF067061, Retn, Tagln, Noa1, BC147527, Gm45184, Gm8332, Foxq1, Zscan4d, S100a4,
## Fabp3, Lhfpl4, Wdcp, Plk4, Serpinh1, Mcpt8, Gm43409, Slamf1, Acta2, Cryab,
## Gm21762, S100a6, Alox8, Col4a1, Tcstv3, Spry2, Olfr1423, Zscan4-ps3, Ctsl,
## Rhox6, S100a3, Gm4340, Spp1, Vim, Lefty2, Cdkn1c, Gm26737, Spata31d1a, Xaf1,
## Dab2, Gata6, Usp17lc, BC080695, Rrm2b, AC160336.1, Thbs1, Gm45509, Bgn, Igfbp7,
## Meg3, Gfod2, Gm2016, 4930548H24Rik, Amn, Col4a2, Timp2, Tmsb4x, Aqp8, Id3,
## Hmga2, Gm2022, Ccnd2, Trh, Grem1, Atg2a, A330069E16Rik, Dqx1, P3h3, Id1, Gm428,
## Xist, Lama1, Aebp1, Hist1h2bc, Lgals4, Gm13075, Spata7, P2rx4, Rhox5, Unc13d,
## Actb, Krt18, A930014E10Rik, Fst, 1010001N08Rik, AC163720.3, Sox17, Tc2n, Alpk2,
## Mt2, Igfbp4, Txnip, Zfp352, Fbln2, Dkk1, Gm26909, Twist2, Gm48754, Lrpap1,
## Cavin2, Rpl29, 6330410L21Rik, Krt8, Fgfbp1, Apoe, Tm4sf1, Stmn2, Sfn, Gpx3,
## Usp17la, U90926, A330032B11Rik, Gm10457, Fam25c, Lgals3, Krt7, Fbp2, Lamb1,
## Ifitm1, Gng11, Tpm2, Gm13119, Tpm1, Calml4, Cyr61, 9330136K24Rik, Ccser1, Sparc,
## Prex2, Bc1, Gm26710, Ccl7, Ifitm3, Fgf1, Cited4, Gm47510, Tulp3, Csf1, P4ha2,
## Inpp4b, Col1a1, Rgs2, Obox3, Dkk2, Comt, Fcgr3, Zscan4-ps1, Hspa5, Slc16a6,
## Pim1, Krtdap, Lgi1, Dennd5b, Serpine1, Hbegf, Lox, Oaf, AC125149.3, Ddit4,
## Gm48799, Cald1, Glipr2, Gm26782, Mylpf, Tpm4, Gm48610, Limch1, 1600025M17Rik,
## 5430402O13Rik, Gm13078, Slc7a11, Sdc4, Amt, Hmox1, AU019990, Serpinb2, Ngb,
## Ubtfl1, Foxr1, Nupr1, Siglec1, Ppbp, Pyy, Fxyd3, Gm11238, Sp140, Rimklb,
## Calcoco2, Rfpl4b, Rpl39l, Cbx3, AC140186.1, Neat1, Ccno, Filip1l, B020004J07Rik,
## Dnajb9, Lmx1a, C730034F03Rik, Ccne1, Il11, Pth1r, Pou3f1, S100a13, Dcdc2c,
## Gm48764, Cd63, Gm12794, Prrx1, Pld1, Ptgs2, Gm38947, Ascl2, Slc39a1, Gm26772,
## Chpt1, Gm26764, Sct, Ccl20, Hist1h2ap, Nfkbia, Pdgfrl, Phlda1, A530040E14Rik,
## Ccl3, B2m, Ctgf, Sp110, Shkbp1, Hs3st1, Ier3, Car2, Gm8953, Gadd45g, Bex6,
## Cxcl1, Gsn, Gm11487, Ercc4, Gm20625, H2-T23, Zfp57, Dlg1, Cubn, Atf2, Usp26,
## 4933440M02Rik, Gm43263, Dnajb14, BC028528, Gata4, Krt17, Terf2ip, Myl9, Fam46b,
## Alppl2, Tmem252, Gm16368, Ngf, Runx1, Enah, Fth1, Klf2, Ccl2, Tmem92, Dppa3,
## Zar1, Cd44, Ldhb, Ube2c, Nexn, Adcy2, Epop, 1700013H16Rik, Rasl11a, Id2, Ncoa3,
## H1f0, Rps24, Gpx2, Gjb3, Hsp90aa1, Cited2, Parvb, Fam46a, Plk3, Cbr3, Rab33b,
## Sox4, Avpi1, B020031M17Rik, Igfbp2, Mafb, Phkg1, Scamp1, Fos, Spink1, S100a11,
## Dazl, 1810062G17Rik, Phlda2, Plac8, Serpinb5, Pttg1, Gm2056, Dpys, Rep15,
## Gm10696, Lrrc32, Npm2, Plaur, Gm21731, Gm7102, Il1rl1, Gm12280, Gpx4, Cav1,
## Tfap2c, Gabarapl2, Stra8, Oasl1, Gsto1, Klf4, Slc15a1, Ccdc80, Gm10827, Lcp1,
## Ifit1, Crip1, Jun, Gmcl1, Aqp3, Junb, Arhgef26, A530032D15Rik, Marveld1, Krt19,
## Jag1, Krt42, Anxa2, Snai1, Tacr3, Tiparp, Timp1, Gm35339, Psmb8, Rnf128, Ctss,
## Hspa1a, Col5a2, Axl, Ooep, Aard, AU022751, Chrm3, Ndrg1, Slc39a10, Gadd45a,
## Lrp2, Pank3, Sgk1, Slc34a2, 2200002D01Rik, Anxa1, Nodal, Rhox9, Gm20442,
## 0610040J01Rik, AC191865.2, Gm46332, Itm2b, Htra1, G0s2, Tcl1, Myo1f, Cidea,
## Arg2, Fgf3, Atf4, Smagp, Col1a2, Adm, Itpr2, Sfmbt2, Spry4, Ifi27l2a, Csrnp3,
## Gm28940, H2-D1, Rasgrp2, Fbxo15, Gm12800, Insm1, Cdkn2a, Tnfsf12, Dusp1,
## Gm21269, Pirb, Olfr881, Tinagl1, Cxcl5, Glrx2, Hmgb3, Gbp2b, Platr3, Glul,
## Gm9125, Car4, Cpsf4l, Spz1, Exosc2, Msc, Chac1, Tmem176b, Zfp296, Cst3, Rhox13,
## Mbd5, Gm15627, Isg15, Cd47, AC158554.1, AC168977.1, Podxl, 9530059O14Rik, Irgm1,
## Tdpoz1, Pnp, Gm16233, Perp, Zfp809, Gm5788, Bnip3, Apobec2, Eda, Rbm15, Slc28a3,
## Olfr376, Skil, Ahnak2, Oas1e, Ly6a, Zfp428, Clmn, Slc24a5, Ccnd1, Ppp1r15a,
## Nefl, Pla2g12b, Arhgdib, Anxa5, Gbx2, Drr1, Khdc3, Sesn2, Malat1, 2610528J11Rik,
## Tcl1b2, 1600010M07Rik, Endov, Ccdc163, Foxa2, Klf6, Mycn, Socs3, Nr2f2, Hook2,
## Utp11, Dok2, Ubald2, Wnt4, Wfdc2, Wtap, Fxyd6, Cdx2, Fam186b, Fabp5, S100a10,
## Nat8f2, Ebf1, Ckb, Hsp90b1, Ythdf2, Antxr1, Cyba, Tdrd12, Tesc, Bbs4, Hmgn5,
## Lmo7, Mecom, Sept1, Nkx6-2, Zfp622, Gm48362, Cldn7, Crygs, Cenpf, Cd24a,
## Rbbp6, Amot, Bspry, Bend5, 1700007K13Rik, G2e3, Errfi1, Cnn2, 3300005D01Rik,
## 1700097N02Rik, Obox6, Tspan8, Tubb3, Gm10687, Pfn1, Emp1, Cstb, Prrc1, Fam124a,
## Mcrip2, Srpk1, Slit2, Cdc20, Pdgfra, Tbx3, Exoc1, Sapcd1, Gm49016, Zfp560, Ghr,
## Prtg, Edn2, Dppa2, Upf1, Snhg18, Glyat, Mad2l1bp, Fhl2, Hspb1, Zfand5, Il1rn,
## Slc7a3, Eif4e1b, Kdm5b, Plin2, Rhoc, Mt4, Pla2g1b, Mthfd2, Pdia6, Cst7, Bmp4,
## Fam151a, Srxn1, Fosl1, Arl4d, Sema3c, Ninj1, H2-M2, Mphosph6, Fam162a, Tsc22d1,
## Ccl5, Fam109a, Wnt6, Gm48168, Rbp7, Nrp1, Fundc1, Peg10, Pnrc1, Gm4858, Spesp1,
## Eva1a, Klf17, Klhl13, Col27a1, Jund, Gm28578, Plet1, Prrx2, Tnc, Hes1, Scaf11,
## Dkkl1, Il6ra, Rnf11, Cdh11, Urah, 2310040G24Rik, Crlf1, B930036N10Rik, Bbx,
## Wnt7b, Foxo3, Cldn6, Ereg, Gng12, Noct, 4933434E20Rik, Pde6a, Fam110c, Slc29a1,
## Cab39, Parp12, Ttc30b, Dpysl3, Folr1, Notch2, Flrt3, Loxl3, Fn1, Emb, Sag, Gjb5,
## Stk35, Gpat3, Fam83d, Lgmn, Cabp1, Rrn3, Dynll2, Egr1, Abhd6, Tmprss2, Fxyd5,
## Clu, Alcam, Anxa3, Fdft1, Klk7, Adra2b, Nfam1, Timp3, Erf, Gapdh, Eprn, Slco3a1,
## Cpeb1, Znhit3, 1190005I06Rik, Sox9, Fosb, Pmaip1, Slbp, Smad7, Rrm2, Creb3l2,
## Hspb2, Dppa1, Epcam, Muc13, Cebpd, Taok3, Gm36266, Cebpb, Gbp2, Jam2, Jpt1,
## Arl4c, Paip2b, Pim2, Irf7, Msmo1, Npy, H1fx, Spats2l, Tppp3, Kctd3, Nrn1, Ccnb1,
## Dcc, Slc27a2, AU015836, Lef1, Sox7, Dppa5a, Pdgfa, Relb, Sfrp1, Ifit3, Gm13964,
## Jam3, Marcks, Nusap1, Flt1, Tfec, Ccnd3, Ldlr, Tdh, Foxp1, Lhfpl2, Dis3l, Cd9,
## Fgf7, Clic1, Pcgf2, Akr1c21, Zfp516, Cyp26a1, Phactr1, Anks4b, Ctnnal1, Gm2115,
## Lsp1, Ptp4a3, Ung, Reep5, Gata1, Insig1, Prr13, Fstl1, Slc10a1, Rgs16, Lgals9,
## Mrpl9, Fmr1nb, Crct1, Klf5, Slc40a1, Bfsp1, Mdm4, Atpaf2, Lepr, Nup98, Hist1h1b,
## Areg, Nnmt, Gm5, Hmces, Hmgcr, Mymx, Crabp2, Pdzd3, Pfas, Gm16222, Myef2,
## Gadd45b, Alkbh5, Fgf4, Lrrc8c, Plcxd3, Pim3, Ptpn22, Nkx6-3, Actg1, Alpk3,
## Wdr34, Clic5, Bhlhe40, Stard10, Vmn1r15, Btg1, Tubb6, 4930550L24Rik, Map1b,
## Tpbgl, Ddr2, 4933408N05Rik, Col5a1, Ddah1, Ier5, Ncam1, Fbxo34, Gm9, Usp17lb,
## Rps15a, Myl4, Tagln2, Tuba1a, Pglyrp1, Bex1, Gpx8, Arap2, Arl6ip1, Gm16755,
## Socs2, Slc39a4, Herpud1, Slc48a1, Ddit3, 2810474O19Rik, Nectin4, Baz1a, Gsta4,
## Rhox1, Tmem37, Kdm6b, 0610005C13Rik, Zc3h6, Cenpa, Apbb1ip, Abhd2, Tfpi, Pitx2,
## Usp18, Gast, Ctf2, Glrx, Ifit3b, Ppig, Adam12, Brd2, Tuba1c, Tob1, Snhg11, Cks2,
## Gm11232, Mbnl2, Cnr2, Akr1c19, Gabra1, Dusp9, Upp1, Calca, Sat1, Bhlha15, Cmtm7,
## Atrx, Gnas, Mc1r, Rab3il1, Cxcl16, 3830417A13Rik, Kdelr3, Crip2, Scd2, Dnmt3b,
## Maml3, Serpina3g, Spink10, Fthl17c, Tuba3a, Dnajc3, Ehmt2, Thy1, Hsf2, Ptma,
## Tpo, Soat1, Csrp1, Prc1, Edn1, Ptpre, Nasp, Zp3, Rps12, Mpzl1, Cltb, Crim1,
## Stmn1, Rnf168, Unc5c, Trib3, Incenp, Icam1, Gm16104, Psap, Ldoc1, Esd, Gm5463,
## Dnajc21, Nub1, Dap, Spic, Apela, Uchl1, Lamc1, Elf1, Platr31, Platr5, Man2b1,
## Igfbp3, Pip5k1a, Sh3kbp1, Calr, Tmcc3, Bcl2l11, Nav1, Adgrl3, Trpm6, Sdc1,
## Ampd3, H2-K1, P4ha1, Egln3, Gata2, Pcgf5, Aurka, Ptgr1, Asns, Tacstd2, Msx2,
## Ppia, Tspan1, Nr5a2, Tapbpl, Pdcd4, Sertad1, Ankrd37, Sf3b1, Mpped2, Gm2694,
## Foxd3, Epha4, Ube2s, Iqcb1, Oasl2, Fermt1, Dst, Gal, Pnma5, Mast4, Sin3b, L1td1,
## Rpl22l1, Tgfb3, Mdga2, Apoc1, Ctsz, Tmem30c, Slc38a2, Pmepa1, Tnfrsf12a, Sms,
## Cotl1, Xpnpep2, Rps25, H2-M5, Klf3, Pcdh17, Hmgn3, Rap2b, Crb3, Vax2, Rbp1,
## Pgam2, Ypel2, Synm, Ceacam1, Phldb2, Bbs2, Dyrk3, Ppp1r11, Zfhx2, Eras, Irf1,
## Mt3, Selenow, Amotl2, Nptx2, Basp1, Efna4, Slc2a3, Serpinb6c, Ramp2, F2r, Esrp1,
## Kif20b, Lysmd1, Gdpd5, Bbc3, Pola2, Pdzd2, Sh3bgrl, Cda, Arhgap25, Dstn, Galnt6,
## Slc38a4, Dlk1, Mael, Patl2, Efna3, Lpar6, Mirg, Ahnak, Aass, Cenpe, Pmp22,
## Fgfr3, Sycp1, Hsd17b14, Fam46c, Car7, Ticrr, Mlana, Sqle, Rpsa, Lhfp, Ovol2,
## Cpe, Elavl3, Lat2, Zfp553, Cited1, Slc7a7, Msn, Calm1, Slc7a14, Pcbd1, Zbtb10,
## Pou6f2, Manba, 9530053A07Rik, Gas6, Grasp, Elf3, Nrg1, Abtb2, C8b, Dcaf12l1,
## Nefh, Chd7, Cdc42ep5, Eomes, Mras, Phf21a, Gpbp1l1, Nab2, Sgk3, Foxc1, Prps1,
## Gm26829, Hexa, Ucp2, Marcksl1, Axin2, Taf7l, Ptpn14, Pbx1, Tmem108, Capn11,
## Gm48170, Akap12, Cox4i2, Tshz1, Lockd, Ngfr, Cdc42ep3, Acot1, Sox15, Rsrp1,
## Sec1, Fam213a, Pramef8, Fbxo36, Capg, Ub  
  # subsetting and assign levels
TBLCESCEarlyDeve1.combined<-subset(x = TBLCESCEarlyDeve1.combined, idents = c('0','1','2','3','4','5','6','ESCs','2CLC', 'Early 2C and Zygote', 'Mid-Late 2 Cell', '4 Cell', '8 Cell', '8-16 Cell', '16 Cell', 'Early-Mid Blastocyst', 'Mid-Late Blastocyst'))
clustered_levels <- c('0','1','2','3','4','5','6','ESCs','2CLC', 'Early 2C and Zygote', 'Mid-Late 2 Cell', '4 Cell', '8 Cell', '8-16 Cell', '16 Cell', 'Early-Mid Blastocyst', 'Mid-Late Blastocyst')
levels(TBLCESCEarlyDeve1.combined) <- clustered_levels  
  av.exp.forreviewer <- AverageExpression(TBLCESCEarlyDeve1.combined)$`RNA`
matrix.av.expav.exp.forreviewer <- as.data.frame(av.exp.forreviewer)
colnames(matrix.av.expav.exp.forreviewer)[0] <- "Gene_Symbol"
matrix.av.expav.exp.forreviewer<-tibble::rownames_to_column(matrix.av.expav.exp.forreviewer, "Gene_Symbol") 
matrix.av.expav.exp.forreviewer  
 
 
 
  write_xlsx(matrix.av.expav.exp.forreviewer,"~/Desktop/Scaledexpressionmatrixforviolinplot.xlsx")  
 Repeat result from Figure S3H 
  #Repeat result from Figure S3H

# Pluripotent genes

VlnPlot(TBLCESCEarlyDeve1.combined, features = 
          c("Nr0b1","Sox2","Nanog","Tdgf1","H2afz","Tubb5")
        ,ident=c('ESCs','2CLC','0', '1', '2', '3', '4', '5', '6', 'Early 2C and Zygote', 'Mid-Late 2 Cell', '4 Cell', '8 Cell', '8-16 Cell', '16 Cell', 'Early-Mid Blastocyst', 'Mid-Late Blastocyst'),assay='integrated',pt.size=0)  
   
  VlnPlot(TBLCESCEarlyDeve1.combined, features = 
          c("Pou5f1","Nop10","Zfp42","Tcf15","Tet1","Esrrb")
        ,ident=c('ESCs','2CLC','0', '1', '2', '3', '4', '5', '6', 'Early 2C and Zygote', 'Mid-Late 2 Cell', '4 Cell', '8 Cell', '8-16 Cell', '16 Cell', 'Early-Mid Blastocyst', 'Mid-Late Blastocyst'),assay='integrated',pt.size=0)  
   
  VlnPlot(TBLCESCEarlyDeve1.combined, features = 
          c("Rest","Utf1","Tcf15","H2afx","Aes")
        ,ident=c('ESCs','2CLC','0', '1', '2', '3', '4', '5', '6', 'Early 2C and Zygote', 'Mid-Late 2 Cell', '4 Cell', '8 Cell', '8-16 Cell', '16 Cell', 'Early-Mid Blastocyst', 'Mid-Late Blastocyst'),assay='integrated',pt.size=0)  
   
  # Totipotent genes

VlnPlot(TBLCESCEarlyDeve1.combined, features = 
          c("Plk2","Cdkn1a","Zfp365","Trp53inp1","Mdm2","Btg2")
        ,ident=c('ESCs','2CLC','0', '1', '2', '3', '4', '5', '6', 'Early 2C and Zygote', 'Mid-Late 2 Cell', '4 Cell', '8 Cell', '8-16 Cell', '16 Cell', 'Early-Mid Blastocyst', 'Mid-Late Blastocyst'),assay='integrated',pt.size=0)  
   
  VlnPlot(TBLCESCEarlyDeve1.combined, features = 
          c("Ddit4l","Pdrg1","Pid1","Emp3","Dffb","Ctsb")
        ,ident=c('ESCs','2CLC','0', '1', '2', '3', '4', '5', '6', 'Early 2C and Zygote', 'Mid-Late 2 Cell', '4 Cell', '8 Cell', '8-16 Cell', '16 Cell', 'Early-Mid Blastocyst', 'Mid-Late Blastocyst'),assay='integrated',pt.size=0)  
   
  VlnPlot(TBLCESCEarlyDeve1.combined, features = 
          c("Ctsd","Cd81","Mt1","Gm5662","Gm8300")
        ,ident=c('ESCs','2CLC','0', '1', '2', '3', '4', '5', '6', 'Early 2C and Zygote', 'Mid-Late 2 Cell', '4 Cell', '8 Cell', '8-16 Cell', '16 Cell', 'Early-Mid Blastocyst', 'Mid-Late Blastocyst'),assay='integrated',pt.size=0)  
   
  VlnPlot(TBLCESCEarlyDeve1.combined, features = 
          c("H2afz","Nop10","Tdgf1","Tubb5","Nr0b1","Zfp42")
        ,ident=c('ESCs','2CLC','0', '1', '2', '3', '4', '5', '6', 'Early 2C and Zygote', 'Mid-Late 2 Cell', '4 Cell', '8 Cell', '8-16 Cell', '16 Cell', 'Early-Mid Blastocyst', 'Mid-Late Blastocyst'),assay='integrated',pt.size=0)  
   
 


 

 

 

 

 


 
 

 
 
