# Supplementary material for: Comparative Analyses of Single-Cell Transcriptomic Profiles between In Vitro Totipotent Blastomere-like Cells and In Vivo Early Mouse Embryonic Cells
Source: Cells. 2021 Nov 10;10(11):3111. doi: 10.3390/cells10113111 (PMC8621967; doi:10.3390/cells10113111)
Supplement: Supplementary file 1 [file cells-10-03111-s001.zip › Sup file/R code/2.0-Repeat-genome-analysis.html]

2.0-Repeat-genome-analysis.knit


Merging TBLC repetitive genes with coding genes matrix based on cell barcodes

```
# Load relevant packages
library(Seurat)
```

```
## Attaching SeuratObject
```

```
library(dplyr)
```

```
## 
## Attaching package: 'dplyr'
```

```
## The following objects are masked from 'package:stats':
## 
##     filter, lag
```

```
## The following objects are masked from 'package:base':
## 
##     intersect, setdiff, setequal, union
```

```
library(Matrix)
library(ggplot2)
library(cowplot)
library(patchwork)
```

```
## 
## Attaching package: 'patchwork'
```

```
## The following object is masked from 'package:cowplot':
## 
##     align_plots
```

```
library(writexl)
```

```
# loading the TBLC data
TBLCCountMatrix <- read.csv("~/Desktop/Internship/PlaB data/SCTBLCcount.csv")
```

```
# loading the repeat TBLC data
RepeatTBLC.data <- Read10X(data.dir="~/Desktop/Internship/SC-repeat/SLP-TBLC/filtered_feature_bc_matrix")
```

```
RepeatTBLC.matrix<-as.data.frame(RepeatTBLC.data)
```

```
a<-substr(colnames(TBLCCountMatrix),1,15)
b<-substr(colnames(RepeatTBLC.matrix),1,15)
c <- which(a %in% b)
d1<-TBLCCountMatrix[,(c)]
colnames(d1)<-substr(colnames(d1),1,15)
```

```
#Repeat count matrix
c1 <- which(b %in% a)
d2<-RepeatTBLC.matrix[,(c1)]
colnames(d2)<-substr(colnames(d2),1,15)
d3 <- rbind(d1,d2)
```

```
TBLCwRepeat <- CreateSeuratObject(counts = d3, project = "RepeatTBLC")
```

```
## Warning: Feature names cannot have underscores ('_'), replacing with dashes
## ('-')
```

```
#QC control
# 1.number of unique genes detected in each cell
# 2.total number of molecules detected in each cell (strongly correlated with point 1)
# 3.the percentage of reads that map to the mitochondrial genome - PercentageFeature calculates the percentage of counts originating from a set of features
TBLCwRepeat$"percent.mt" <- PercentageFeatureSet(TBLCwRepeat, pattern = "^mt-")
# Visualize QC metrics as a violin plot
VlnPlot(TBLCwRepeat, features = c("nFeature_RNA", "nCount_RNA", "percent.mt"), ncol = 3)
```

```
#Normalizing Data: normalizes the feature expression measurements for each cell by the total expression (all genes a cell expresses), multiplies this by a scale factor (10,000 by default), and log-transforms the result. Normalized values are stored in TBLC[["RNA"]]@data.
#Normalized value stored here   TBLC$"RNA"@data
TBLCwRepeat <- NormalizeData(TBLCwRepeat, normalization.method = "LogNormalize", scale.factor = 10000)

#Identification of highly variable features (feature selection) - with 2000 variable features per dataset
TBLCwRepeat<- FindVariableFeatures(TBLCwRepeat, selection.method = "vst", nfeatures = 2000)
TBLCwRepeat
```

```
## An object of class Seurat 
## 18864 features across 3815 samples within 1 assay 
## Active assay: RNA (18864 features, 2000 variable features)
```

```
#Next, we apply a linear transformation (‘scaling’) that is a standard pre-processing step prior to dimensional reduction techniques like PCA. The ScaleData function:

#Shifts the expression of each gene, so that the mean expression across cells is 0
#Scales the expression of each gene, so that the variance across cells is 1
#This step gives equal weight in downstream analyses, so that highly-expressed genes do not dominate
#The results of this are stored in TBLC[["RNA"]]@scale.data

TBLCwRepeat <- ScaleData(TBLCwRepeat)
```

```
## Centering and scaling data matrix
```

```
#Perform PCA on scaled data
TBLCwRepeat <- RunPCA(TBLCwRepeat, features = VariableFeatures(object = TBLCwRepeat),verbose=FALSE)
```

```
TBLCwRepeat <- FindNeighbors(TBLCwRepeat, dims = 1:15)
```

```
## Computing nearest neighbor graph
```

```
## Computing SNN
```

```
TBLCwRepeat  <- FindClusters(TBLCwRepeat, resolution = 0.3)
```

```
## Modularity Optimizer version 1.3.0 by Ludo Waltman and Nees Jan van Eck
## 
## Number of nodes: 3815
## Number of edges: 129608
## 
## Running Louvain algorithm...
## Maximum modularity in 10 random starts: 0.8813
## Number of communities: 6
## Elapsed time: 0 seconds
```

```
# Run non-linear dimentional reduction (UMAP/tSNE)
# If you haven't installed UMAP, you can do so via reticulate::py_install(packages =
# 'umap-learn')
TBLCwRepeat <- RunUMAP(TBLCwRepeat, dims = 1:15)
```

```
## Warning: The default method for RunUMAP has changed from calling Python UMAP via reticulate to the R-native UWOT using the cosine metric
## To use Python UMAP via reticulate, set umap.method to 'umap-learn' and metric to 'correlation'
## This message will be shown once per session
```

```
## 16:13:22 UMAP embedding parameters a = 0.9922 b = 1.112
```

```
## 16:13:22 Read 3815 rows and found 15 numeric columns
```

```
## 16:13:22 Using Annoy for neighbor search, n_neighbors = 30
```

```
## 16:13:22 Building Annoy index with metric = cosine, n_trees = 50
```

```
## 0%   10   20   30   40   50   60   70   80   90   100%
```

```
## [----|----|----|----|----|----|----|----|----|----|
```

```
## **************************************************|
## 16:13:22 Writing NN index file to temp file /var/folders/cv/cfhg7t_j5y3d34y2_81gskv00000gn/T//RtmpMhBtp4/file4ec34f61da46
## 16:13:22 Searching Annoy index using 1 thread, search_k = 3000
## 16:13:23 Annoy recall = 100%
## 16:13:23 Commencing smooth kNN distance calibration using 1 thread
## 16:13:23 Initializing from normalized Laplacian + noise
## 16:13:24 Commencing optimization for 500 epochs, with 155942 positive edges
## 16:13:28 Optimization finished
```

```
# note that you can set `label = TRUE` or use the LabelClusters function to help label
# individual clusters
DimPlot(TBLCwRepeat, reduction = "umap",label=TRUE)
```

```
# Assigning cluster names for future reference and data integration
TBLCwRepeat.cluster.ids <- c("TBLCs","TBLCs","TBLCs","TBLCs","TBLCs","MEF")
names(TBLCwRepeat.cluster.ids) <- levels(TBLCwRepeat)
TBLCwRepeat <- RenameIdents(TBLCwRepeat, TBLCwRepeat.cluster.ids)
DimPlot(TBLCwRepeat, reduction = "umap", label = TRUE, label.size=5) + NoLegend()
```

```
FeaturePlot(TBLCwRepeat, features = c("Zscan4c","Zscan4d"),min.cutoff="q3")
```

```
FeaturePlot(TBLCwRepeat, features = c("MERVL-int","MT2-Mm"),min.cutoff = "q3")
```

```
FeaturePlot(TBLCwRepeat, features = c("S100a4"),min.cutoff = "q3")
```

Merging ESC repetitive genes with coding genes matrix based on cell barcodes

```
# load ESC data
ESCCountMatrix <- read.csv("~/Desktop/Internship/ESC data/SCESCcount.csv")
```

```
# loading the repeat TBLC data
RepeatESC.data <- Read10X(data.dir="~/Desktop/Internship/SC-repeat/WT/filtered_feature_bc_matrix")
```

```
RepeatESC.matrix<-as.data.frame(RepeatESC.data)
```

```
A<-substr(colnames(ESCCountMatrix),1,15)
B<-substr(colnames(RepeatESC.matrix),1,15)
C <- which(A %in% B)
D1<-ESCCountMatrix[,(C)]
colnames(D1)<-substr(colnames(D1),1,15)
```

```
#Repeat count matrix
C1 <- which(B %in% A)
D2<-RepeatESC.matrix[,(C1)]
colnames(D2)<-substr(colnames(D2),1,15)
D3 <- rbind(D1,D2)
```

```
ESCwRepeat <- CreateSeuratObject(counts = D3, project = "RepeatESC")
```

```
## Warning: Feature names cannot have underscores ('_'), replacing with dashes
## ('-')
```

```
#QC control
# 1.number of unique genes detected in each cell
# 2.total number of molecules detected in each cell (strongly correlated with point 1)
# 3.the percentage of reads that map to the mitochondrial genome - PercentageFeature calculates the percentage of counts originating from a set of features
ESCwRepeat$"percent.mt" <- PercentageFeatureSet(ESCwRepeat, pattern = "^mt-")
# Visualize QC metrics as a violin plot
VlnPlot(ESCwRepeat, features = c("nFeature_RNA", "nCount_RNA", "percent.mt"), ncol = 3)
```

```
#Normalizing Data: normalizes the feature expression measurements for each cell by the total expression (all genes a cell expresses), multiplies this by a scale factor (10,000 by default), and log-transforms the result. Normalized values are stored in ESC[["RNA"]]@data.
#Normalized value stored here   ESCwRepeat$"RNA"@data
ESCwRepeat <- NormalizeData(ESCwRepeat, normalization.method = "LogNormalize", scale.factor = 10000)

#Identification of highly variable features (feature selection) - with 2000 variable features per dataset
ESCwRepeat <- FindVariableFeatures(ESCwRepeat, selection.method = "vst", nfeatures = 2000)

#Next, we apply a linear transformation (‘scaling’) that is a standard pre-processing step prior to dimensional reduction techniques like PCA. The ScaleData function:

#Shifts the expression of each gene, so that the mean expression across cells is 0
#Scales the expression of each gene, so that the variance across cells is 1
#This step gives equal weight in downstream analyses, so that highly-expressed genes do not dominate
#The results of this are stored in ESC[["RNA"]]@scale.data

ESCwRepeat <- ScaleData(ESCwRepeat)
```

```
## Centering and scaling data matrix
```

```
#Perform PCA on scaled data
ESCwRepeat <- RunPCA(ESCwRepeat, features = VariableFeatures(object = ESCwRepeat),nfeatures.print=2000,verbose = FALSE)
```

```
#Clustering Cells
ESCwRepeat <- FindNeighbors(ESCwRepeat, dims = 1:20)
```

```
## Computing nearest neighbor graph
```

```
## Computing SNN
```

```
ESCwRepeat <- FindClusters(ESCwRepeat, resolution = 3.4)
```

```
## Modularity Optimizer version 1.3.0 by Ludo Waltman and Nees Jan van Eck
## 
## Number of nodes: 4087
## Number of edges: 136320
## 
## Running Louvain algorithm...
## Maximum modularity in 10 random starts: 0.5249
## Number of communities: 28
## Elapsed time: 0 seconds
```

```
# Run non-linear dimentional reduction (UMAP/tSNE)
# If you haven't installed UMAP, you can do so via reticulate::py_install(packages =
# 'umap-learn')
ESCwRepeat <- RunUMAP(ESCwRepeat, dims = 1:20)
```

```
## 16:14:10 UMAP embedding parameters a = 0.9922 b = 1.112
```

```
## 16:14:10 Read 4087 rows and found 20 numeric columns
```

```
## 16:14:10 Using Annoy for neighbor search, n_neighbors = 30
```

```
## 16:14:10 Building Annoy index with metric = cosine, n_trees = 50
```

```
## 0%   10   20   30   40   50   60   70   80   90   100%
```

```
## [----|----|----|----|----|----|----|----|----|----|
```

```
## **************************************************|
## 16:14:11 Writing NN index file to temp file /var/folders/cv/cfhg7t_j5y3d34y2_81gskv00000gn/T//RtmpMhBtp4/file4ec339c96200
## 16:14:11 Searching Annoy index using 1 thread, search_k = 3000
## 16:14:12 Annoy recall = 100%
## 16:14:12 Commencing smooth kNN distance calibration using 1 thread
## 16:14:12 Initializing from normalized Laplacian + noise
## 16:14:12 Commencing optimization for 500 epochs, with 167170 positive edges
## 16:14:18 Optimization finished
```

```
DimPlot(ESCwRepeat, reduction = "umap", label = TRUE)
```

```
# Assigning cluster names for future reference and data integration
ESCwRepeat.cluster.ids <- c("ESCs","ESCs","ESCs","ESCs","ESCs","ESCs","ESCs","ESCs","ESCs","ESCs","ESCs","ESCs","ESCs","ESCs","ESCs","ESCs","ESCs","ESCs","ESCs","ESCs","ESCs","ESCs","ESCs","ESCs","ESCs","ESCs","ESCs","2CLC")
names(ESCwRepeat.cluster.ids) <- levels(ESCwRepeat)
ESCwRepeat <- RenameIdents(ESCwRepeat, ESCwRepeat.cluster.ids)
DimPlot(ESCwRepeat, reduction = "umap", label = TRUE) + NoLegend()
```

```
FeaturePlot(ESCwRepeat, features = c("Zscan4c","Zscan4d"),min.cutoff="q3")
```

```
FeaturePlot(ESCwRepeat, features = c("MERVL-int","MT2-Mm"),min.cutoff = "q3")
```

```
FeaturePlot(ESCwRepeat, features = c("S100a4"),min.cutoff = "q3")
```

Integrate the two datasets (TBLCs and ESCs with repetitive genes)

```
TBLCwRepeat <- FindNeighbors(TBLCwRepeat, dims = 1:15)
```

```
## Computing nearest neighbor graph
```

```
## Computing SNN
```

```
TBLCwRepeat  <- FindClusters(TBLCwRepeat, resolution = 0.5)
```

```
## Modularity Optimizer version 1.3.0 by Ludo Waltman and Nees Jan van Eck
## 
## Number of nodes: 3815
## Number of edges: 129608
## 
## Running Louvain algorithm...
## Maximum modularity in 10 random starts: 0.8399
## Number of communities: 8
## Elapsed time: 0 seconds
```

```
# Run non-linear dimentional reduction (UMAP/tSNE)
# If you haven't installed UMAP, you can do so via reticulate::py_install(packages =
# 'umap-learn')
TBLCwRepeat <- RunUMAP(TBLCwRepeat, dims = 1:15)
```

```
## 16:14:23 UMAP embedding parameters a = 0.9922 b = 1.112
```

```
## 16:14:23 Read 3815 rows and found 15 numeric columns
```

```
## 16:14:23 Using Annoy for neighbor search, n_neighbors = 30
```

```
## 16:14:23 Building Annoy index with metric = cosine, n_trees = 50
```

```
## 0%   10   20   30   40   50   60   70   80   90   100%
```

```
## [----|----|----|----|----|----|----|----|----|----|
```

```
## **************************************************|
## 16:14:23 Writing NN index file to temp file /var/folders/cv/cfhg7t_j5y3d34y2_81gskv00000gn/T//RtmpMhBtp4/file4ec3502310aa
## 16:14:23 Searching Annoy index using 1 thread, search_k = 3000
## 16:14:24 Annoy recall = 100%
## 16:14:25 Commencing smooth kNN distance calibration using 1 thread
## 16:14:25 Initializing from normalized Laplacian + noise
## 16:14:25 Commencing optimization for 500 epochs, with 155942 positive edges
## 16:14:30 Optimization finished
```

```
# note that you can set `label = TRUE` or use the LabelClusters function to help label
# individual clusters
DimPlot(TBLCwRepeat, reduction = "umap",label=TRUE)
```

```
# Assigning cluster names for future reference and data integration
TBLCwRepeat.cluster.ids <- c("0","1","3","2","4","5","6","MEF")
names(TBLCwRepeat.cluster.ids) <- levels(TBLCwRepeat)
TBLCwRepeat <- RenameIdents(TBLCwRepeat, TBLCwRepeat.cluster.ids)
DimPlot(TBLCwRepeat, reduction = "umap", label = TRUE, label.size=5) + NoLegend()
```

```
table(TBLCwRepeat@active.ident)
```

```
## 
##    0    1    3    2    4    5    6  MEF 
## 1057  825  592  511  306  247  203   74
```

```
# merging TBLC and ESC data
ESCTBLCwRepeat<-merge(TBLCwRepeat,ESCwRepeat)
```

```
## Warning in CheckDuplicateCellNames(object.list = objects): Some cell names are
## duplicated across objects provided. Renaming to enforce unique cell names.
```

```
# splitting object identify for downstream cluster analysis
ESCTBLCwRepeat1 <- SplitObject(ESCTBLCwRepeat, split.by = "orig.ident")
ESCTBLCwRepeat1
```

```
## $RepeatTBLC
## An object of class Seurat 
## 19718 features across 3815 samples within 1 assay 
## Active assay: RNA (19718 features, 0 variable features)
## 
## $RepeatESC
## An object of class Seurat 
## 19718 features across 4087 samples within 1 assay 
## Active assay: RNA (19718 features, 0 variable features)
```

```
# normalize and identify variable features for each dataset independently
ESCTBLCwRepeat1 <- lapply(X = ESCTBLCwRepeat1, FUN = function(x) {
    x <- NormalizeData(x)
    x <- FindVariableFeatures(x, selection.method = "vst", nfeatures = 2000)
})

# integrating data by integrating 2 seurat objects as input
ESCTBLCwRepeat1.anchors <- FindIntegrationAnchors(object.list = ESCTBLCwRepeat1, dims = 1:20)
```

```
## Computing 2000 integration features
```

```
## Scaling features for provided objects
```

```
## Finding all pairwise anchors
```

```
## Running CCA
```

```
## Merging objects
```

```
## Finding neighborhoods
```

```
## Finding anchors
```

```
##  Found 11282 anchors
```

```
## Filtering anchors
```

```
##  Retained 4012 anchors
```

```
ESCTBLCwRepeat1.combined <- IntegrateData(anchorset = ESCTBLCwRepeat1.anchors, dims = 1:20)
```

```
## Merging dataset 1 into 2
```

```
## Extracting anchors for merged samples
```

```
## Finding integration vectors
```

```
## Finding integration vector weights
```

```
## Integrating data
```

```
DefaultAssay(ESCTBLCwRepeat1.combined ) <- "integrated"

# Run the standard workflow for visualization and clustering
ESCTBLCwRepeat1.combined  <- ScaleData(ESCTBLCwRepeat1.combined, verbose = FALSE)
ESCTBLCwRepeat1.combined  <- RunPCA(ESCTBLCwRepeat1.combined, verbose = FALSE)
# TSNE and Clustering
ESCTBLCwRepeat1.combined  <- RunTSNE(ESCTBLCwRepeat1.combined, reduction = "pca", dims = 1:20, check_duplicates = FALSE)
```

```
# To visualize the two conditions side-by-side, we can use the split.by argument to show each condition colored by cluster
DimPlot(ESCTBLCwRepeat1.combined, reduction = "tsne",group.by = "orig.ident")
```

```
DimPlot(ESCTBLCwRepeat1.combined, reduction = "tsne")
```

```
FeaturePlot(ESCTBLCwRepeat1.combined, features = c("Zscan4c","Zscan4d"), split.by = "orig.ident", max.cutoff = 3,reduction = "tsne",
    cols = c("grey","red"),min.cutoff = "q5")
```

```
FeaturePlot(ESCTBLCwRepeat1.combined, features = c("MERVL-int","MT2-Mm"), split.by = "orig.ident", max.cutoff = 3,reduction = "tsne",
    cols = c("grey","red"),min.cutoff = "q15")
```

```
# Another non-overlapping population is MEF as viewed under MEF marker Sp100a4
FeaturePlot(ESCTBLCwRepeat1.combined, features = c("S100a4"), split.by = "orig.ident", max.cutoff = 3,reduction = "tsne",
    cols = c("grey","red"),min.cutoff = "q5")
```

```
# assign levels
ESCTBLCwRepeat1.combined_levels <- c('0', '1', '2', '3', '4', '5', '6','ESCs','2CLC','MEF')
levels(ESCTBLCwRepeat1.combined) <- ESCTBLCwRepeat1.combined_levels
```

```
VlnPlot(ESCTBLCwRepeat1.combined, features = c("Zscan4c","Zscan4d"),ident=c('ESCs','2CLC','0', '1', '2', '3', '4', '5', '6'),assay='integrated',pt.size=0)
```

```
VlnPlot(ESCTBLCwRepeat1.combined, features = c("MERVL-int","MT2-Mm"),ident=c('ESCs','2CLC','0', '1', '2', '3', '4', '5', '6'),assay='integrated',pt.size=0)
```
